# Nine New Glycosylated Compounds from the Leaves of the Medicinal Plant *Malus hupehensis*

**DOI:** 10.3390/molecules29225269

**Published:** 2024-11-07

**Authors:** Lin-Lin Yuan, Yi Wang, Guo-Kai Wang, Ji-Kai Liu

**Affiliations:** 1 Anhui Province Key Laboratory of Bioactive Natural Products, School of Pharmaceutical Sciences, Anhui University of Chinese Medicine, Hefei 230012, China; yuanlinlin44@ahtcm.edu.cn; 2 Genpact, 1155 Avenue of the Americas 4th Fl, New York, NY 10036, USA; ywangcathy@gmail.com

**Keywords:** *Malus hupehensis* (Pamp.) Rehd., rosaceae, dihydrochalcone glycosides, NO production activity, α-glucosidase inhibitor

## Abstract

Nine new compounds (**1**–**9**), including four dihydrochalcone glycosides, two dibenzofuran glycosides, and two biphenyl glycosides, were isolated from the leaves of the medicinal plant *Malus hupehensis* collected in Shennongjia Forestry District (Hubei, China). Their structures were elucidated by comprehensive spectroscopic techniques, including HRESIMS and NMR spectra. All compounds were tested by preliminary biological evaluation for their α-glucosidase inhibitory and NO production activities. Compound **4** was found to show significant inhibitory activity against NO production in LPS-activated RAW 264.7 macrophage cells with an IC_50_ value of 29.60 μM, and compounds **3** and **4** were found to exhibit potent α-glucosidase inhibition with IC_50_ values of 44.17 and 60.15 μM, respectively. This work represents the first report of the diverse glycosides from the plant *Malus hupehensis*. It expands our understanding of the secondary metabolites of this medicinal plant and lays the foundation for the study of the bioactive principles of the ethnic hypoglycemic medicinal plant.

## 1. Introduction

*Malus hupehensis* (Pamp.) Rehder, a tree within the Rosaceae family and Malus genus, commonly known as ‘Tea crabapple’, is widely distributed throughout southern China. It has a long history of use as a healthy tea or herbal medicine for treating hyperglycemia, particularly in the Tujia ethnic minority in Hubei Province. It was approved by the National Health Commission of China as a novel food supplement in 2014 [1]. Previously, pharmacological studies have shown that *M. hupehensis* exerts biological properties, such as antioxidant [2,3], antithrombotic [4], anticancer [5], hepatic protection [6], lowering cholesterol levels [7,8], and so on. Recently, research on *M. hupehensis* has gained increasing attention in the fields of food and pharmaceutical products.

Diabetes, a prevalent and severe chronic disease, ranks among the top 10 causes of mortality in adults and exerts a profound negative impact on individuals, families, and society at large. Owing to shifts in lifestyle and living conditions, the prevalence of diabetes has been steadily escalating in our nation in recent years. According to data from the International Diabetes Federation (IDF), there were 536 million people with diabetes globally in 2021, and this number is projected to rise to 783 million by 2045 [9]. The exploration of safe and preventative hypoglycemic medications is crucial, given the rising prevalence of diabetes in China.

There are three main types of diabetes: type 1 diabetes mellitus (T1D), type 2 diabetes mellitus (T2D), and gestational diabetes mellitus (GDM) [10]. Patients with type 2 diabetes (T2D) make up the majority of them, which is the focus of the current study. Acarbose, voglibose, and miglitol, three commercially available α-glucosidase inhibitors, effectively control postprandial hyperglycemia. However, frequent use has adverse effects on the gastrointestinal system [11]. Therefore, the quest for novel α-glucosidase inhibitors from natural sources has captured our attention. *M. hupehensis* is used to treat type 2 diabetes with significant efficacy. Still, information on its antihyperglycemic chemical components and their biological effects remains limited. Therefore, it is necessary to investigate the active components with unique structures from *M. hupehensis* and assess their biological activities. It is especially important to consider the Chinese habit of drinking tea in health care. The plant *M. hupehensis* has a long tradition of use as a health tea. Its safety and efficacy have been evidenced in a large number of people over a long period. There is a need to study the active components of the plant related to its role in human glucose metabolism.

## 2. Results and Discussion

**Figure 1:**
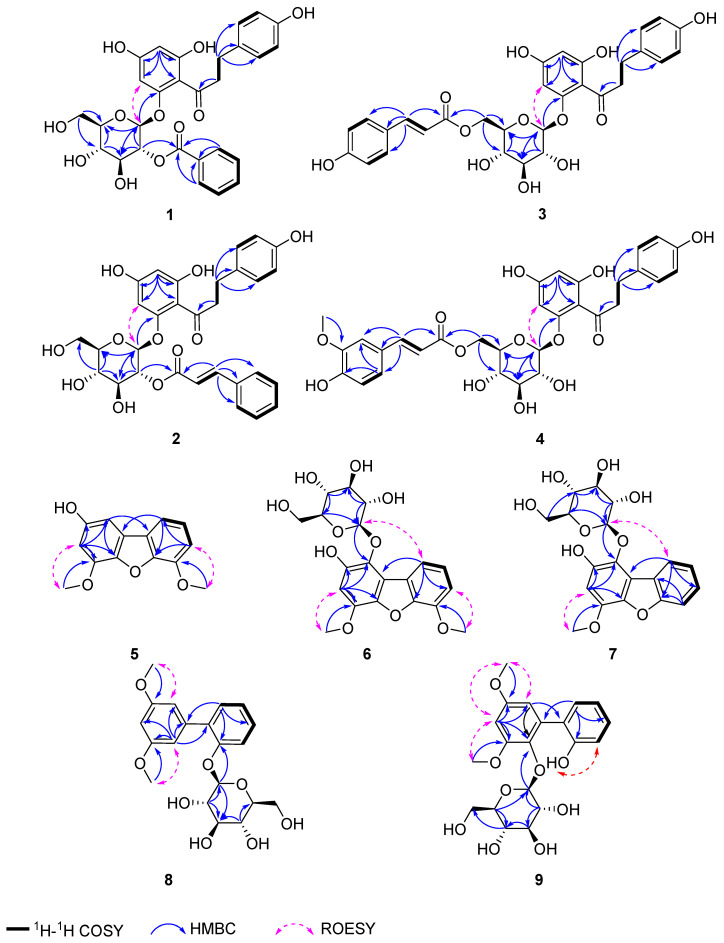
Key HMBC, ^1^H-^1^H COSY, and ROESY correlations of **1**–**9**.

Compound **1** was obtained as a light-yellow powder. The molecular formula of **1** was determined as C_28_H_28_O_11_ by HRESIMS [*m/z* 563.15247 [M + Na]^+^ (calcd. for C_28_H_28_O_11_Na, 563.15238)]. The ^1^H NMR spectrum (Table 1) showed eleven aromatic olefinic protons at *δ*_H_ 8.05 (2H, dd, *J* = 8.5, 1.2 Hz, H-2′′′, 6′′′), 7.61 (1H, t, *J* = 7.5 Hz, H-4′′′), 7.47 (2H, t, *J* = 7.8 Hz, H-3′′′, 5′′′), 6.87 (2H, d, *J* = 8.5 Hz, H-2, 6), 6.58 (2H, d, *J* = 8.5 Hz, H-3, 5), 6.18 (1H, d, *J* = 2.2 Hz, H-3′), and 5.92 (1H, d, *J* = 2.2 Hz, H-5′). The ^13^C NMR and DEPT spectra (Table 2) exhibited signals for twenty-eight carbon atoms, including three methylenes [*δ*_C_ 62.3 (C-6″), 46.6 (C-α), and 30.0 (C-β)], sixteen methines [eleven aromatic carbons *δ*_C_ 134.6 (C-4′′′), 130.9 (C-2′′′, 6′′′), 130.5 (C-2, 6), 129.7 (C-3′′′, 5′′′), 115.8 (C-3, 5), 98.4 (C-5′), and 95.3 (C-3′), five glucose residue carbons *δ*_C_ 99.2 (C-1″), 78.6 (C-5″), 76.5 (C-3″), 75.2 (C-2″), and 71.3 (C-4″)], and nine quaternary carbons [two carbonyl carbons *δ*_C_ 205.8 (C=O), 167.2 (C-7′′′), seven aromatic carbons *δ*_C_ 167.1 (C-6′), 165.8 (C-4′), 161.3 (C-2′), 156.2 (C-4), 133.7 (C-1), 131.1 (C-1′′′), and 106.7 (C-1′)]. The related compound to **1** was phloridzin 6″-O-benzoate [12], with the difference being the connection site of the *p*-hydroxybenzoyl moiety. This *p*-hydroxybenzoyl in compound **1** (Figure 1) was connected at the C-2″ and was confirmed by HMBC correlations of H-2″ (1H, *δ*_H_ 5.32, dd, *J* = 9.5, 8.1 Hz) and C-7′′′. The glucopyranoside was *β*-configuration on the ground of a large coupling constant (*δ*_H_ 5.49, *J* = 8.1 Hz, H-1″) of the anomeric proton. Therefore, compound **1** was identified as malahupinoside C.

**Figure 2:**
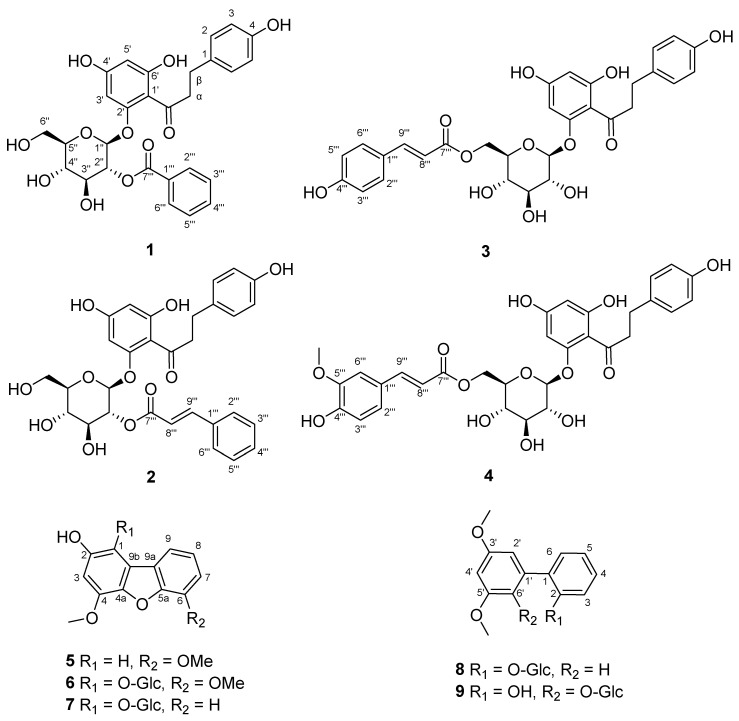
Structures of compounds **1**–**9**.

Compound **2** was obtained as a light-yellow powder. Its molecular formula of **2** was determined as C_30_H_30_O_11_ by HRESIMS [*m/z* 589.16821 [M + Na]^+^ (calcd. for C_30_H_30_O_11_Na, 589.16803)]. The ^1^H NMR spectrum (Table 1) showed signals for thirteen olefinic protons at *δ*_H_ 7.72 (1H, d, *J* = 16.0 Hz, H-9′′′), 7.58 (2H, m, H-2′′′, 6′′′), 7.40 (2H, overlapped, H-3′′′, 5′′′), 7.39 (1H, overlapped, H-4′′′), 7.01 (2H, d, *J* = 8.5 Hz, H-2, 6), 6.60 (2H, d, *J* = 8.5 Hz, H-3, 5), 6.55 (1H, d, *J* = 16.0 Hz, H-8′′′), 6.17 (1H, d, *J* = 2.2 Hz, H-3′), and 5.93 (1H, d, *J* = 2.2 Hz, H-5′). The ^13^C NMR and DEPT spectra (Table 2) revealed signals for thirty carbon atoms, including three methylenes [*δ_C_* 62.3 (C-6″), 46.6 (C-α), and 30.2 (C-β)], eighteen methines [thirteen aromatic carbons *δ_C_* 147.2 (C-9′′′), 131.7 (C-4′′′), 130.6 (C-2, 6), 130.0 (C-3′′′, 5′′′), 129.4 (C-2′′′, 6′′′), 118.5 (C-8′′′), 115.9 (C-3, 5), 98.4 (C-5′), and 95.3 (C-3′), five glucose residue carbons *δ_C_* 99.2 (C-1″), 78.6 (C-5″), 76.5 (C-3″), 74.8 (C-2″), and 71.2 (C-4″)], and nine quaternary carbons [two carbonyl carbons *δ_C_* 206.0 (C=O), 167.5 (C-7′′′), seven aromatic carbons *δ_C_* 167.2 (C-6′), 165.8 (C-4′), 161.3 (C-2′), 156.2 (C-4), 135.7 (C-1′′′), 133.8 (C-1), and 106.8 (C-1′)]. The comparison of the ^1^H and ^13^C NMR spectroscopic data (Table 1 and Table 2) of **2** revealed similar resonances to those of phloridzin 6″-O-cinnamate [13], apart from the position of cinnamate in phloridzin 6″-O-cinnamate was located from C-6″ to C-2″ in **2**. The HMBC correlations (Figure 1) between the anomeric proton at H-2″ (1H, *δ*_H_ 5.20, t, *J* = 8.2 Hz) and the carbon at C-7′′′ confirmed this change. The structure of **2** is depicted in Figure 2 and named malahupinoside D.

Compound **3** was obtained as a light-yellow powder. The molecular formula of C_30_H_30_O_12_ was established by the positive HRESIMS [M + Na]^+^ ion peak at *m/z* 605.16467 (calcd. for C_30_H_30_O_12_Na, 605.16295). The comparison of the ^1^H and ^13^C NMR spectroscopic data (Table 1 and Table 2) of **3** showed similar resonances to those of phloridzin 6″-O-cinnamate, with the only difference being the substitution of a hydroxyl moiety at C-4′′′ (*δ*_C_ 161.2). This finding was confirmed by ^1^H–^1^H COSY correlations (Figure 1) between H-2′′′/H-3′′′ and H-5′′′/H-6′′′, together with HRESIMS (Figure S15). Therefore, compound **3** was named malahupinoside E.

Compound **4** was obtained as a light-yellow powder. The molecular formula of C_31_H_32_O_13_ was established by the positive HRESIMS [M + Na]^+^ ion peak at *m/z* 635.17535 (calcd. for C_31_H_32_O_13_Na, 635.17351). The ^1^H and ^13^C NMR spectra of compound **4** (Table 1 and Table 2) were similar to those of **3**, with the main difference being the presence of an additional methoxy group at C-5′′′ (*δ*_C_ 149.3). The HMBC correlations (Figure 1) observed from H-10′′′ (*δ*_H_ 3.85, 3H, s) to C-5′′′, from H-2′′′ (1H, *δ*_H_ 7.02, dd, *J* = 8.1, 1.7 Hz) to C-4′′′ (*δ*_C_ 150.6) and C-6′′′ (*δ*_C_ 111.5) further confirmed the presence of this methoxy group. Thus, the structure of **4** was elucidated as malahupinoside F.

Compound **5** was obtained as a pale-yellow gum. Its molecular formula was determined as C_14_H_12_O_4_ from the [M + H]^+^ ion peak at *m/z* 245.08082 (calcd. for C_14_H_13_O_4_, 254.08084) in the positive HRESIMS. The ^1^H NMR spectrum (Table 3) revealed signals for two methoxy groups at *δ*_H_ 4.01 (3H, s, 6-OMe), and 3.99 (3H, s, 4-OMe), along with five aromatic methines at *δ*_H_ 7.44 (1H, dd, *J* = 7.8, 0.8 Hz, H-9), 7.22 (1H, t, *J* = 7.8 Hz, H-8), 7.03 (1H, dd, *J* = 7.8, 0.8 Hz, H-7), 6.89 (1H, d, *J* = 2.2 Hz, H-1), and 6.58 (1H, d, *J* = 2.2 Hz, H-3). The ^13^C NMR and DEPT spectra (Table 3) indicated the presence of fourteen carbon atoms, including two methoxy carbons *δ*_C_ 56.7 (4-OMe, 6-OMe), five aromatic methines carbons *δ*_C_ 124.3 (C-8), 113.7 (C-9), 110.7 (C-7), 100.6 (C-3), and 98.2 (C-1), and seven quaternary carbons *δ*_C_ 155.5 (C-2), 147.3 (C-4), 147.2 (C-6), 147.0 (C-5a), 140.9 (C-4a), 127.3 (C-9a), and 127.1 (C-9b). The ^1^H and ^13^C NMR data of **5** were similar to those of 2-hydroxy-4-methoxydibenzofuran [14], except for the presence of a carbomethoxyl group at C-6, which was confirmed by HMBC correlations (Figure 1) between H-6-OMe and C-6, together with ROESY correlated of H-6-OMe and H-7. Thus, the structure of **5** was determined to be fortuneanoside M.

Compound **6** was obtained as a pale-yellow gum. Its molecular formula was determined as C_20_H_22_O_10_ from the [M + H]^+^ ion peak at *m/z* 423.12857 (calcd. for C_20_H_23_O_10_, 423.12857) in the positive HRESIMS. The ^1^H and ^13^C NMR data of **6** (Table 3) were similar to those of **5**, except for the presence of a glucose residue. In the HMBC spectrum, a long-range correlation between the signals at *δ*_H_ 4.83, 3.78/3.73, 3.64, 3.53, 3.46, 3.26, and the carbon signals at *δ*_C_ 107.5 (C-1″), 78.4 (C-5″), 78.0 (C-3″), 75.6 (C-2″), 71.0 (C-4″), and 62.2 (C-6″) confirmed the speculation. The connection of glucose residue at C-1 (*δ*_C_ 133.0) was further supported by HMBC correlations (Figure 2) from H-1′ (1H, *δ*_H_ 4.83, d, *J* = 7.8 Hz) to C-1. Additionally, acid hydrolysis of **6** confirmed the presence of D-glucose, which was determined to be in the *β*-configuration based on the large coupling constant (*J* = 7.8 Hz) of the anomeric proton. Thus, the structure of **6** was determined to be fortuneanoside N.

Compound **7** was obtained as a pale-yellow gum. Its molecular formula was determined as C_19_H_20_O_9_ from the [M + H]^+^ ion peak at *m/z* 393.11800 (calcd. for C_19_H_21_O_9_, 393.11801) in the positive HRESIMS. The comparison of the ^1^H and ^13^C NMR spectroscopic data (Table 3) of **7** revealed similar resonances to those of **6**, apart from the absence of a methoxy group at C-6 (*δ*_C_ 112.0). This change was corroborated by ^1^H–^1^H COSY correlations (Figure 2) between H-6, H-7, H-8 and H-9. The sugar was confirmed to be *β*-D-glucose, as described in **6**. Therefore, compound **7** was named fortuneanoside O.

Compound **8** was obtained as a pale-yellow gum. Its molecular formula was determined as C_20_H_24_O_8_ from the [M + Na]^+^ ion peak at *m/z* 415.13614 (calcd. for C_20_H_24_O_8_Na, 415.13634) in the positive HRESIMS. The ^13^C and DEPT NMR spectra (Table 4) of **8** showed twenty carbons, including two methoxy groups, one methylene, twelve methines, and five aromatic quaternary carbons. The ^1^H NMR spectrum (Table 4) revealed signals for a 1,2-disubstituted aromatic ring [*δ*_H_ 7.31 (1H, overlapped, H-6), 7.30 (1H, overlapped, H-4), 7.26 (1H, dd, *J* = 8.7, 1.2 Hz, H-3), and 7.07 (1H, td, *J* = 7.4, 1.2 Hz, H-5)], a 1′,3′,5′-trisubstituted aromatic ring [*δ*_H_ 6.75 (2H, d, *J* = 2.3 Hz, H-2′, 6′), and 6.43 (1H, t, *J* = 2.3 Hz, H-4′)], two methoxy groups at *δ*_H_ 3.80 (6H, s,3′, 5′-OCH_3_). The remaining signals in the ^1^H NMR spectrum showed the presence of a glucose residue [*δ*_H_ 5.08 (1H, d, *J* = 8.0 Hz, H-1″), 3.87 (1H, dd, *J* = 12.4, 2.2 Hz, H-6″), 3.68 (1H, dd, *J* = 12.4, 5.5 Hz, H-6″), 3.44 (1H, overlapped, H-3″), 3.42 (1H, overlapped, H-5″), 3.40 (1H, overlapped, H-2″), and 3.37 (1H, m, H-4″)]. Compared to the structure of fortuneanoside C [15], the distinct difference of **8** was the absence of the hydroxyl group at C-4′ (*δ*_C_ 100.4). The ROESY correlations (Figure 2) between the protons of two methoxy groups (3′, 5′-OCH_3_) and two equivalent aromatic protons (H-2′ and H-6′), and H-4′ confirmed this change. Therefore, compound **8** was named fortuneanoside P.

Compound **9** was obtained as a pale-yellow gum. Its molecular formula was determined as C_20_H_24_O_9_ from the [M + Na]^+^ ion peak at *m/z* 431.13116 (calcd. for C_20_H_24_O_9_Na, 431.13125) in the positive HRESIMS. The ^1^H NMR spectrum (Table 4) showed typical signals for a 1,2-disubstituted aromatic ring [*δ*_H_ 7.24 (1H, d, *J* = 7.2 Hz, H-6), 7.10 (1H, d, *J* = 7.6 Hz, H-4), 6.85 (1H, d, *J* = 8.0 Hz, H-3), and 6.76 (1H, d, *J* = 7.2 Hz, H-5)], a 1′,2′,3′,5′-trisubstituted aromatic ring [*δ*_H_ 6.59 (1H, d, *J* = 2.0 Hz, H-4′), and 6.29 (1H, d, *J* = 2.0 Hz, H-2′)], two methoxy groups at *δ*_H_ 3.80 (5′-OCH_3_) and *δ*_H_ 3.71 (3′-OCH_3_). The remaining signals in the ^1^H NMR spectrum showed the presence of a glucose residue [*δ*_H_ 4.68 (1H, d, *J* = 7.6 Hz, H-1″), 3.51 (1H, d, *J* = 11.7 Hz, H-6″), 3.29 (1H, overlapped, H-6″), 3.05 (1H, t, *J* = 8.6 Hz, H-3″), 2.93 (1H, overlapped, H-4″), 2.90 (1H, overlapped, H-2″), and 2.89 (1H, m, H-5″)]. Compared to the structure of fortuneanoside B [15], the distinct difference of **9** was the absence of the hydroxyl group at C-4′ (*δ*_C_ 99.6). The ROESY correlations (Figure 2) between the protons of two methoxy groups (3′, 5′-OCH_3_) and H-4′confirmed this change. In addition, in **9** there was the absence of the methoxy group at C-6 (*δ*_C_ 132.0) and the presence of an extra hydroxy group at C-2 (*δ*_C_ 154.2). These changes were confirmed by the HMBC correlations (Figure 2) from H-6 to C-1′ (*δ*_C_ 133.5), C-2, and C-4 (*δ*_C_ 128.1), and the ^1^H–^1^H COSY correlations (Figure 2) between H-3, H-4, H-5 and H-6, and the ROESY correlation between H-6 and H-2′, along with HRESIMS data (Figure S57). The strategy previously described was used to determine the sugar configuration. Therefore, compound **9** was named fortuneanoside Q.

The six known analogues were identified as phloridzin 6″-O-benzoate (**10**) [12], phloridzin 6″-O-cinnamate (**11**) [13], 4-[3-[2-(*β*-D-Glucopyranosyloxy)-4,6-dihydroxyphenyl]-3-oxopropyl]phenyl(2*E*)-3-(4-hydroxyphenyl)-2-propenoate (**12**) [13], methylenebisphloridzin (**13**) [16], 2-Hydroxy-4-methoxydibenzofuran (**14**) [14], and 3′,5′-Dimethoxy-(1,1′-biphenyl)-3,4-diol 3-O-β-D-glucopyranoside (**15**) [17] by comparison of their NMR spectroscopic data with those reported in the literature.

The isolates were evaluated for their α-glucosidase inhibitory and NO production activities. As a result, compound **4** showed significant NO production inhibitory activity, with IC_50_ values of 29.60 μM. Compounds **3** and **4** demonstrated potent α-glucosidase inhibition with IC_50_ values of 44.17 and 60.15 μM, respectively, which was lower than the positive control acarbose (Table 5). Therefore, **3** and **4** might serve as promising bioactive constituents for this plant as a healthy tea for the treatment of diabetes, and could be of interest as possible antidiabetic compounds for further biological evaluation. Overproduction of NO by NOSs could activate proinflammatory mediators associated with acute and chronic inflammations. NO production interference is used for the discovery of new anti-inflammatory agents. The plant *M. hupehensis* has also been documented in folk medicine for its anti-inflammatory uses.

**Figure 3:**
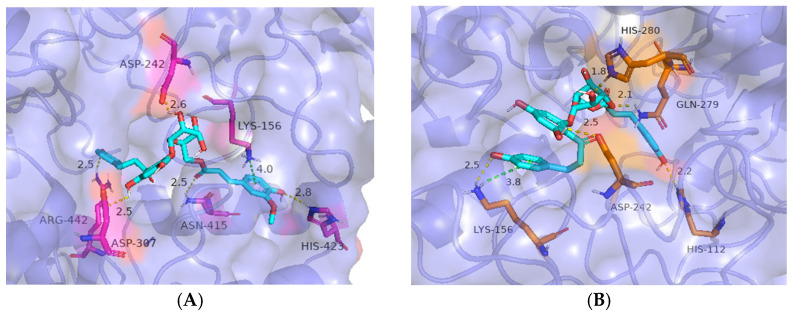
Molecular docking of **3** and **4** and α-glucosidase. The 3D structure of α-glucosidase is shown in blue slate color, ligands are shown in cyan, side chain amino acids are shown in violet, and distances (Angstrom) are shown in black; (**A**) interactions of **3**; (**B**) interactions of **4**.

Binding models of compounds **3** and **4** with α-glucosidase were depicted in Figure 3. It is clear from Figure 3A that **3** displayed two interactions with Lys-156, Asp-242, Lys-156, His-280, and Gln-279 residues. Two H-bonds were formed between the hydroxyl groups of the phloretin molecule and Lys-156 (2.5 Å) and Asp-242 (2.5 Å), in which the B ring made a cation–π interaction with Lys-156 (3.8 Å). The 2″ and 3″-hydroxy of the sugar unit formed two hydrogen bonds with His-280 (1.8 Å) and Gln-279 (2.1 Å), respectively. Similarly, as seen in Figure 3B, **4** displayed two interactions with Arg-442, Asp-307, Asp-242, His-423, Asn-415, and Lys-156 residues. Two H-bonds were formed between the hydroxyl groups of the phloretin molecule and Arg-442 (2.5 Å) and Asp-307 (2.5 Å), in which the B ring made a cation–π interaction with Lys-156 (4.0 Å). The 2″-hydroxy of the sugar unit formed one hydrogen bond with Asp-242 (2.6 Å). The 4′′′-hydroxy formed one hydrogen bond with His-423 (2.8 Å), and the oxygen atom of the carbonyl group formed one hydrogen bond with Asn-415 (2.5 Å). Molecular docking explains the action mechanism between inhibitors and target proteins.

## 3. Materials and Methods

### 3.1. General Experimental Procedures

Sephadex LH-20 and silica gel were used for column chromatography (CC). TLC was carried out using GF254 plates. On a PuriFlash 450 equipment (Interchim, Montluçon, France), medium-pressure liquid chromatography (MPLC) was carried out. Preparative HPLC was performed on a Zorbax SB-C18 column (5 µm, 9.4 × 150 mm) utilizing a DAD detector on an Agilent 1260 liquid chromatography system (Agilent Technologies, Santa Clara, CA, USA). On an Agilent 1260 liquid chromatograph using a Zorbax SB-Aq column (5 µm, 4.6 × 250 mm), semipreparative HPLC was carried out. The Bruker Avance III 600 MHz spectrometer (Bruker, Karlsruhe, Germany) was utilized to obtain NMR spectra. On a Q Exactive Orbitrap mass spectrometer (Thermo Scientific, Waltham, MA, USA), HRESIMS spectra were acquired. Optical rotation was collected on a Rudolph Autopol IV-T polarimeter (Rudolph, Hackettstown, NJ, USA), CD spectra were recorded on a Chirascan Applied Photophysics Chirascan-Plus CD (Applied Photophysics Ltd., London, UK), and the UV spectra were obtained on a Rudolph Autopol IV-T polarimeter (Rudolph, Hackettstown, NJ, USA).

### 3.2. Plant Material

The leaves of *Malus hupehensis* (Pamp.) Rehder (Rosaceae) were collected in Shennongjia Forestry District (110.276105° E, 31.454418° N), Hubei Province of China, in September 2020. The plant material was identified by Prof. Dr. Hua Peng, Kunming Institute of Botany (Kunming, China), and a voucher specimen (No. HB202001) is held at South-Central Minzu University.

### 3.3. Extraction and Isolation

The leaves of *M. hupehensis* (3 kg) were extracted with 95% EtOH at room temperature for 4 weeks, with maceration performed four times. The solvent was evaporated under reduced pressure to yield the EtOH extract, and this extract was suspended in water (1 L) and extracted with EtOAc (5 × 3 L) to give the EtOAc extraction (82.01 g). The EtOAc extraction was separated by MPLC and eluted using a gradient of MeOH/H_2_O (0–100%) to afford five fractions (Frs. A–E). Fr. B was subjected to Sephadex LH20 (MeOH) to produce ten subfractions; Fr. B10 was subjected to silica gel CC (CHCl_3_/MeOH, *v*/*v*, from 100:0 to 0:100) and yielded six subfractions; Fr. B10d was further purified by HPLC using CH_3_CN/H_2_O to obtain compounds **1** (2.6 mg), **11** (4.3 mg), **10** (4.6 mg), and **2** (3.3 mg). Fr. B11 was purified by Sephadex LH-20 (MeOH) to produce four subfractions; Fr. B11c was separated by HPLC to obtain compound **13** (10.8 mg). Compounds **3** (7.6 mg), **4** (4.6 mg), and **12** (3.8 mg) were isolated from subfraction B11b by silica gel CC and HPLC. Fr. C was subjected to silica gel CC (CHCl_3_/MeOH, *v*/*v*, from 100:0 to 0:100) to produce six subfractions; Fr. C3 was subjected to Sephadex LH20 (MeOH) to produce five subfractions; Fr. C3c further purified by HPLC to obtain compounds **9** (6.6 mg) and **15** (3.4 mg). Fr. C4 was subjected to silica gel CC (CHCl_3_/MeOH, *v*/*v*, from 100:0 to 0:100) to provide eight subfractions, and Fr. C4b was further purified by HPLC to obtain compound **8** (27.2 mg). Fr. C4c was subjected to silica gel CC (CHCl_3_/MeOH, *v*/*v*, from 100:0 to 0:100) to produce six subfractions, and Fr. C4c3 and Fr. C4c3 were further purified by HPLC to obtain compounds **5** (5.3 mg), **6** (4.1 mg), **7** (7.9 mg), and **14** (4.0 mg).

Malahupinoside C (**1**): pale-yellow powder; [α]^25^_D_ –8.2 (*c* 0.10, MeOH); UV (MeOH) *λ*_max_ (log *ε*) 210 (3.72), 225 (3.80), and 285 (3.54) nm; ^1^H (600 MHz) and ^13^C NMR (150 MHz) data (CD_3_OD), see Table 1 and Table 2; HRESIMS *m/z* 563.15247 (calcd for C_28_H_28_O_11_Na [M + Na]^+^, 563.15238).

Malahupinoside D (**2**): pale-yellow powder; [α]^25^_D_ –16.1 (*c* 0.13, MeOH); UV (MeOH) *λ*_max_ (log *ε*) 210 (3.85), 220 (3.85), and 280 (3.86) nm; ^1^H (600 MHz) and ^13^C NMR (150 MHz) data (CD_3_OD), see Table 1 and Table 2; HRESIMS *m/z* 589.16821 (calcd for C_30_H_30_O_11_Na [M + Na]^+^, 589.16803).

Malahupinoside E (**3**): pale-yellow powder; [α]^25^_D_ +27.7 (*c* 0.04, MeOH); UV (MeOH) *λ*_max_ (log *ε*) 230 (4.83), 290 (4.86), and 310 (4.88) nm; ^1^H (600 MHz) and ^13^C NMR (150 MHz) data (CD_3_OD), see Table 1 and Table 2; HRESIMS *m/z* 605.16467 (calcd for C_30_H_30_O_12_Na [M + Na]^+^, 605.16295).

Malahupinoside F (**4**): pale-yellow powder; [α]^25^_D_ +27.2 (*c* 0.04, MeOH); UV (MeOH) *λ*_max_ (log *ε*) 225 (4.96), 290 (4.96), and 320 (4.90) nm; ^1^H (600 MHz) and ^13^C NMR (150 MHz) data (CD_3_OD), see Table 1 and Table 2; HRESIMS *m/z* 635.17535 (calcd for C_31_H_32_O_13_Na [M + Na]^+^, 635.17351).

Fortuneanoside M (**5**): pale-yellow gum; [α]^25^_D_ +3.9 (*c* 0.05, MeOH); UV (MeOH) *λ*_max_ (log *ε*) 220 (3.96), 260 (3.42), 280 (3.15), and 315 (3.04) nm; ^1^H (600 MHz) and ^13^C NMR (150 MHz) data (CD_3_OD), see Table 3; HRESIMS *m/z* 245.08082 (calcd for C_14_H_13_O_4_ [M + H]^+^, 245.08084).

Fortuneanoside N (**6**): pale-yellow gum; [α]^25^_D_ +22.2 (*c* 0.05, MeOH); UV (MeOH) *λ*_max_ (log *ε*) 225 (3.76), 260 (3.25), and 320 (2.95) nm; ^1^H (600 MHz) and ^13^C NMR (150 MHz) data (CD_3_OD), see Table 3; HRESIMS *m/z* 423.12857 (calcd for C_20_H_23_O_10_ [M + H]^+^, 423.12857).

Fortuneanoside O (**7**): pale-yellow gum; [α]^25^_D_ +35.5 (*c* 0.07, MeOH); UV (MeOH) *λ*_max_ (log *ε*) 210 (3.78), 255 (3.29), 280 (3.30), and 315 (2.88) nm; ^1^H (600 MHz) and ^13^C NMR (150 MHz) data (CD_3_OD), see Table 3; HRESIMS *m/z* 393.11800 (calcd for C_19_H_21_O_9_ [M + H]^+^, 393.11801).

Fortuneanoside P (**8**): pale-yellow gum; [α]^25^_D_ –26.1 (*c* 0.08, MeOH); UV (MeOH) *λ*_max_ (log *ε*) 210 (4.04), 250 (3.42), and 280 (3.12) nm; ^1^H (600 MHz) and ^13^C NMR (150 MHz) data (CD_3_OD), see Table 4; HRESIMS *m/z* 415.13614 (calcd for C_20_H_24_O_8_Na [M + Na]^+^, 415.13634).

Fortuneanoside Q (**9**): pale-yellow gum; [α]^25^_D_ –4.2 (*c* 0.12, MeOH); UV (MeOH) *λ*_max_ (log *ε*) 210 (4.05) and 280 (3.19) nm; ^1^H (600 MHz) and ^13^C NMR (150 MHz) data (CD_3_OD), see Table 4; HRESIMS *m/z* 431.13116 (calcd for C_20_H_24_O_9_Na [M + Na]^+^, 431.13125).

### 3.4. Determination of the Absolute Configuration of the Sugars

The absolute configuration of the monosaccharide was determined by the previously published method reported by Yuan et al. [18]. Compounds **1**–**4** and **6**–**9** were acid hydrolyzed and derivatized using the methods described. The configurations of D-glucose were determined by comparison of the retention time of the corresponding derivatives with derivatives of the D-glucose standard (t_R_ 17.05 min in this study).

### 3.5. α-Glucosidase Inhibitory Assay

With slight modifications, the α-glucosidase inhibitory activity was determined using the method of Li et al. [19]. In this investigation, *p*-nitrophenol-α-D-glucopyranoside (*p*NPG) was used as the substrate to determine the inhibitor’s inhibitory activity by determining the amount of nitrophenol (PNP) released by enzyme action at a wavelength of 405 nm. A total of 125 µL of 50 mM phosphate buffer (pH 6.8), 25 µL of 10 mM *p*-nitrophenyl-α-D-glucopyranoside (Sigma), and 25 µL of different compounds (final concentrations: 1.25, 2.5, 5, 10, 12.5, 25.00, and 50 µM) were added to a 96-well plate. The mixture was incubated at 37 °C for 10 min. After that, 25 µL of 0.25 U/mL α-glucosidase (Saccharomyces cerevisiae, Sigma) was added, and it was cultured for 30 min at 37 °C. By adding 50 µL of 1 M Na_2_CO_3_, the reaction was stopped. Using an Envision multilabel plate reader, the release of the nitrophenolate group was measured at 405 nm. The triplicates of each experiment were repeated. As a positive control, acarbose was used. The inhibition rates (%) = [(ODcontrol − ODcontrol blank) − (ODtest − ODtest blank)]/(ODcontrol blank − ODcontrol blank) × 100%. The IC_50_ values for the sample were calculated with Graphpad prism 8.

### 3.6. Docking Studies

Molecular docking was performed using Autodock Vina 4.1 to explore the interaction of inhibitors with α-glucosidase. The 3D structure of α-glucosidase (PDB ID: 3A4A) was obtained from the Protein Data Bank (PDB) and further optimized by removing water molecules, hetero atoms, and co-factors. The ligand structures (in pdb format) were generated by ChemDraw Professional 16.0, and the energy was minimized. Autodock Tools 1.5.7 was used to obtain the pdbqt files of both ligand and acceptor. Blind docking was analyzed by Autodock Vina, docking the ligand into the binding pocket of the acceptor, and conformational states were evaluated through cluster analysis in Autodock Tools. The best conformational states were visualized using PyMOL 16.0.

### 3.7. Anti-NO Activity Assay

Using a previously published method [20,21], the anti-inflammatory activity of all compounds (**1**–**15**) was assessed based on the suppression of NO generation in RAW264.7 cells.

## 4. Conclusions

In summary, malahupinosides C-F (**1**–**4**) and fortuneanosides M-Q (**5**–**9**), four new dihydrochalcone glycosides, two new dibenzofuran glycosides, and two new biphenyl glycosides were isolated from *M. hupehensis*. Their structures and absolute configurations of the glycoside were elucidated on the basis of spectroscopic analysis, hydrolyzed acid, and derivatization. It expands our understanding of the bioactive natural products in this herb medicine used in people. Additionally, the α-glucosidase inhibitory and anti-NO activities of compounds **1**–**15** were tested, and results showed that compounds **3**–**4** exhibited α-Glucosidase inhibitory activities, with IC_50_ values of 44.17 and 60.15 μM, respectively, while **4** exhibited NO production inhibitory activity. These findings reveal the active components of the plant that are associated with the role of glucose metabolism in humans, explaining why the plant *M. hupehensis* is useful as a health tea for lowering glucose in humans. The results also provide a basis for the safe and effective application of the plant and the establishment of quality standards.

## Figures and Tables

**Table 1:** ^1^H NMR (600 MHz) spectral data of **1–4** in CD_3_OD.

No.	1	2	3	4
2	6.87, d (8.5)	7.01, d (8.5)	7.07, d (8.3)	7.08, d (8.4)
3	6.58, d (8.5)	6.60, d (8.5)	6.69, d (8.4)	6.69, d (8.4)
5	6.58, d (8.5)	6.60, d (8.5)	6.69, d (8.4)	6.69, d (8.4)
6	6.87, d (8.5)	7.01, d (8.5)	7.07, d (8.3)	7.08, d (8.4)
3′	6.18, d (2.2)	6.17, d (2.2)	6.22, d (2.2)	6.20, d (2.2)
5′	5.92, d (2.2)	5.93, d (2.2)	5.97, d (2.2)	5.95, d (2.2)
1″	5.49, d (8.1)	5.39, d (8.0)	5.08, d (7.4)	5.08, d (7.3)
2″	5.32, dd (9.5, 8.1)	5.20, dd (9.5, 8.2)	3.52, overlapped	3.51, overlapped
3″	3.82, t (8.8)	3.74, overlapped	3.50, overlapped	3.49, overlapped
4″	3.57, overlapped	3.54, overlapped	3.45, t (9.3)	3.45, m
5″	3.59, overlapped	3.55, overlapped	3.72, m	3.72, m
6″	3.95, dd (12.1, 2.0) 3.77, dd (12.1, 5.3)	3.94, dd (12.1, 1.8) 3.76, overlapped	4.55, dd (11.9, 2.2) 4.31, dd (11.9, 6.6)	4.55, dd (11.9, 2.1) 4.33, dd (11.9, 6.4)
2′′′	8.05, dd (8.5, 1.2)	7.58, m	7.39, d (8.6)	7.02, dd (8.1, 1.7)
3′′′	7.47, t (7.8)	7.40, overlapped	6.79, d (8.6)	6.79, d (8.1)
4′′′	7.61, t (7.5)	7.39, overlapped		
5′′′	7.47, t (7.8)	7.40, overlapped	6.79, d (8.6)	
6′′′	8.05, dd (8.5, 1.2)	7.58, m	7.39, d (8.6)	7.10, d (1.7)
8′′′		6.55, d (16.0)	6.34, d (16.0)	6.36, d (15.9)
9′′′		7.72, d (16.0)	7.59, d (16.0)	7.59, d (15.9)
α	3.29, overlapped 3.02, m	3.34, overlapped 3.13, m	3.50, overlapped 3.37, overlapped	3.51, overlapped 3.34, overlapped
β	2.70, m	2.82, m	2.87, m	2.87, m
5′′′-OCH_3_				3.85, d (s)

**Table 2:** ^13^C NMR (150 MHz) spectral data of **1**–**4** in CD3OD.

No.	1	2	3	4
1	133.7, C	133.8, C	133.8, C	133.8, C
2	130.5, CH	130.6, CH	130.4, CH	130.4, CH
3	115.8, CH	115.9, CH	116.1, CH	116.1, CH
4	156.2, C	156.2, C	156.4, C	156.4, C
5	115.8, CH	115.9, CH	116.1, CH	116.1, CH
6	130.5, CH	130.6, CH	130.4, CH	130.4, CH
1′	106.7, C	106.8, C	106.9, C	106.9, C
2′	161.3, C	161.3, C	162.2, C	162.2, C
3′	95.3, CH	95.3, CH	95.9, CH	95.9, CH
4′	165.8, C	165.8, C	166.0, C	165.9, C
5′	98.4, CH	98.4, CH	98.4, CH	98.5, CH
6′	167.1, C	167.2, C	167.5, C	167.6, C
1″	99.2, CH	99.2, CH	102.1, CH	102.1, CH
2″	75.2, CH	74.8, CH	74.7, CH	74.7, CH
3″	76.5, CH	76.5, CH	78.3, CH	78.3, CH
4″	71.3, CH	71.2, CH	71.4, CH	71.3, CH
5″	78.6, CH	78.6, CH	75.7, CH	75.7, CH
6″	62.3, CH_2_	62.3, CH_2_	64.4, CH_2_	64.3, CH_2_
1′′′	131.1, C	135.7, C	127.1, C	127.7, C
2′′′	130.9, CH	129.4, CH	131.3, CH	124.2, CH
3′′′	129.7, CH	130.0, CH	116.7, CH	116.4, CH
4′′′	134.6, CH	131.7, CH	161.2, C	150.6, C
5′′′	129.7, CH	130.0, CH	116.7, CH	149.3, C
6′′′	130.9, CH	129.4, CH	131.3, CH	111.5, CH
7′′′	167.2, C	167.5, C	169.1, C	169.0, C
8′′′		118.5, CH	114.8, CH	115.1, CH
9′′′		147.2, CH	146.9, CH	147.1, CH
α	46.6, CH_2_	46.6, CH_2_	47.0, CH_2_	47.1, CH_2_
β	30.0, CH_2_	30.2, CH_2_	30.9, CH_2_	31.0, CH_2_
C=O	205.8, C	206.0, C	206.5, C	206.6, C
5′′′-OCH_3_				56.4, CH_3_

**Table 3:** ^1^H NMR (600 MHz) and ^13^C NMR (150 MHz) spectral data of **5**–**7** in CD_3_OD.

No.	5		6		7	
1	98.2, CH	6.89, d (2.2)	133.0, C		133.2, C	
2	155.5, C		140.3, C		140.2, C	
3	100.6, CH	6.58, d (2.2)	101.4, CH	6.69, s	101.4, CH	6.67, s
4	147.3, C		144.3, C		144.2, C	
4a	140.9, C		147.1, C		147.0, C	
5a	147.0, C		146.7, C		157.6, C	
6	147.2, C		146.7, C		112.0, CH	7.51, d (8.1)
7	110.7, CH	7.03, dd (7.7, 0.8)	110.7, CH	7.05, dd (7.0, 0.5)	128.1, CH	7.44, m
8	124.3, CH	7.22, t (7.8)	124.2, CH	7.23, t (8.0)	123.5, CH	7.30, m
9	113.7, CH	7.44, dd (7.7, 0.8)	117.5, CH	8.21, dd (7.8, 0.9)	125.5, CH	8.65, d (7.8)
9a	127.3, C		125.9, C		124.4, C	
9b	127.1, C		120.8, C		120.5, C	
1′			107.5, CH	4.83, d (7.8)	107.6, CH	4.84, d (7.9)
2′			75.6, CH	3.64, dd (9.2, 7.9)	75.6, CH	3.65, dd (9.2, 8.0)
3′			78.0, CH	3.46, t (9.2)	78.0, CH	3.47, t (9.2)
4′			71.0, CH	3.53, t (9.2)	70.9, CH	3.54, t (9.2)
5′			78.4, CH	3.26, m	78.4, CH	3.27, m
6′			62.2, CH_2_	3.78, dd (11.8, 2.4) 3.73, dd (11.8, 4.4)	62.2, CH_2_	3.78, dd (11.8, 2.4) 3.74, dd (11.8, 4.4)
4-OCH_3_	56.7, CH_3_	3.99, s	57.0, CH_3_	3.98, s	57.0, CH_3_	3.97, s
6-OCH_3_	56.7, CH_3_	4.01, s	56.7, CH_3_	4.01, s		

**Table 4:** ^1^H NMR (600 MHz) and ^13^C NMR (150 MHz) spectral data of **8**–**9**.

No.	8 ^a^		9 ^b^	
1	132.8, C		125.9, C	
2	155.4, C		154.2, C	
3	116.5, CH	7.26, dd (8.7, 1.2)	115.5, CH	6.85, d (8.0)
4	129.8, CH	7.30, overlapped	128.1, CH	7.10, t (7.6)
5	123.4, CH	7.07, td (7.4, 1.2)	118.3, CH	6.76, t (7.2)
6	131.8, CH	7.31, overlapped	132.0, CH	7.24, d (7.2)
1′	141.8, C		133.5, C	
2′	108.9, CH	6.75, d (2.3)	107.5, CH	6.29, d (2.0)
3′	161.8, C		154.9, C	
4′	100.4, CH	6.43, t (2.3)	99.6, CH	6.59, d (2.0)
5′	161.8, C		152.5, C	
6′	108.9, CH	6.75, d (2.3)	136.5, C	
1″	101.8, CH	5.08, d (8.0)	102.3, CH	4.68, d (7.6)
2″	75.0, CH	3.40, overlapped	74.0, CH	2.90, overlapped
3″	78.4, CH	3.44, overlapped	76.3, CH	3.05, t (8.6)
4″	71.3, CH	3.37, m	69.9, CH	2.93, overlapped
5″	78.2, CH	3.42, overlapped	76.8, CH	2.89, overlapped
6″	62.5, CH_2_	3.87, dd (12.4, 2.2) 3.68, dd (12.4, 5.5)	61.1, CH_2_	3.51, d (11.7) 3.29, overlapped
3′-OCH_3_	55.9, CH_3_	3.80, s	55.3, CH_3_	3.71, s
5′-OCH_3_	55.9, CH_3_	3.80, s	56.2, CH_3_	3.80, s
2-OH				9.05, s

^a^ Measured in CD_3_OD. ^b^ Measured in DMSO-*d*_6_.

**Table 5:** α-Glucosidase inhibitory and NO production activities of compounds **1**–**15** (in IC_50_ μM) ^a^.

Compounds	α-Glucosidase	RAW 264.7
**1**	>1000	>40
**2**	>1000	>40
**3**	44.17	>40
**4**	60.15	29.60
**5**	>1000	>40
**6**	>1000	>40
**7**	>1000	>40
**8**	>1000	>40
**9**	>1000	>40
**10**	>1000	>40
**11**	>1000	>40
**12**	>1000	>40
**13**	>1000	>40
**14**	>1000	>40
**15**	>1000	>40
Ammonium pyrrolidinedithiocarbamate ^b^		20 ± 0.12
Acarbose ^b^	1000 ± 1.22	

^a^ IC_50_ (μM) represents means of three independent replicates. ^b^ Positive control.

## Data Availability

Data are contained within the article and Supplementary Materials.

## Supplementary Materials

The following supporting information can be downloaded at https://www.mdpi.com/article/10.3390/molecules29225269/s1. Figures S1–S63: The HRESIMS and NMR spectrum of compounds **1**–**9**. Figure S64: The reversed-phase HPLC spectra of derivatives.

## References

[1] Janssen S.W.J., Martens G.J.M., Sweep C.G.J., Span P.N., Verhofstad A.A.J., Hermus A.R.M.M. (2003). Phlorizin treatment prevents the decrease in plasma insulin levels but not the progressive histopathological changes in the pancreatic islets during aging of Zucker diabetic fatty rats. J. Endocrinol. Investig..

[2] Xiao Z.C., Zhang Y.Y., Chen X., Wang Y.L., Chen W.F., Xu Q.P., Li P.M., Ma F.W. (2017). Extraction, identification, and antioxidant and anticancer tests of seven dihydrochalcones from *Malus* ‘Red Splendor’ fruit. Food. Chem..

[3] Hu Q.W., Chen Y.Y., Jiao Q.Y., Khan A., Shan J., Cao G.D., Li F., Zhang C., Lou H.X. (2017). Polyphenolic compounds from *Malus hupehensis* and their free radical scavenging effects. Nat. Prod. Res..

[4] Cui L.L., Xing M.M., Xu L.T., Wang J.Y., Zhang X.F., Ma C.Y., Kang W.Y. (2018). Antithrombotic components of *Malus halliana* Koehne flowers. Food. Chem. Toxicol..

[5] Qin X.X., Xing Y., Zhou Z.Z., Yao Y.C. (2015). Dihydrochalcone compounds isolated from crabapple leaves showed anticancer effects on human cancer cell lines. Molecules.

[6] Wang S.Q., Zhu X.F., Wang X.N., Shen T., Xiang F., Lou H.X. (2013). Flavonoids from *Malus hupehensis* and their cardioprotective effects against doxorubicin-induced toxicity in H9c2 cells. Phytochemistry.

[7] Liu L.J., Guo D.Y., Fan Y., Sun J., Cheng J.X., Shi Y.J. (2019). Experimental study on the antioxidant activity of *Malus hupehensis* (Pamp.) Rehd extracts in vitro and in vivo. J. Cell. Biochem..

[8] Li M.X., Xue S.J., Tan S., Qin X.X., Gu M., Wang D.S., Zhang Y., Guo L., Huang F.S., Yao Y.C. (2016). Crabapple fruit extracts lower hypercholesterolaemia in high-fat diet-induced obese mice. J. Funct. Foods..

[9] Sun H., Saeedi P., Karuranga S., Pinkepank M., Ogurtsova K., Duncan B.B., Stein C., Basit A., Chan J.C.N., Mbanya J.C. (2022). IDF diabetes Atlas: Global, regional and country-level diabetes prevalence estimates for 2021 and projections for 2045. Diabetes Res. Clin. Pract..

[10] Hossain U., Das A.K., Ghosh S., Sil P.C. (2020). An overview on the role of bioactive α-glucosidase inhibitors in ameliorating diabetic complications. Food Chem. Toxicol..

[11] Patil P., Mandal S., Tomar S.K., Anand S. (2015). Food protein-derived bioactive peptides in management of type 2 diabetes. Eur. J. Nutr..

[12] Hufford C.D., Oguntimein B.O. (1980). Dihydrochalcones from *Uvaria angolensis*. Phytochemistry.

[13] Cheng Z.B., Xu W., Wang Y.Y., Bai S.Y., Liu L.J., Luo Z.H., Yuan W.J., Li Q. (2019). Two new meroterpenoids and two new monoterpenoids from the deep sea-derived fungus *Penicillium* sp. YPGA. Fitoterapia.

[14] Khalil M.N.A., Beuerle T., Müller A., Ernst L., Beerhues L. (2013). Biosynthesis of the biphenyl phytoalexin aucuparin in *Sorbus aucuparia* cell cultures treated with *Venturia inaequalis*. Phytochemistry.

[15] Dai Y., Zhou G.X., Kurihara H., Ye W.C., Yao X.S. (2006). Biphenyl glycosides from the fruit of *Pyracantha fortuneana*. J. Nat. Prod..

[16] Jeong G.H., Cho J.H., Kim S.H., Kim T.H. (2017). Plasma-induced dimerization of phloridzin as a new class of anti-adipogenic agents. Bioorg. Med. Chem. Lett..

[17] Lee I.S., Jung S.H., Lee Y.M., Choi S., Sun H., Kim J.S. (2015). Phenolic compounds from the leaves and twigs of *Osteomeles schwerinae* that inhibit rat lens aldose reductase and vessel dilation in zebrafish larvae. J. Nat. Prod..

[18] Yuan L.L., Shi B.B., Feng T., Huang R., Li Z.H., Chen H.P., Liu J.K. (2022). α-Glucosidase inhibitory phenylpropanoid-dihydrochalcone hybrids from the leaves of medicinal plant *Malus hupehensis* (Pamp.) Rehder. Phytochemistry.

[19] Li W., Wei K., Fu H.W., Koike K. (2007). Structure and absolute configuration of clerodane diterpene glycosides and a rearranged cadinane sesquiterpene glycoside from the stems of *Tinospora sinensis*. J. Nat. Prod..

[20] Green L.C., Wagner D.A., Glogowski J., Skipper P.L., Wishnok J.S., Tannenbaum S.R. (1982). Analysis of nitrate, nitrite, and [15N] nitrate in biological fluids. Anal. Biochem..

[21] Tang Y., Zhao Z.Z., Yao J.N., Feng T., Li Z.H., Chen H.P., Liu J.K. (2018). Irpeksins A−E, 1,10-seco-eburicane-type triterpenoids from the medicinal fungus *Irpex iacteus* and their anti-NO activity. J. Nat. Prod..

