# Supplementary material for: Nine New Glycosylated Compounds from the Leaves of the Medicinal Plant Malus hupehensis
Source: Molecules. 2024 Nov 7;29(22):5269. doi: 10.3390/molecules29225269 (PMC11596612; doi:10.3390/molecules29225269)
Supplement: Supplementary file 1 [file molecules-29-05269-s001.zip › molecules-3296910-supplementary.pdf]

SUPPLEMENTARY MATERIAL

Nine new glycosylated compounds from the leaves of the medicinal plant *Malus hupehensis*

Lin-Lin Yuan[1], Yi Wang[2], Guo-Kai Wang[1,*] and Ji-Kai Liu[1,*]

[1] Anhui Province Key Laboratory of Bioactive Natural Products, School of Pharmaceutical Sciences, Anhui University of Chinese Medicine, Hefei 230012, People's Republic of China

[2] Genpact, 1155 Avenue of the Americas 4th Fl, New York, NY 10036, USA

Emails: wanggk@ahtcm.edu.cn (G.-K. Wang), liujikai@mail.scuec.edu.cn (J.-K. Liu)

Contents

Figure S1. HRESIMS spectroscopic data of compound 1. 3
Figure S2. ^{1}H NMR (600 MHz) spectrum of compound 1. 4
Figure S3. ^{13}C NMR and DEPT (150 MHz) spectra of compound 1. 4
Figure S4. ^{1}H-^{1}H COSY spectrum of compound 1. 5
Figure S5. HSQC spectrum of compound 1. 5
Figure S6. HMBC spectrum of compound 1. 6
Figure S7. ROESY spectrum of compound 1. 6
Figure S8. HRESIMS spectroscopic data of compound 2. 7
Figure S9. ^{1}H NMR (600 MHz) spectrum of compound 2. 7
Figure S10. ^{13}C NMR and DEPT (150 MHz) spectra of compound 2. 8
Figure S11. ^{1}H-^{1}H COSY spectrum of compound 2. 8
Figure S12. HSQC spectrum of compound 2. 9
Figure S13. HMBC spectrum of compound 2. 9
Figure S14. ROESY spectrum of compound 2. 10
Figure S15. HRESIMS spectroscopic data of compound 3. 11
Figure S16. ^{1}H NMR (600 MHz) spectrum of compound 3. 12
Figure S17. ^{13}C NMR and DEPT (150 MHz) spectra of compound 3. 12
Figure S18. ^{1}H-^{1}H COSY spectrum of compound 3. 13
Figure S19. HSQC spectrum of compound 3. 13
Figure S20. HMBC spectrum of compound 3. 14
Figure S21. ROESY spectrum of compound 3. 14
Figure S22. HRESIMS spectroscopic data of compound 4. 15
Figure S23. ^{1}H NMR (600 MHz) spectrum of compound 4. 16
Figure S24. ^{13}C NMR and DEPT (150 MHz) spectra of compound 4. 16
Figure S25. ^{1}H-^{1}H COSY spectrum of compound 4. 17
Figure S26. HSQC spectrum of compound 4. 17
Figure S27. HMBC spectrum of compound 4. 18
Figure S28. ROESY spectrum of compound 4. 18
Figure S29. HRESIMS spectroscopic data of compound 5. 19
Figure S30. ^{1}H NMR (600 MHz) spectrum of compound 5. 20
Figure S31. ^{13}C NMR and DEPT (150 MHz) spectra of compound 5. 20
Figure S32. ^{1}H-^{1}H COSY spectrum of compound 5. 21
Figure S33. HSQC spectrum of compound 5. 21
Figure S34. HMBC spectrum of compound 5. 22
Figure S35. ROESY spectrum of compound 5. 22
Figure S36. HRESIMS spectroscopic data of compound 6. 23
Figure S37. ^{1}H NMR (600 MHz) spectrum of compound 6. 23
Figure S38. ^{13}C NMR and DEPT (150 MHz) spectra of compound 6. 24
Figure S39. ^{1}H-^{1}H COSY spectrum of compound 6. 24
Figure S40. HSQC spectrum of compound 6. 25
Figure S41. HMBC spectrum of compound 6. 25
Figure S42. ROESY spectrum of compound 6. 26

Figure S43. HRESIMS spectroscopic data of compound 7. 27
Figure S44. ^{1}H NMR (600 MHz) spectrum of compound 7. 28
Figure S45. ^{13}C NMR and DEPT (150 MHz) spectra of compound 7. 28
Figure S46. ^{1}H-^{1}H COSY spectrum of compound 7. 29
Figure S47. HSQC spectrum of compound 7. 29
Figure S48. HMBC spectrum of compound 7. 30
Figure S49. ROESY spectrum of compound 7. 30
Figure S50. HRESIMS spectroscopic data of compound 8. 31
Figure S51. ^{1}H NMR (600 MHz) spectrum of compound 8. 32
Figure S52. ^{13}C NMR and DEPT (150 MHz) spectra of compound 8. 32
Figure S53. ^{1}H-^{1}H COSY spectrum of compound 8. 33
Figure S54. HSQC spectrum of compound 8. 33
Figure S55. HMBC spectrum of compound 8. 34
Figure S56. ROESY spectrum of compound 8. 34
Figure S57. HRESIMS spectroscopic data of compound 9. 35
Figure S58. ^{1}H NMR (600 MHz) spectrum of compound 9. 36
Figure S59. ^{13}C NMR and DEPT (150 MHz) spectra of compound 9. 36
Figure S60. ^{1}H-^{1}H COSY spectrum of compound 9. 37
Figure S61. HSQC spectrum of compound 9. 37
Figure S62. HMBC spectrum of compound 9. 38
Figure S63. ROESY spectrum of compound 9. 38
Figure S64. The reversed-phase HPLC spectra of derivatives. 39

Figure S1. HRESIMS spectroscopic data of compound 1.

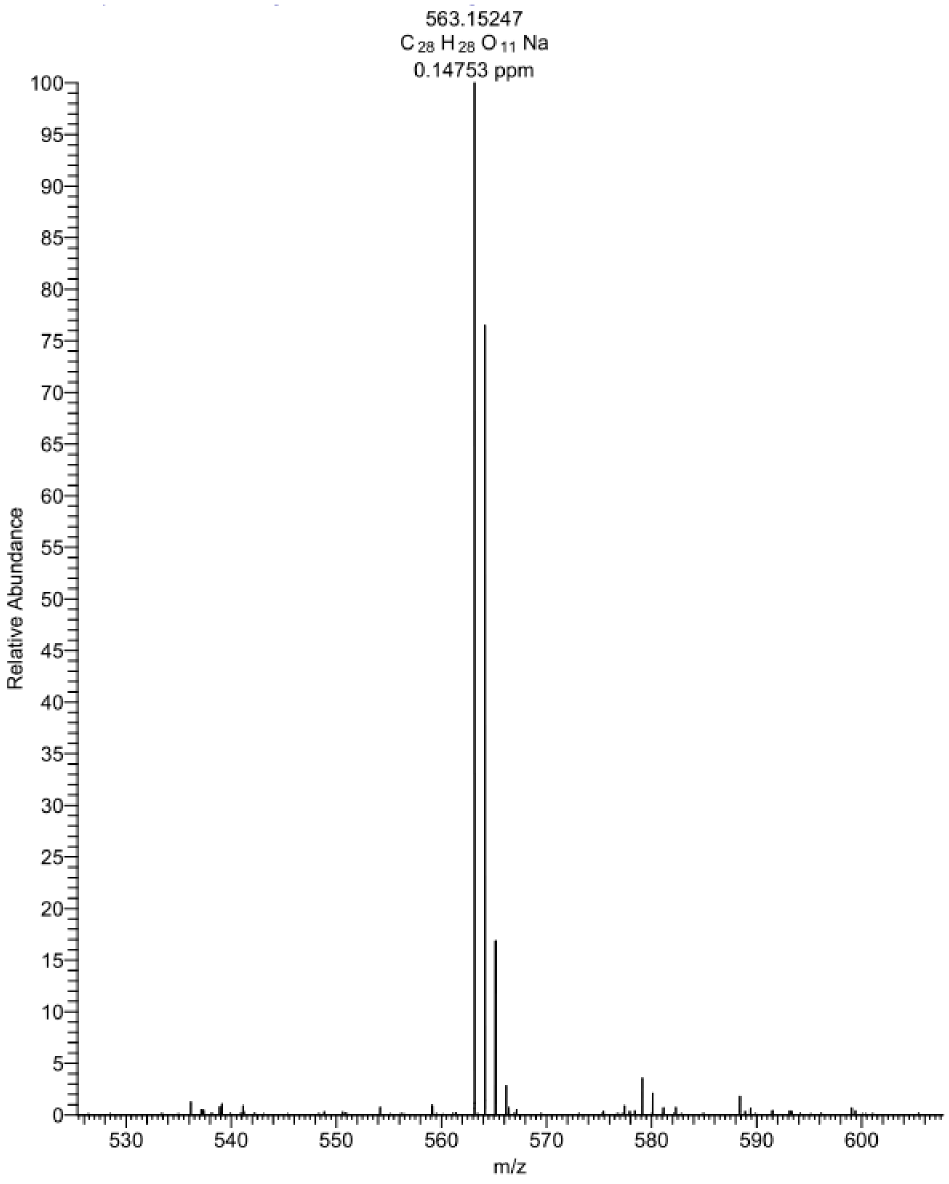

Figure S2. ^{1}**H NMR (600 MHz) spectrum of compound 1.**

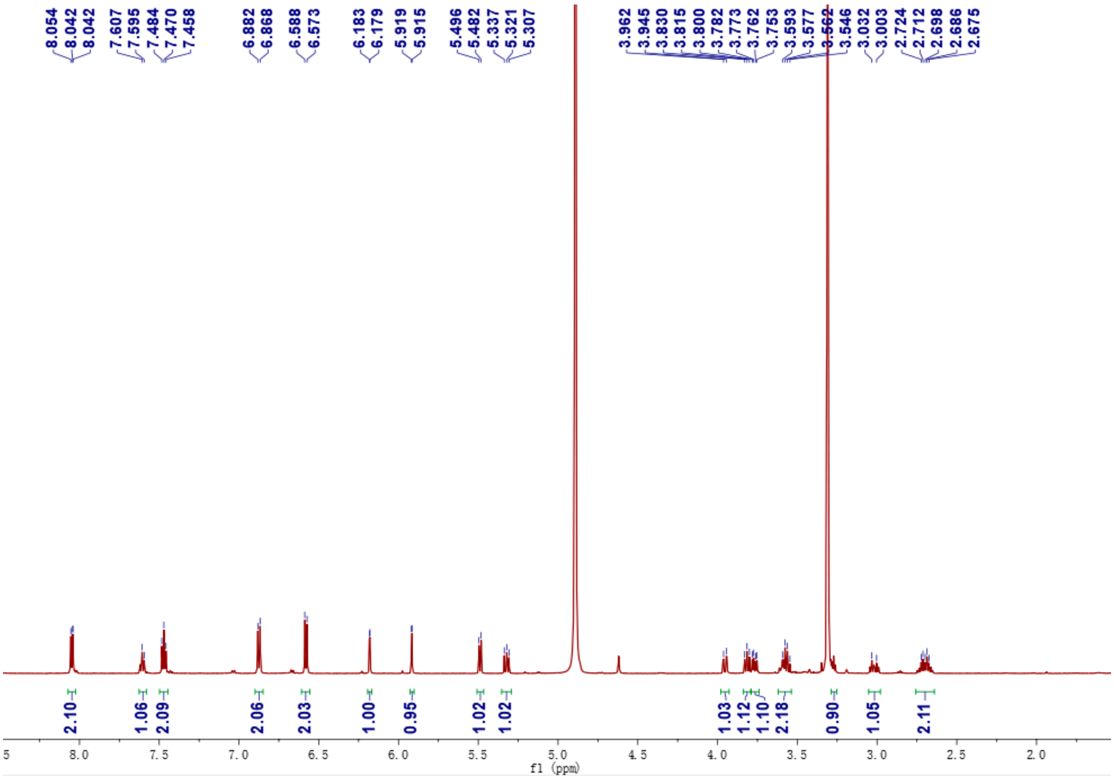

Figure S3. ^{13}**C NMR and DEPT (150 MHz) spectra of compound 1.**

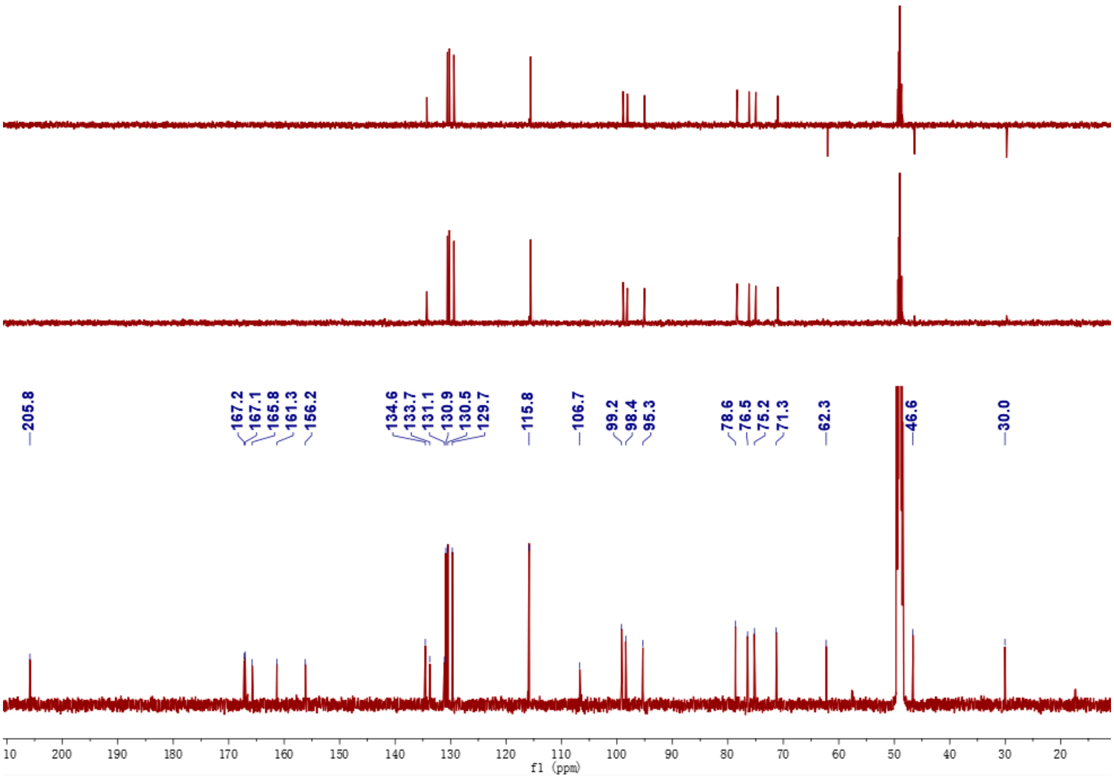

Figure S4. ^{1}H-^{1}H COSY spectrum of compound 1.

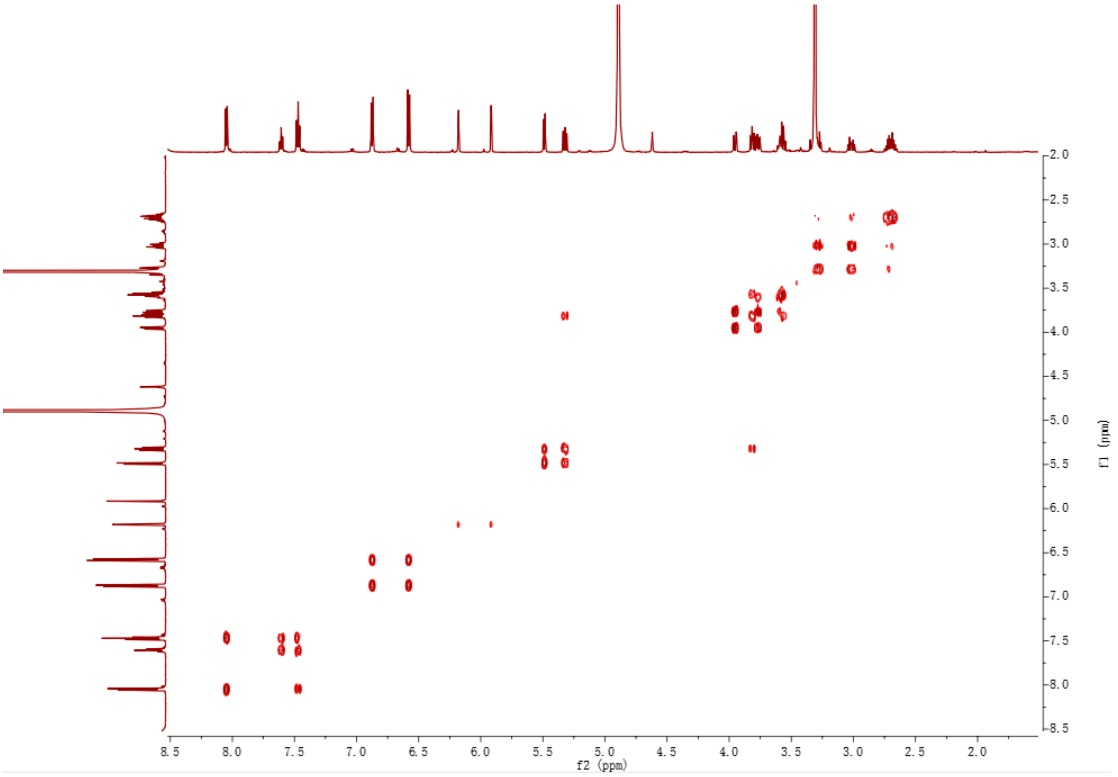

Figure S5. HSQC spectrum of compound 1.

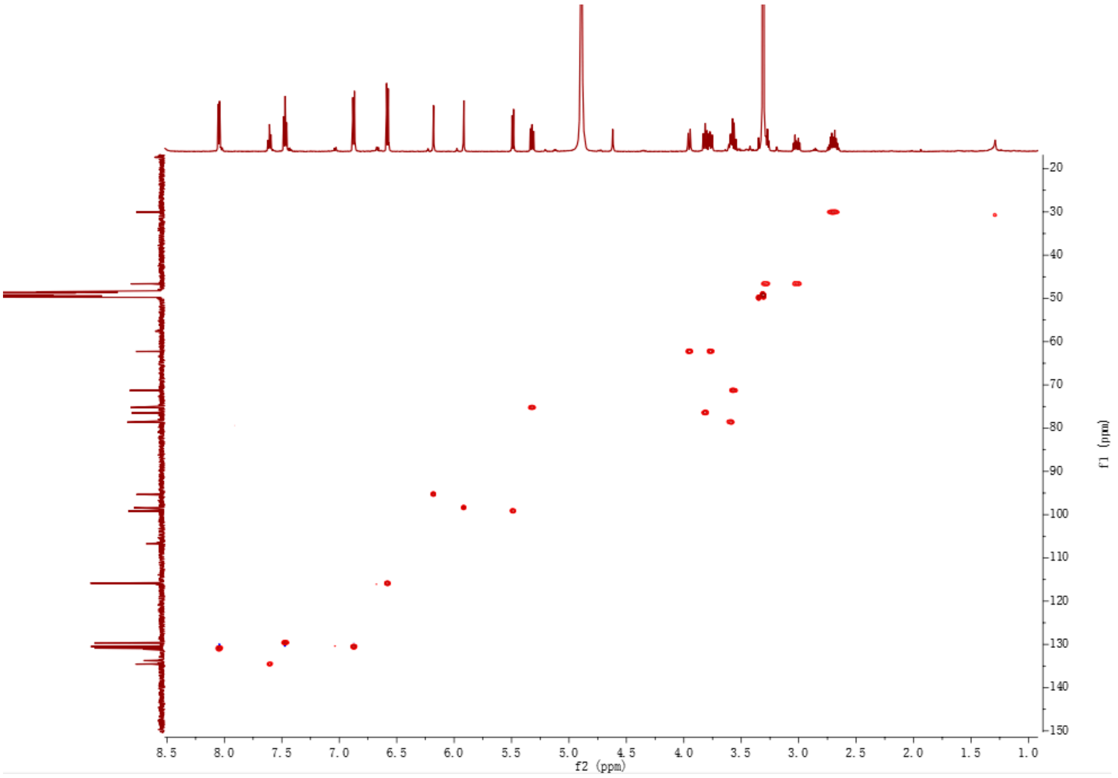

Figure S6. HMBC spectrum of compound 1.

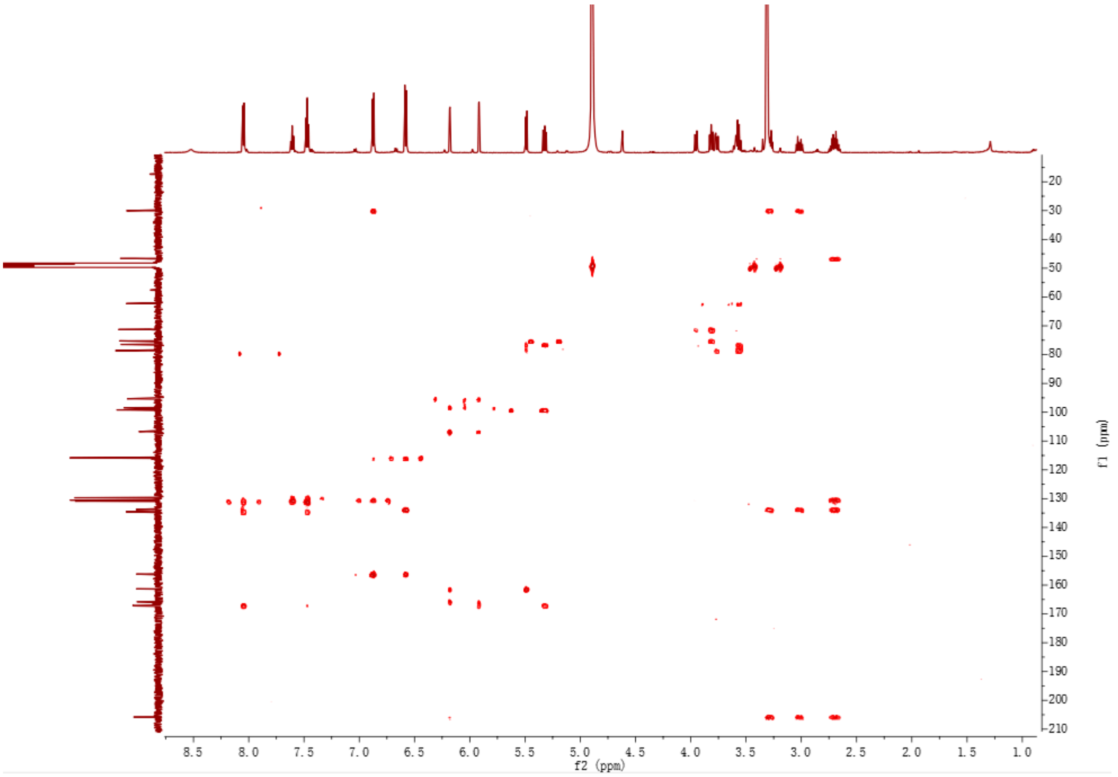

Figure S7. ROESY spectrum of compound 1.

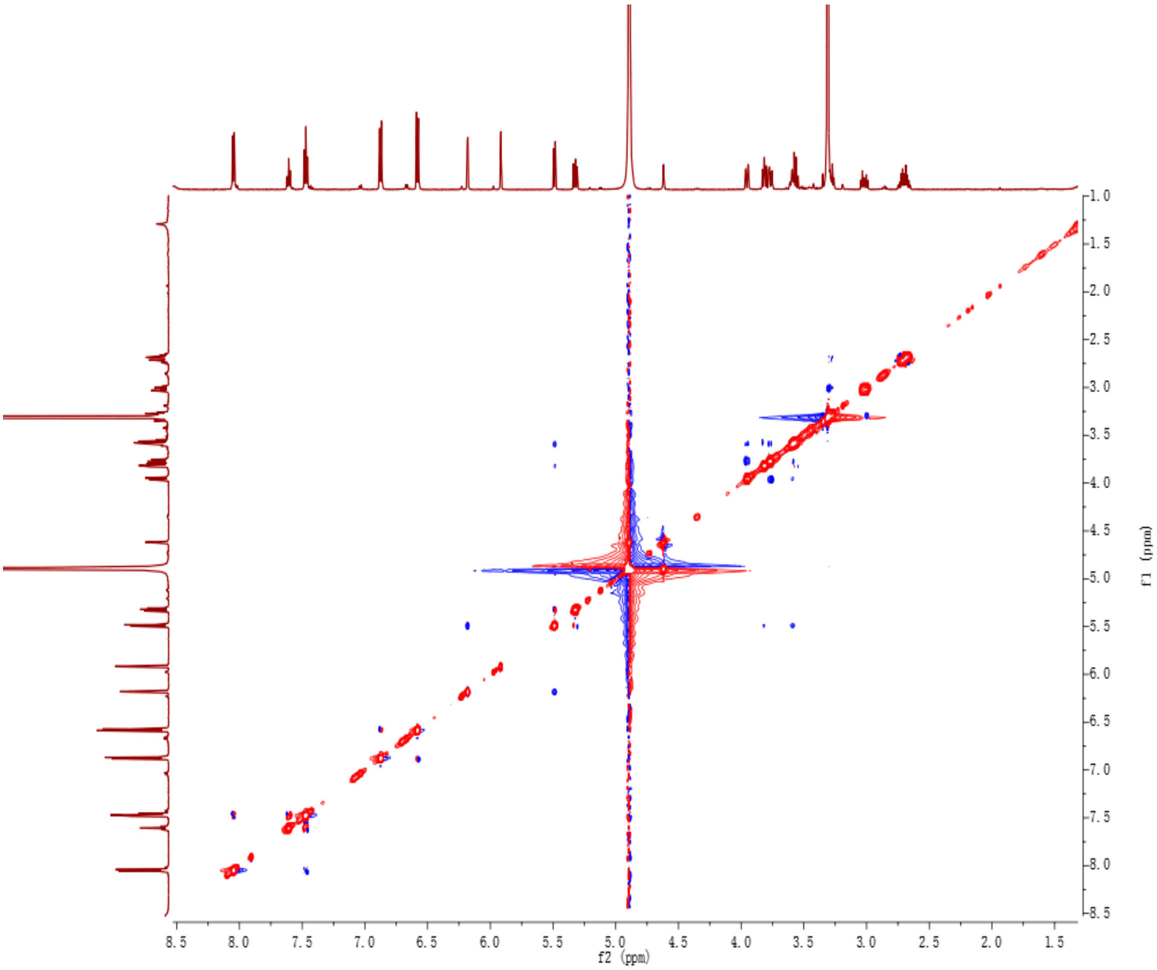

Figure S8. HRESIMS spectroscopic data of compound 2.

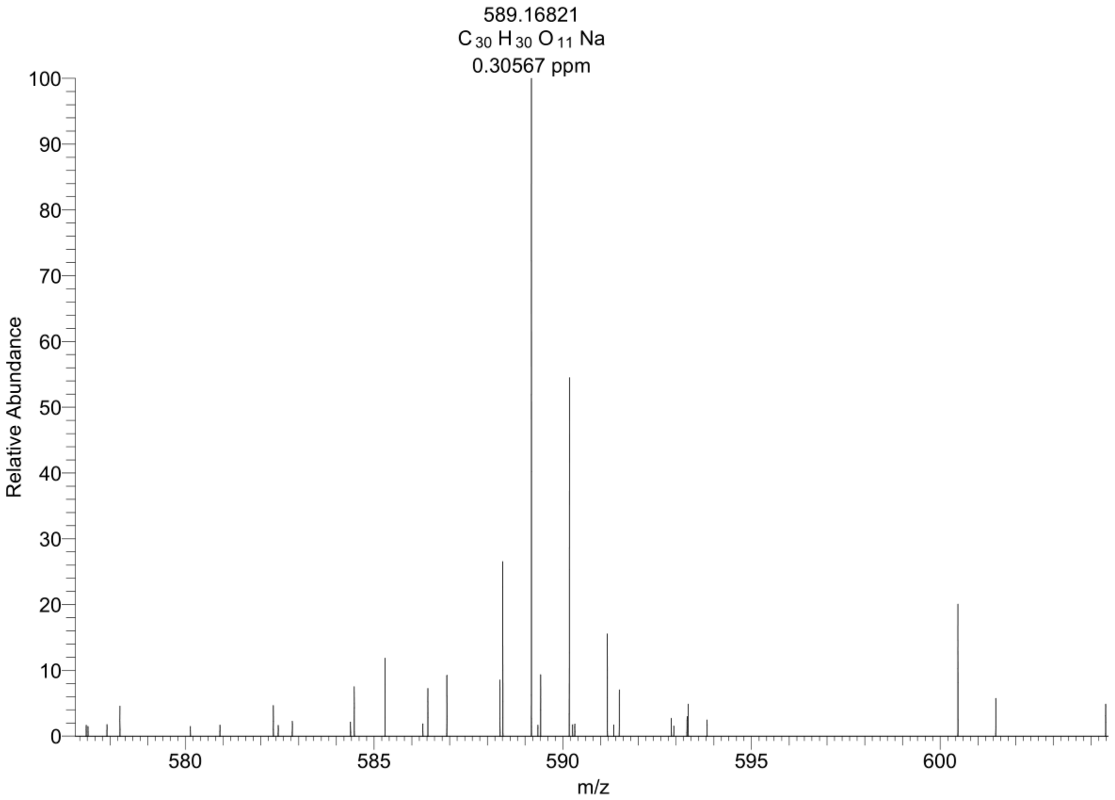

Figure S9. ^{1}H NMR (600 MHz) spectrum of compound 2.

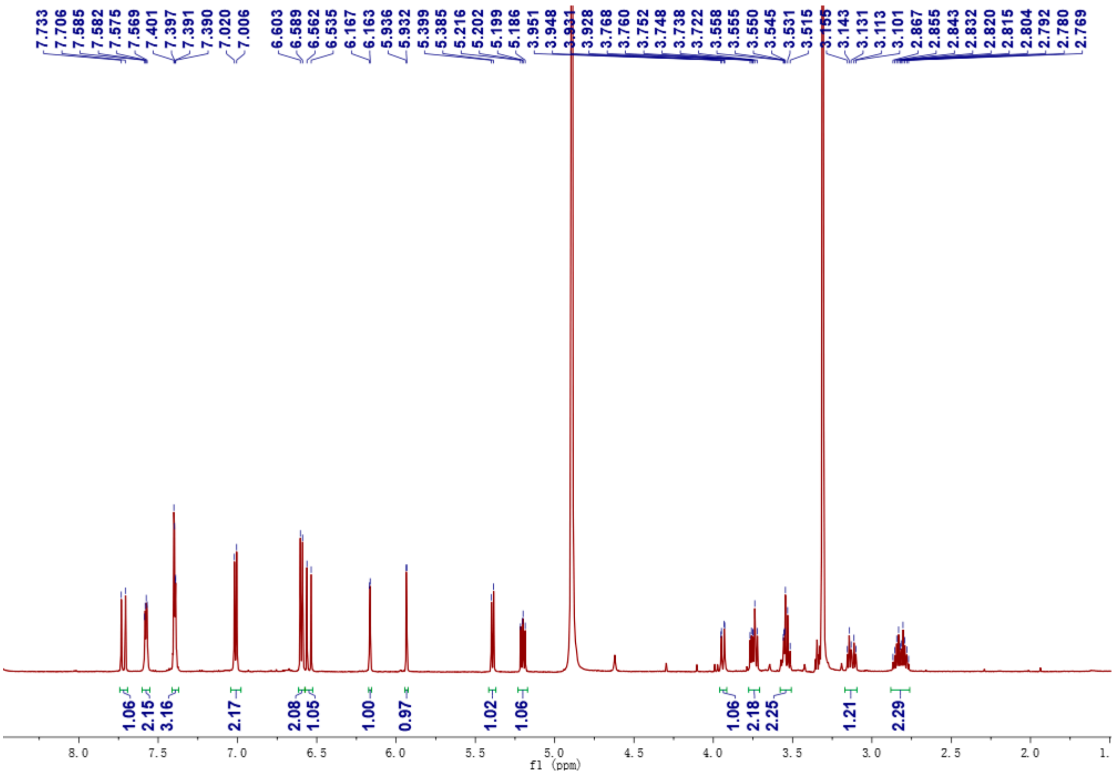

Figure S10. ^{13}C NMR and DEPT (150 MHz) spectra of compound 2.

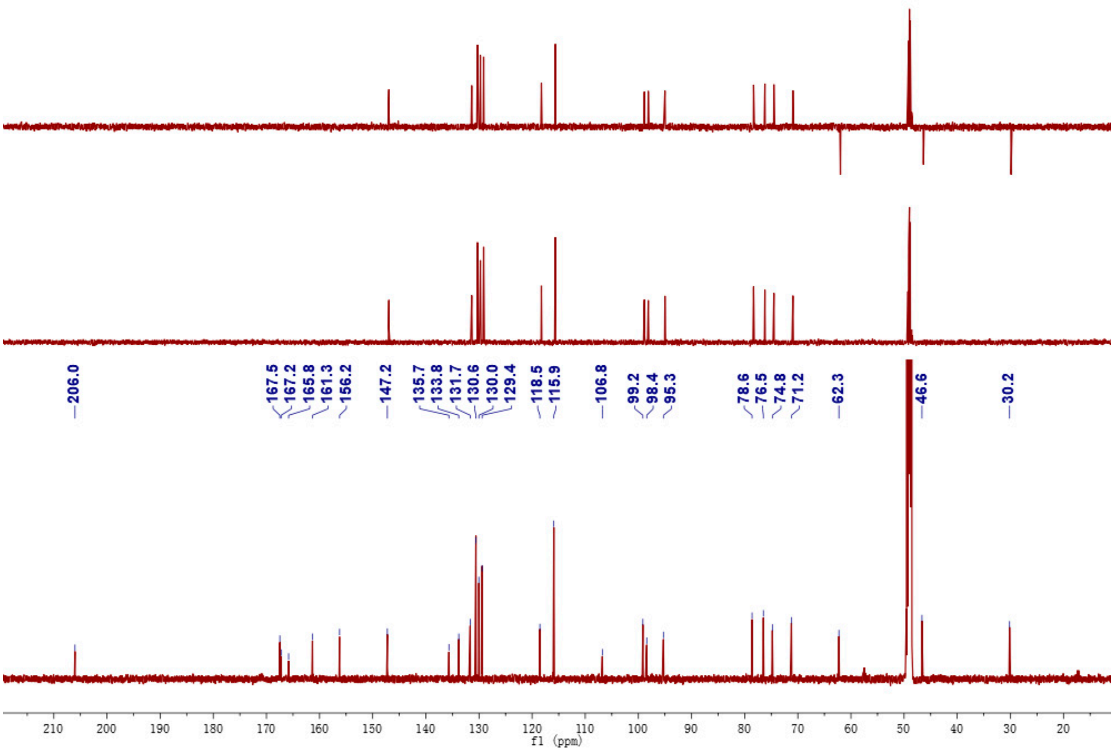

Figure S11. ^{1}H-^{1}H COSY spectrum of compound 2.

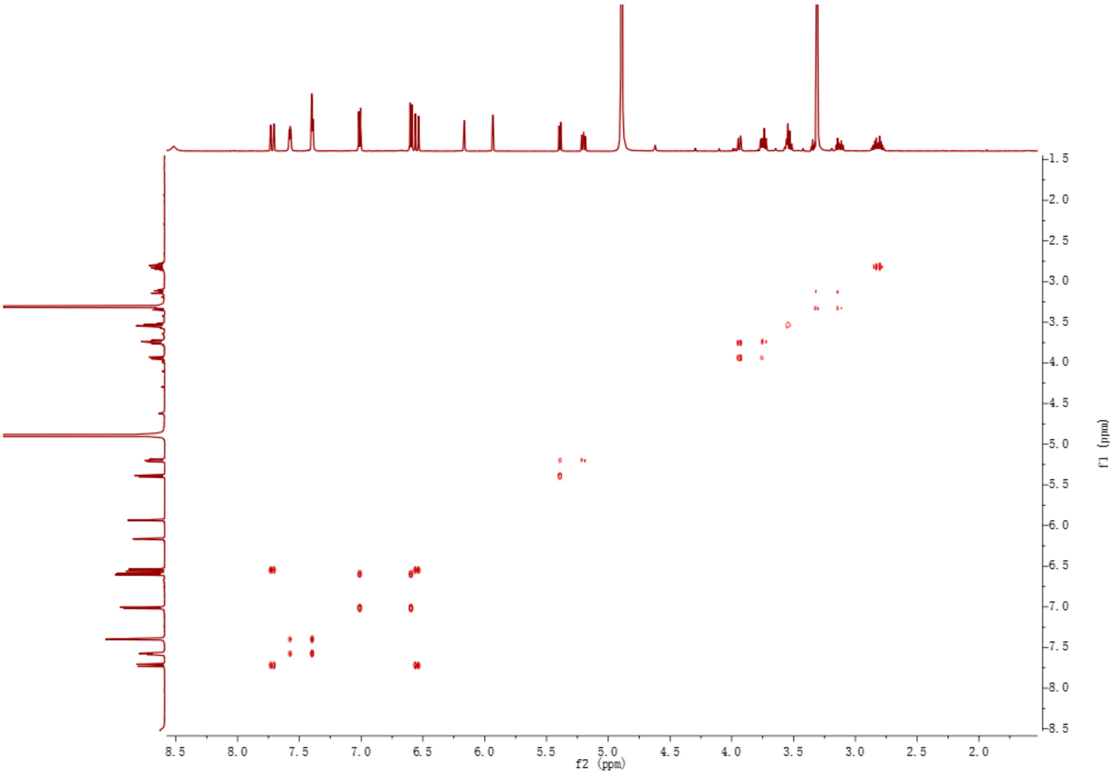

Figure S12. HSQC spectrum of compound 2.

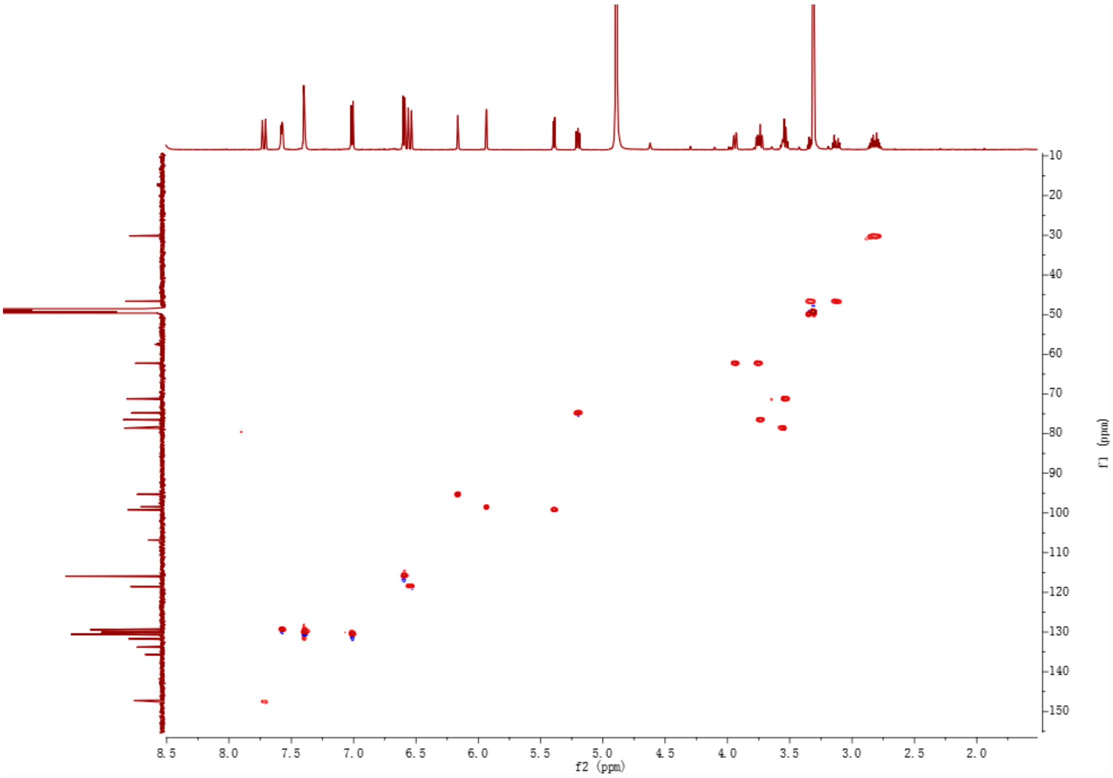

Figure S13. HMBC spectrum of compound 2.

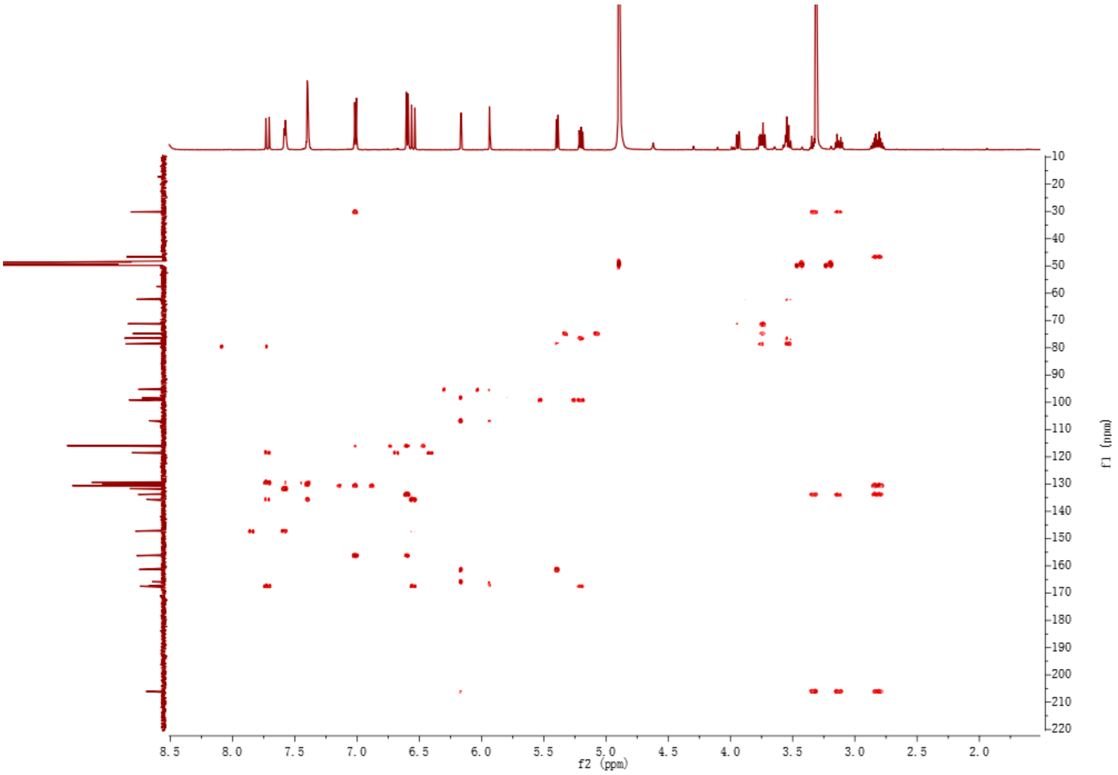

Figure S14. ROESY spectrum of compound 2.

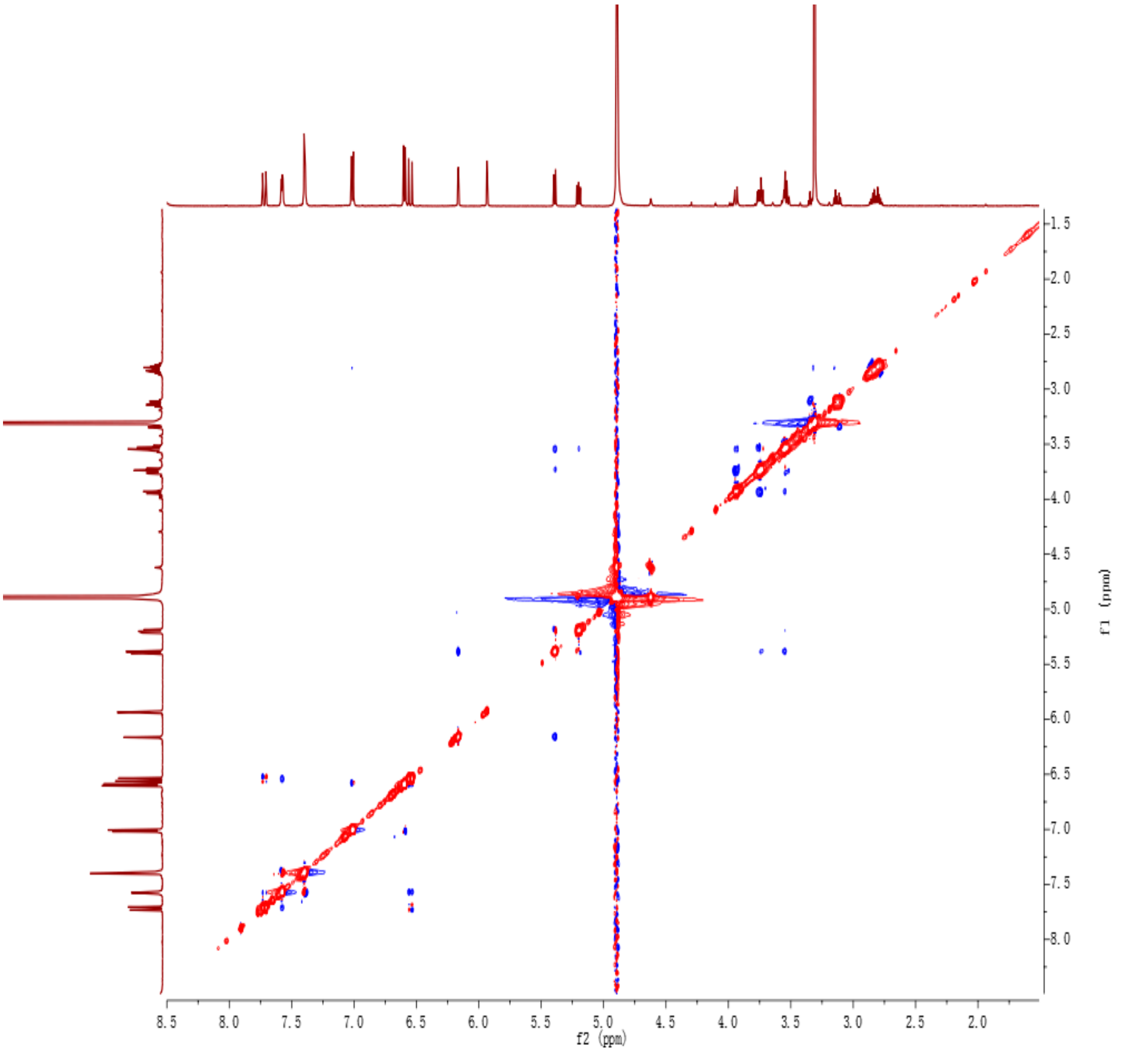

Figure S15. HRESIMS spectroscopic data of compound 3.

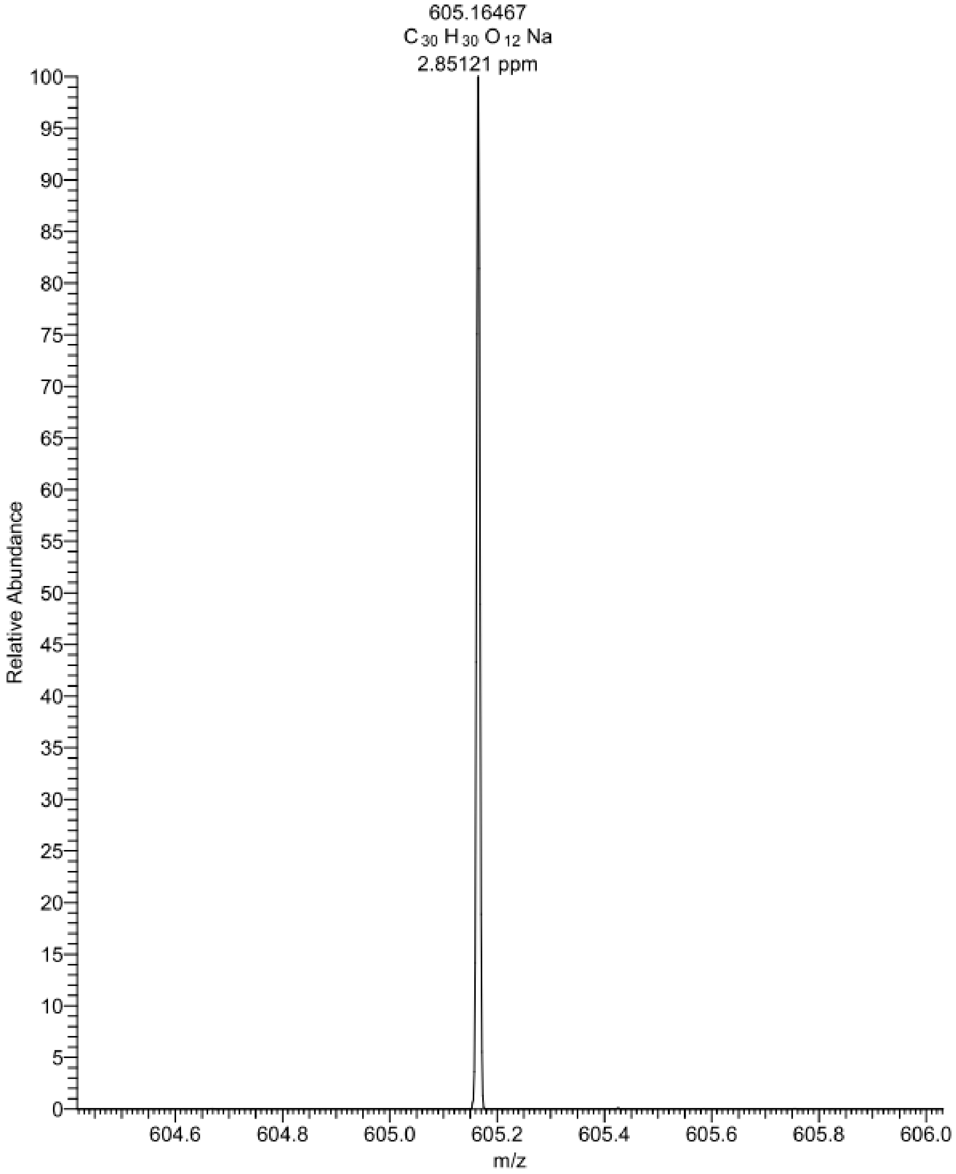

Figure S16. ^{1}H NMR (600 MHz) spectrum of compound 3.

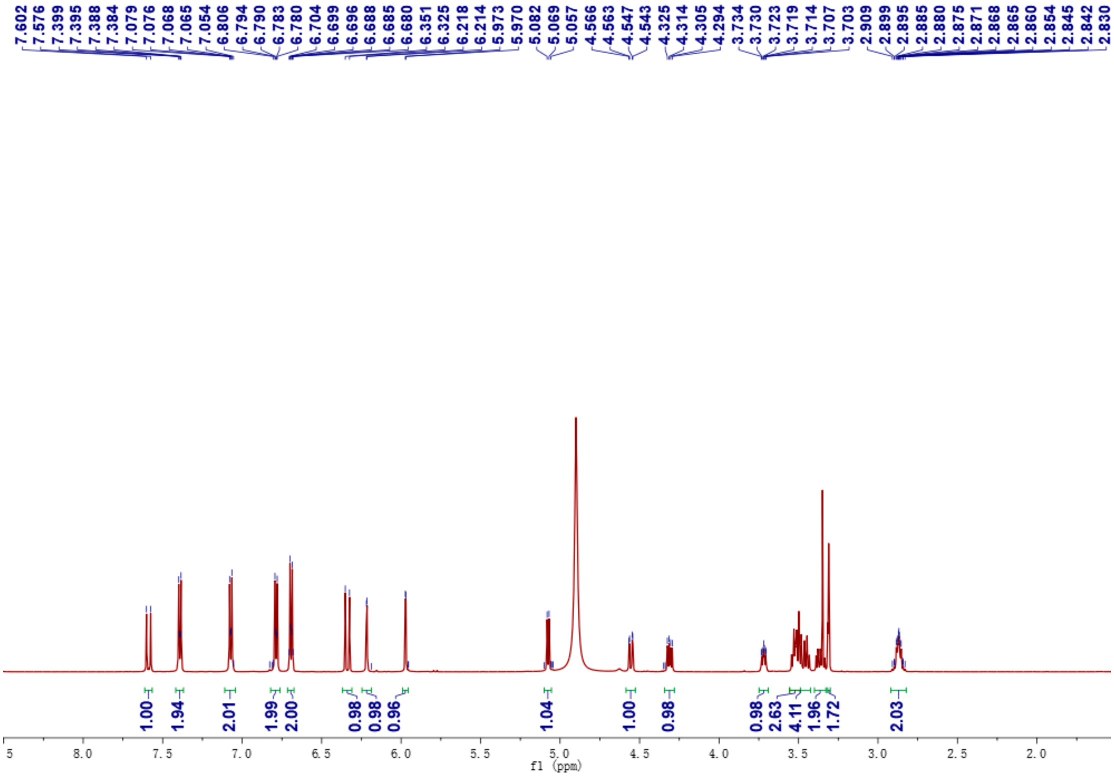

Figure S17. ^{13}C NMR and DEPT (150 MHz) spectra of compound 3.

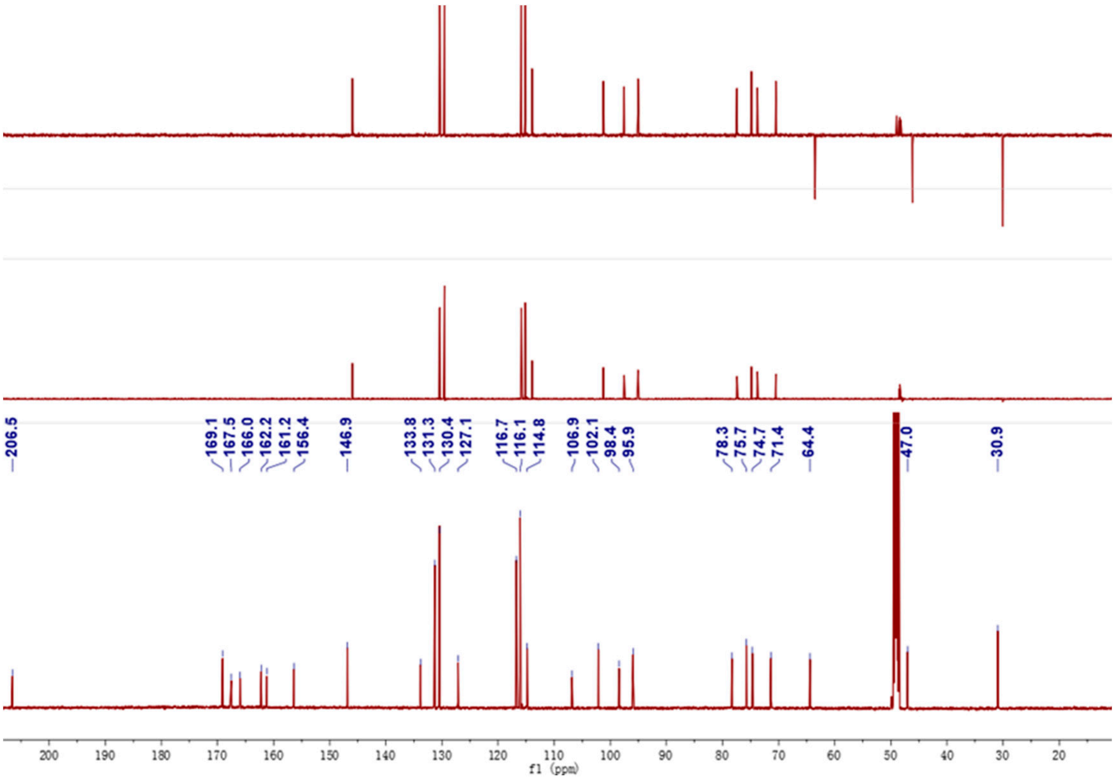

Figure S18. ¹H-¹H COSY spectrum of compound 3.

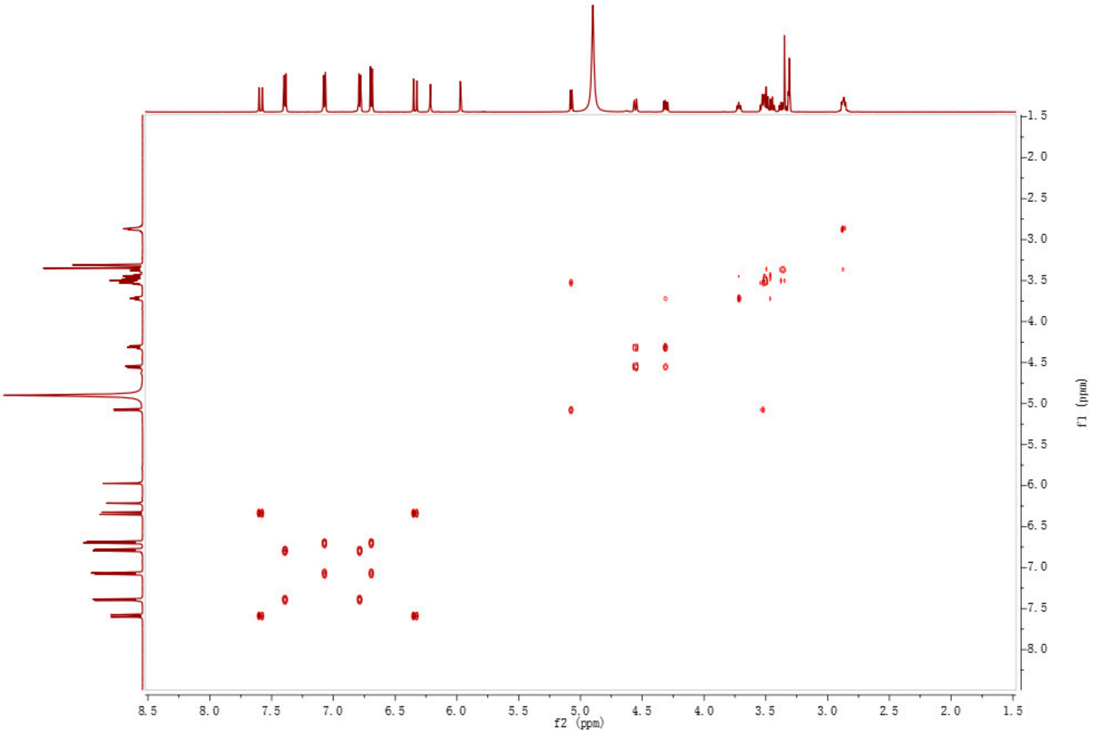

Figure S19. HSQC spectrum of compound 3.

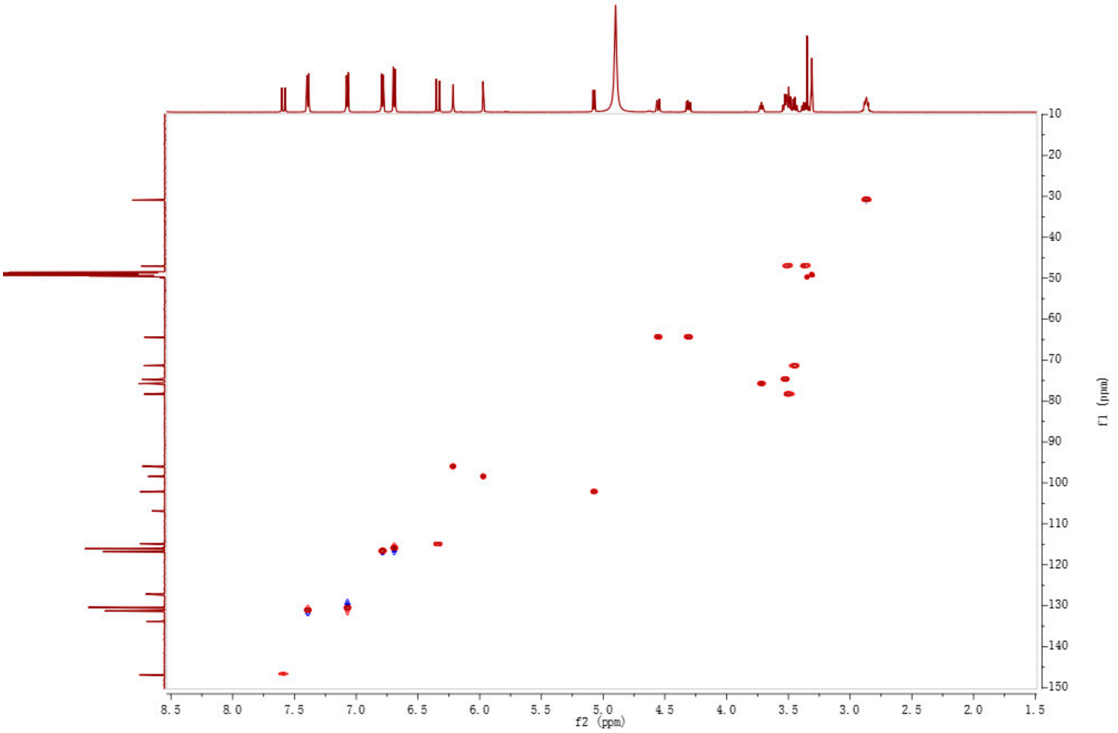

Figure S20. HMBC spectrum of compound 3.

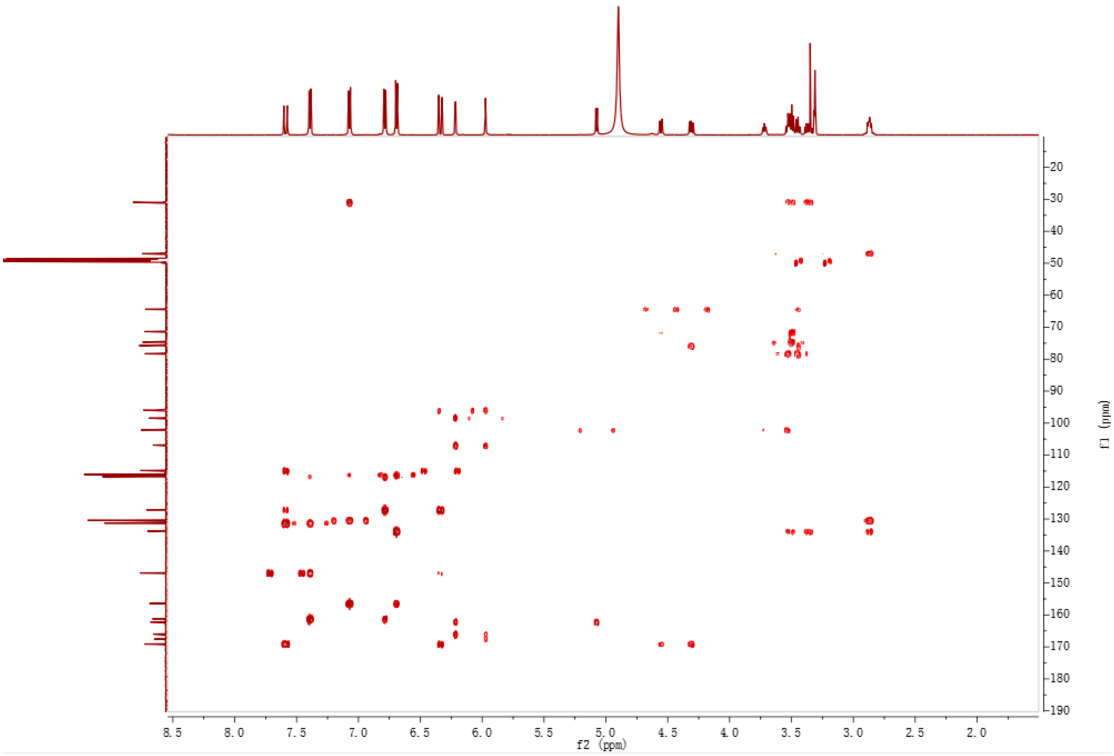

Figure S21. ROESY spectrum of compound 3.

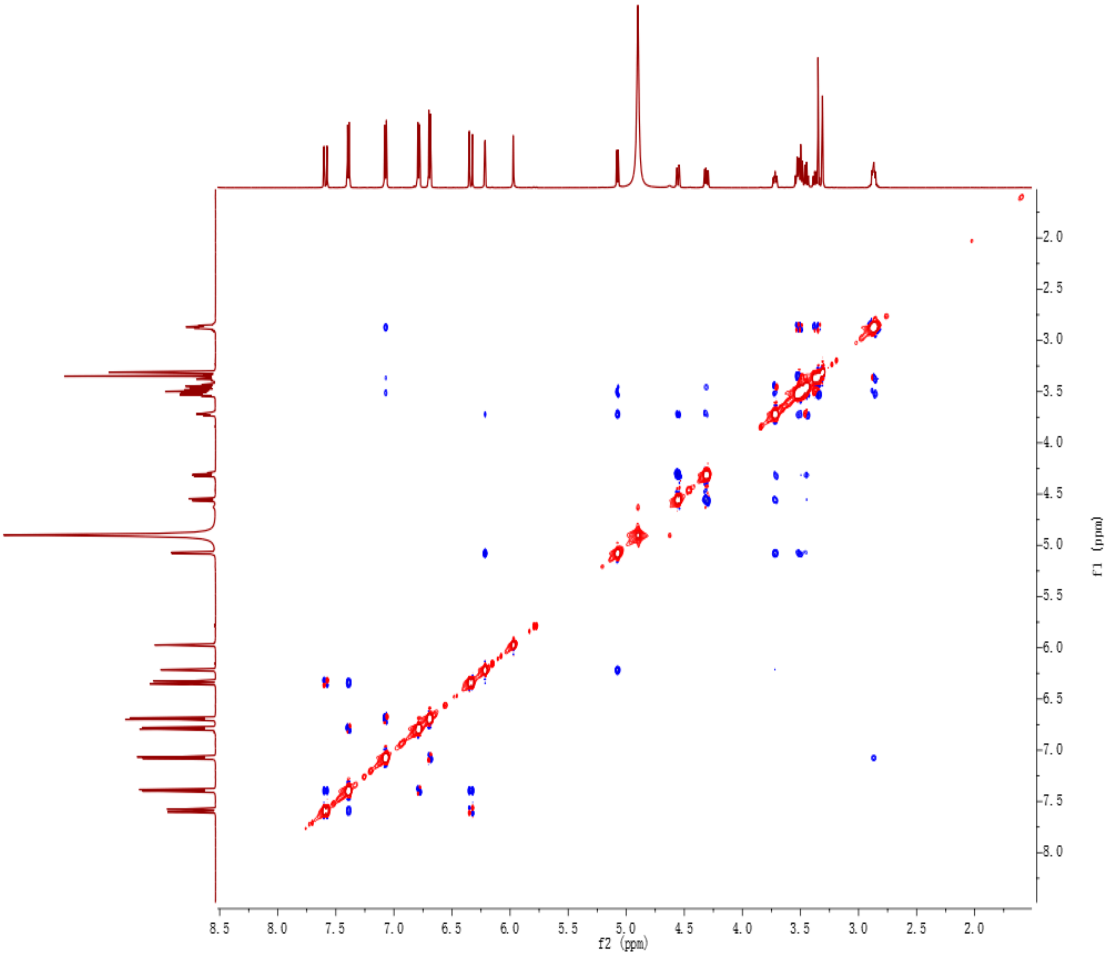

Figure S22. HRESIMS spectroscopic data of compound 4.

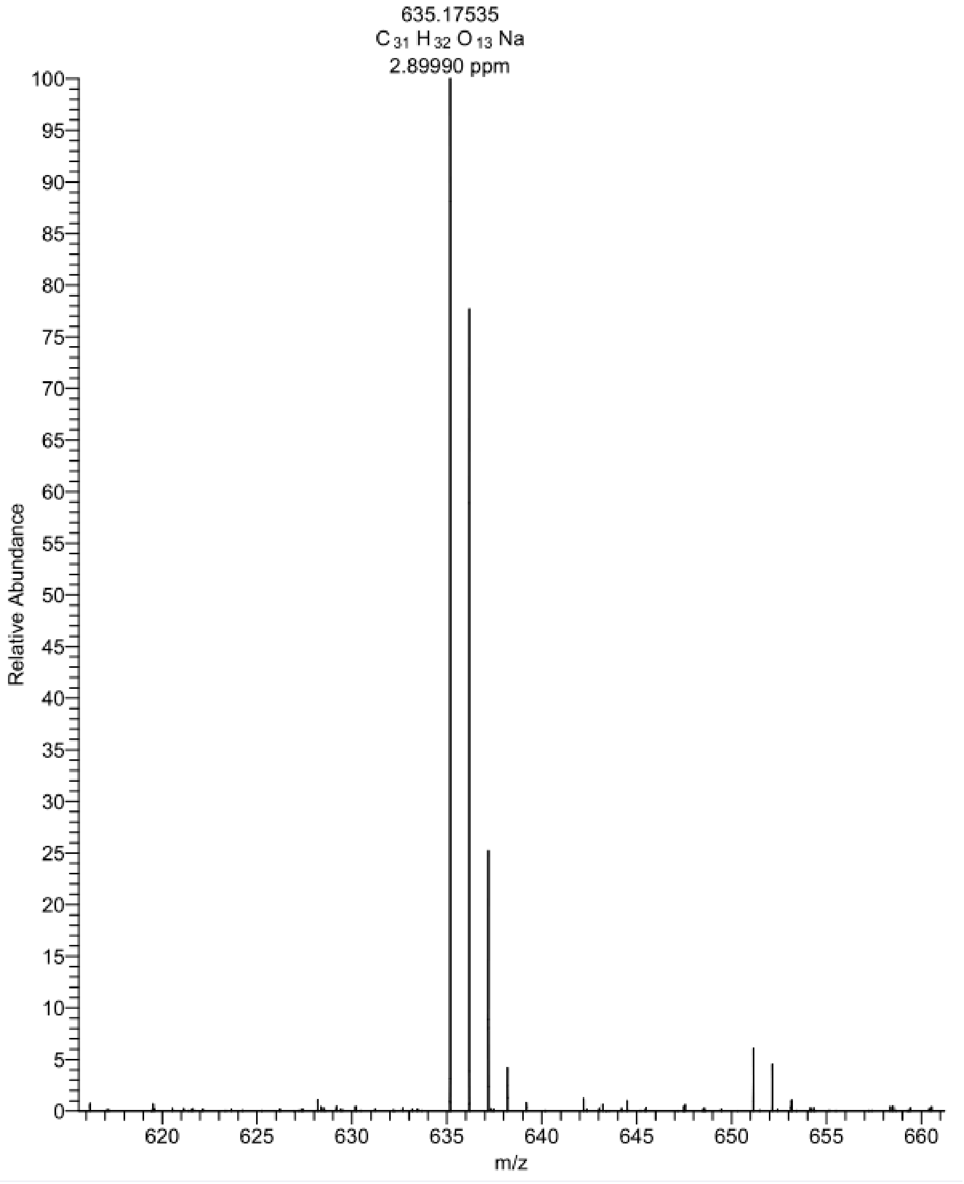

Figure S23. ^{1}H NMR (600 MHz) spectrum of compound 4.

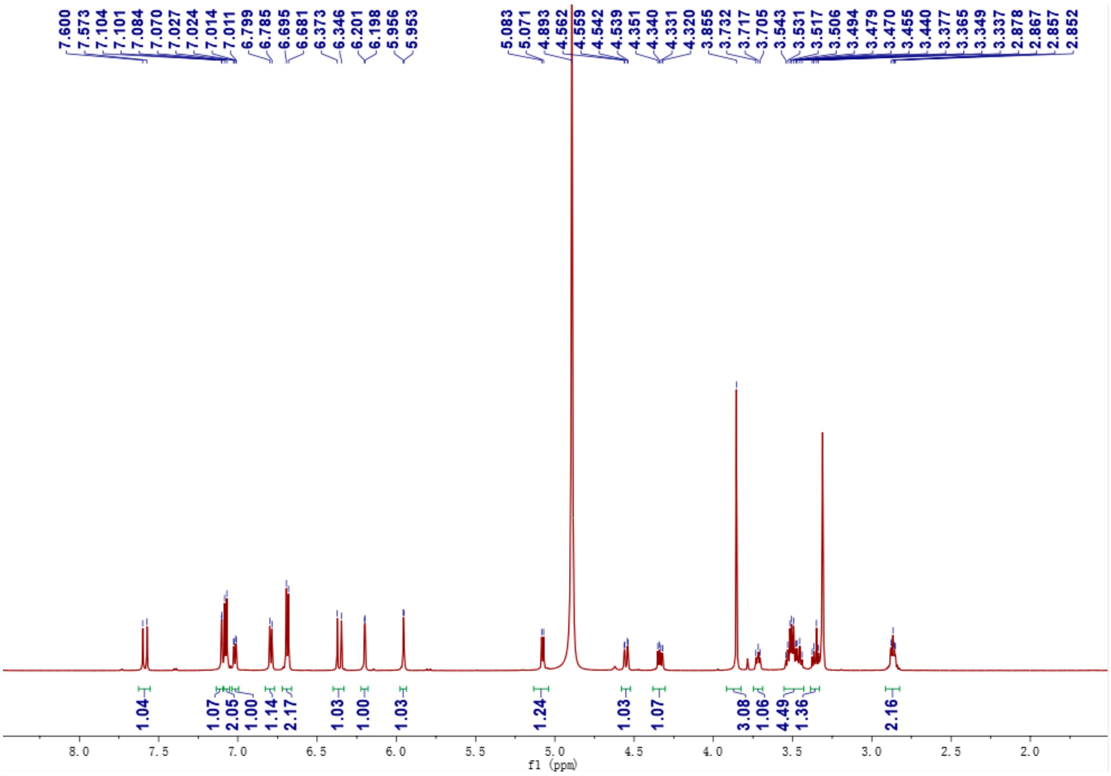

Figure S24. ^{13}C NMR and DEPT (150 MHz) spectra of compound 4.

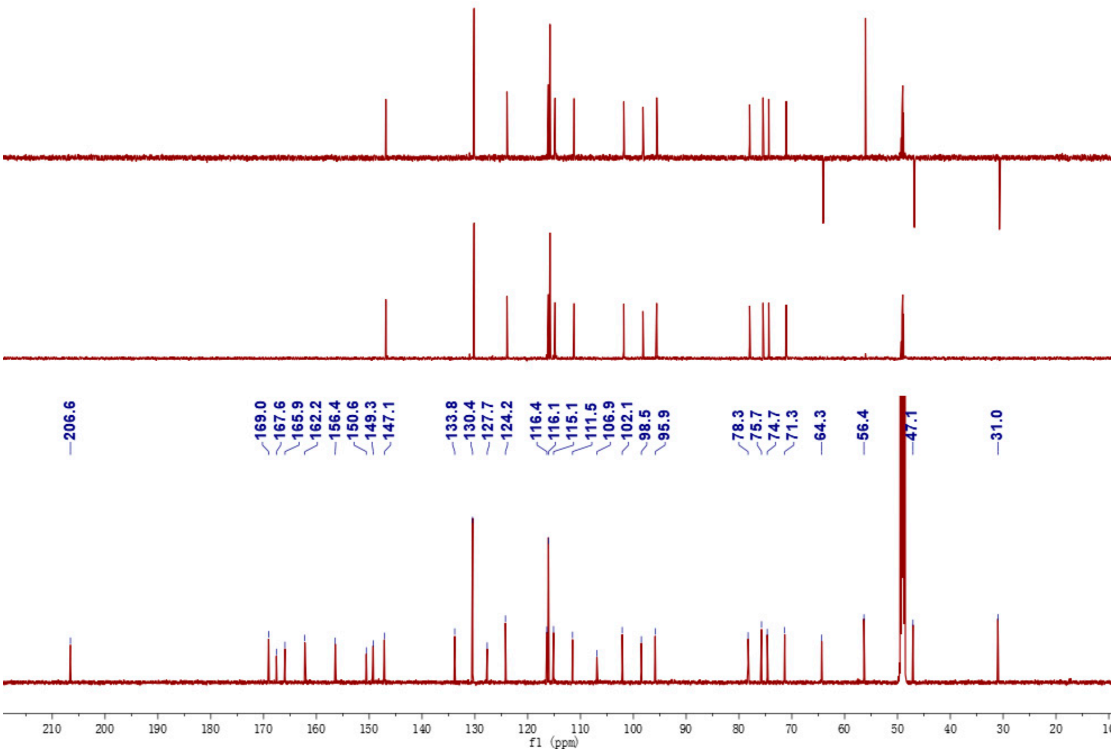

Figure S25. ^{1}H-^{1}H COSY spectrum of compound 4.

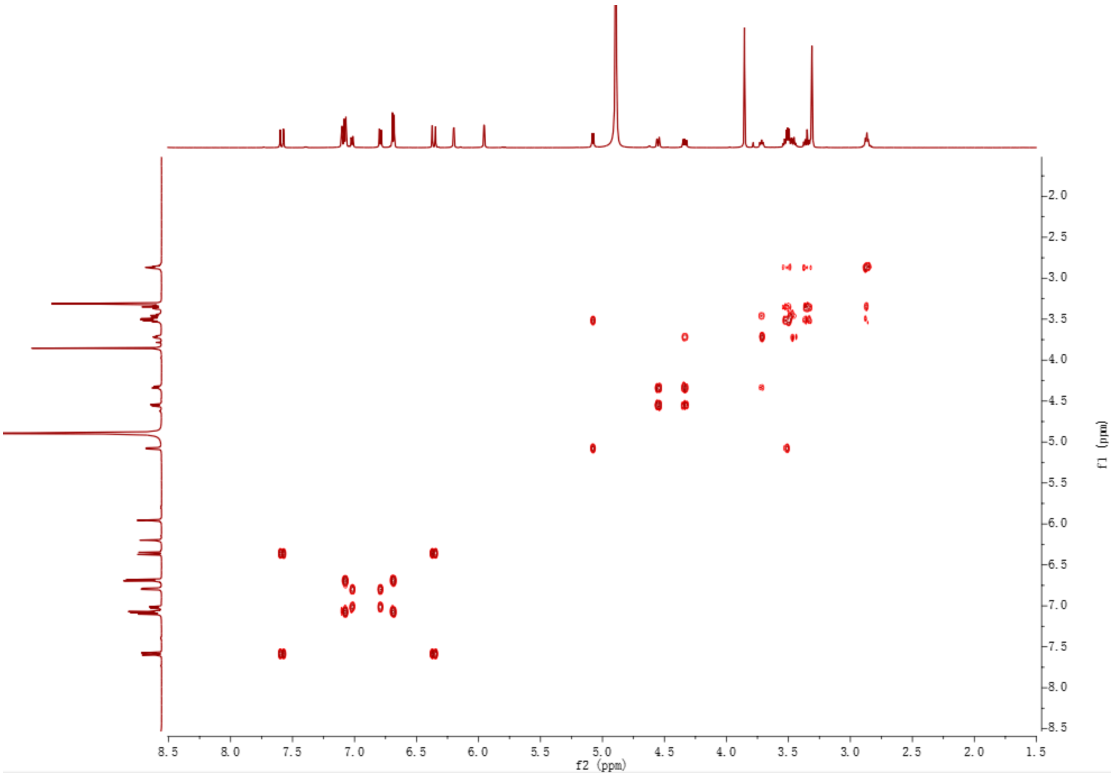

Figure S26. HSQC spectrum of compound 4.

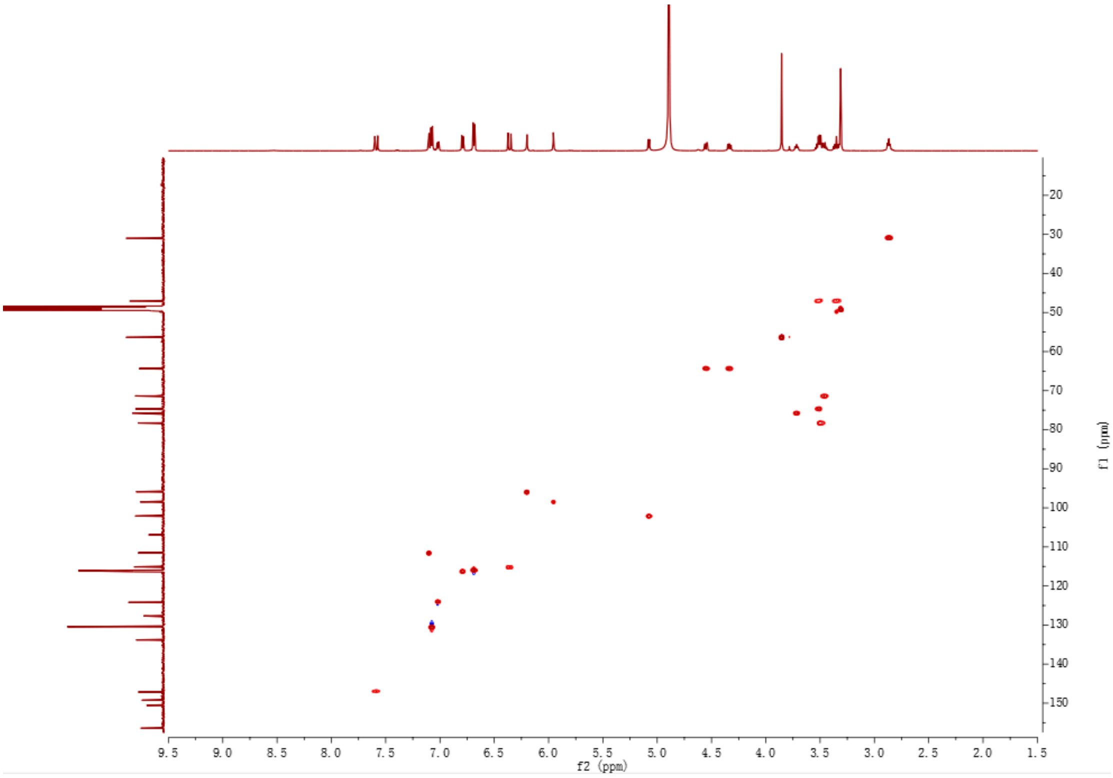

Figure S27. HMBC spectrum of compound 4.

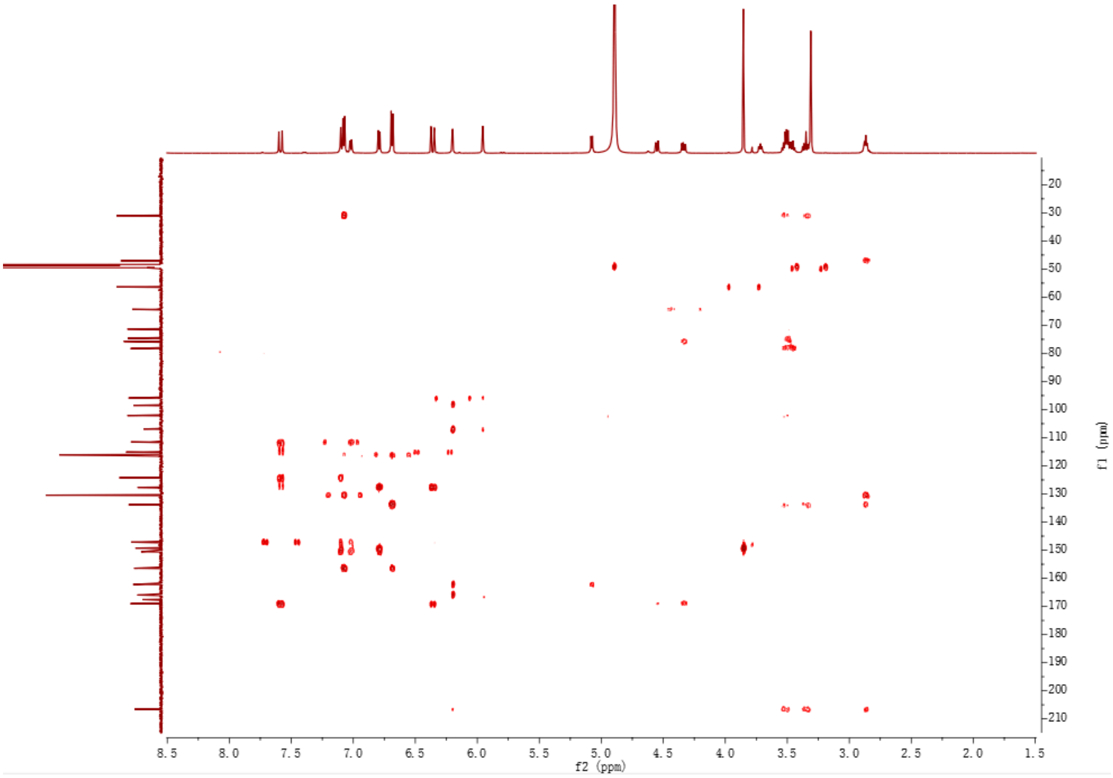

Figure S28. ROESY spectrum of compound 4.

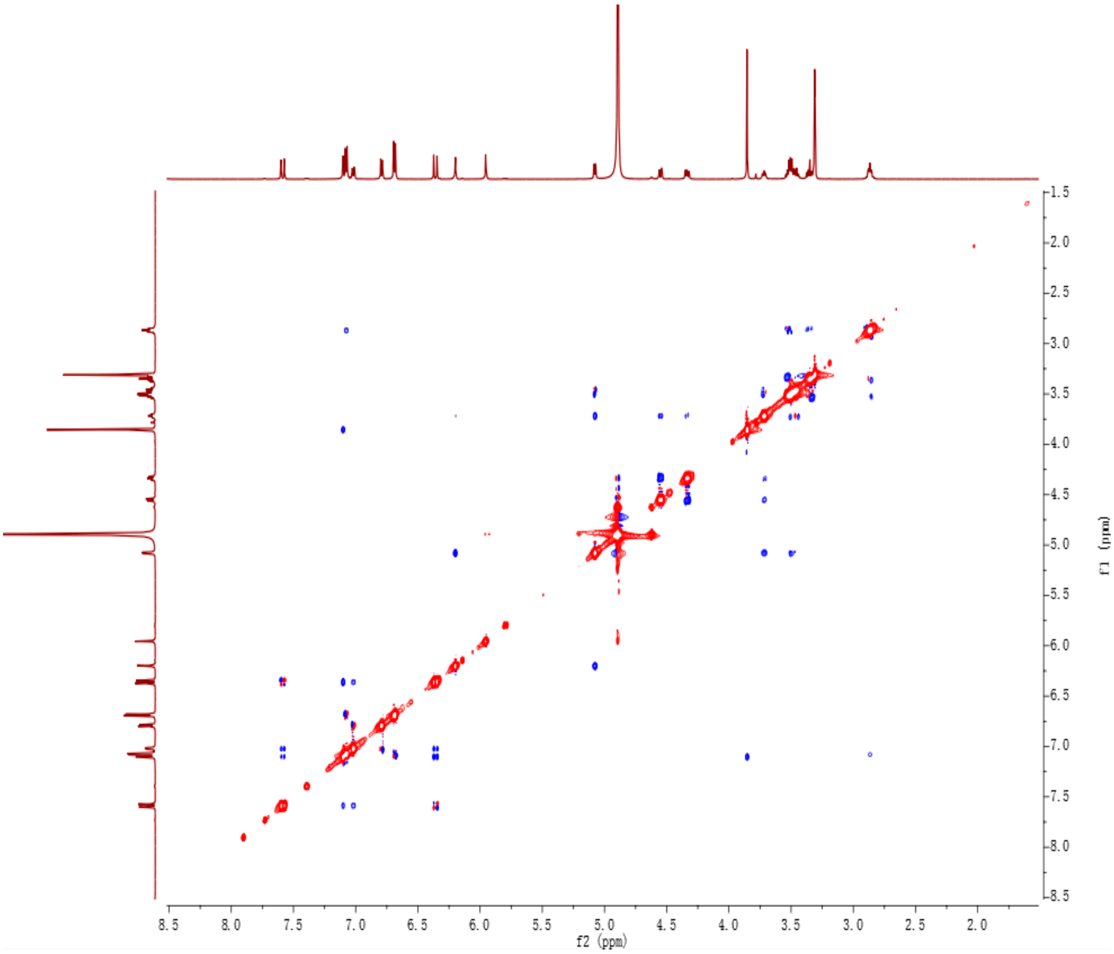

Figure S29. HRESIMS spectroscopic data of compound 5.

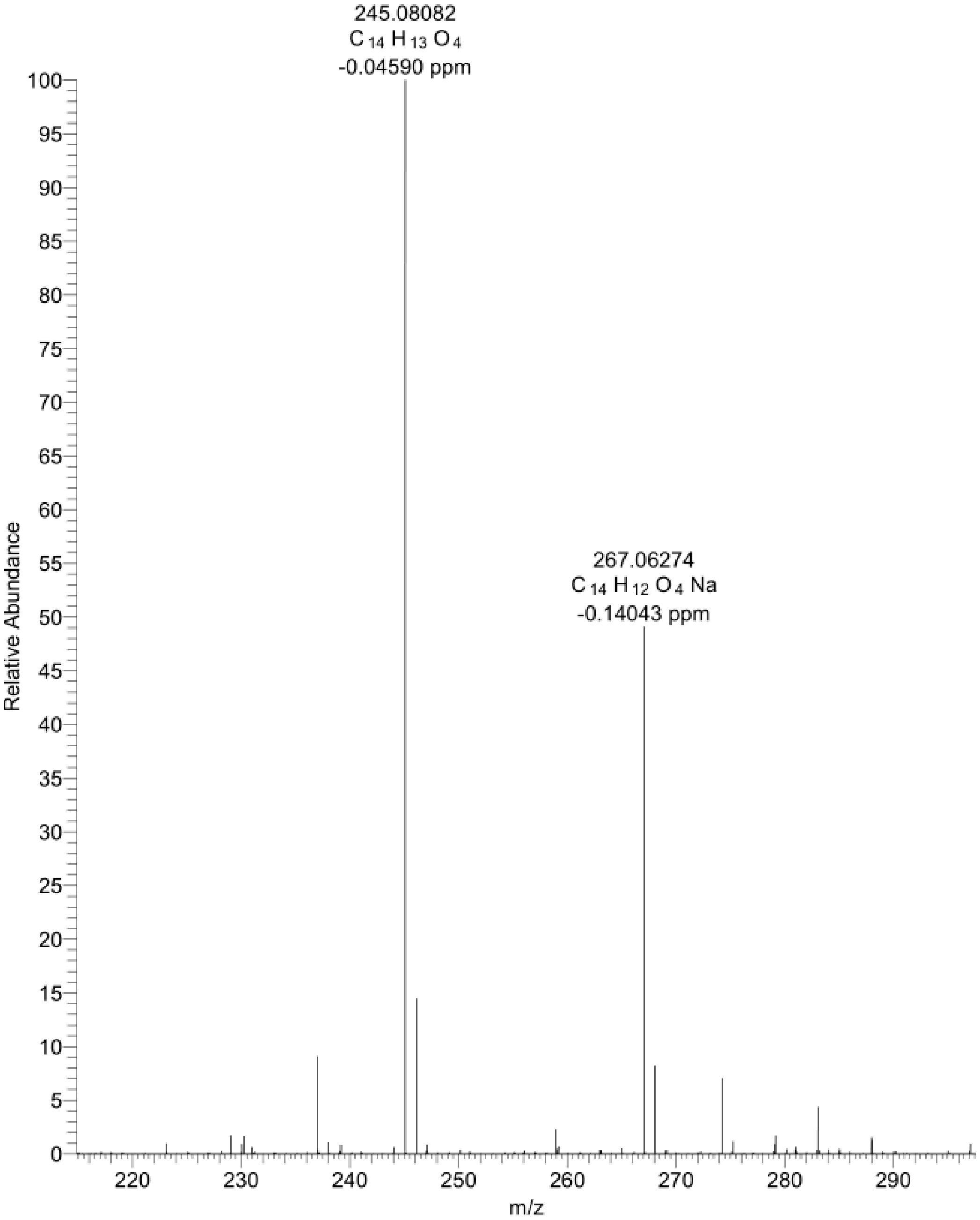

Figure S30. ^{1}H NMR (600 MHz) spectrum of compound 5.

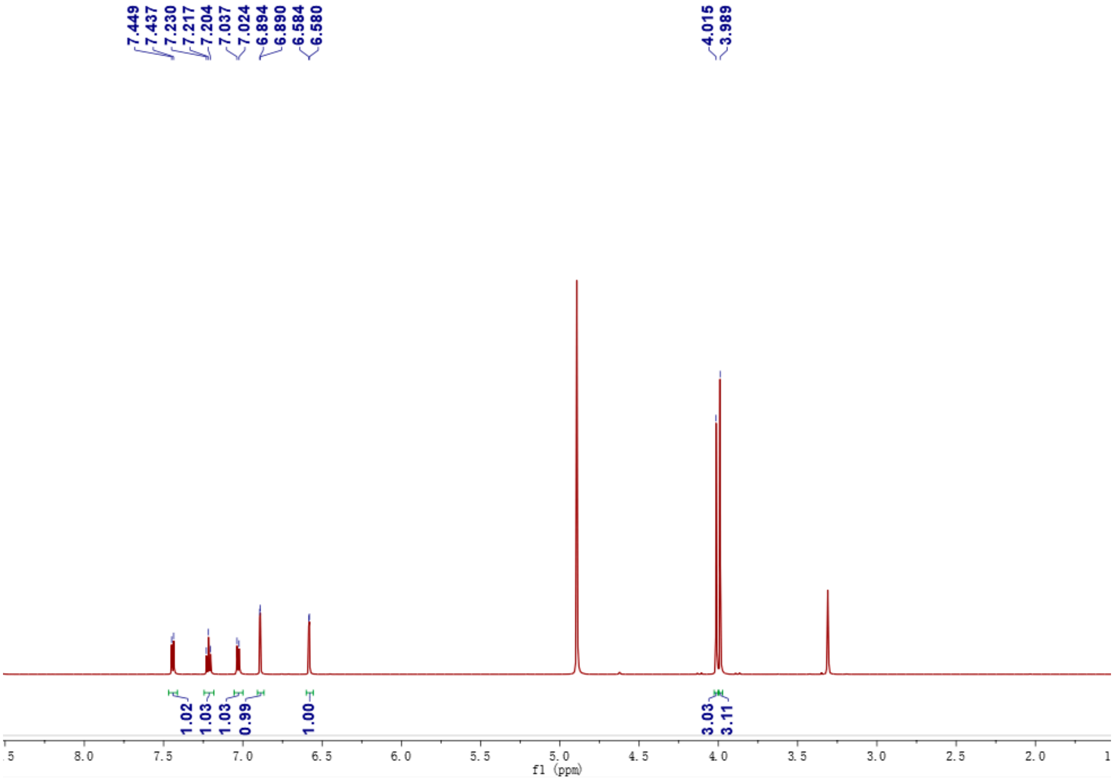

Figure S31. ^{13}C NMR and DEPT (150 MHz) spectra of compound 5.

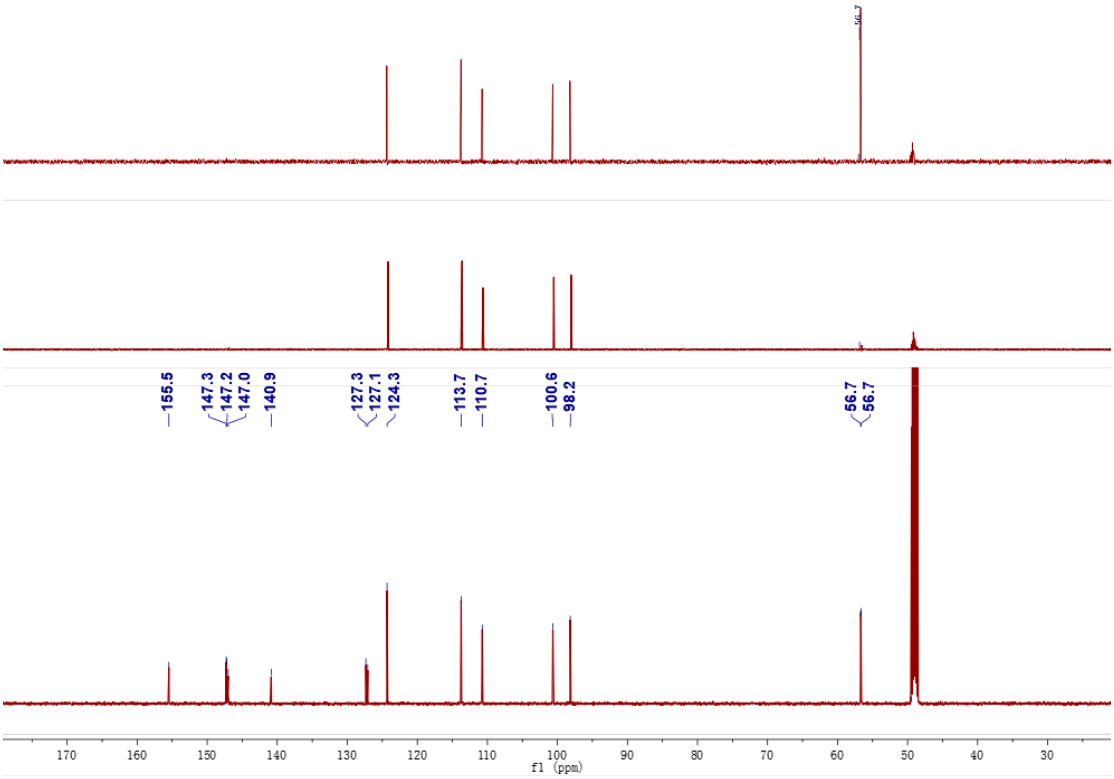

Figure S32. ^{1}H-^{1}H COSY spectrum of compound 5.

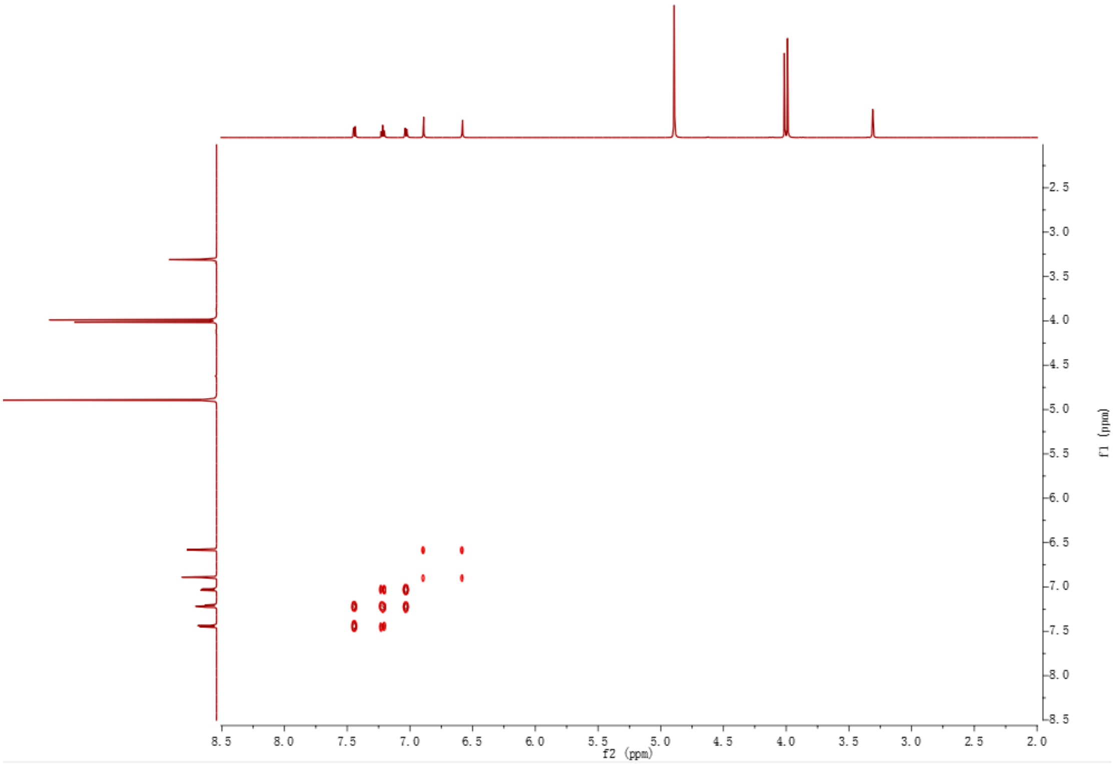

Figure S33. HSQC spectrum of compound 5.

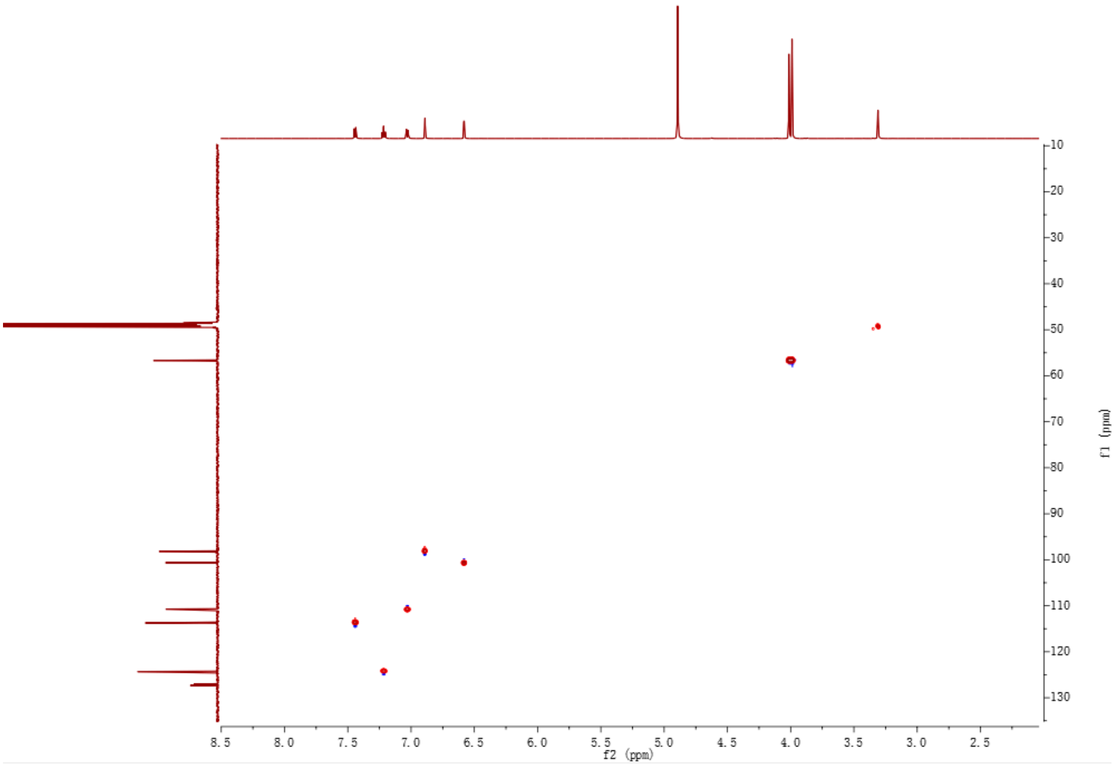

Figure S34. HMBC spectrum of compound 5.

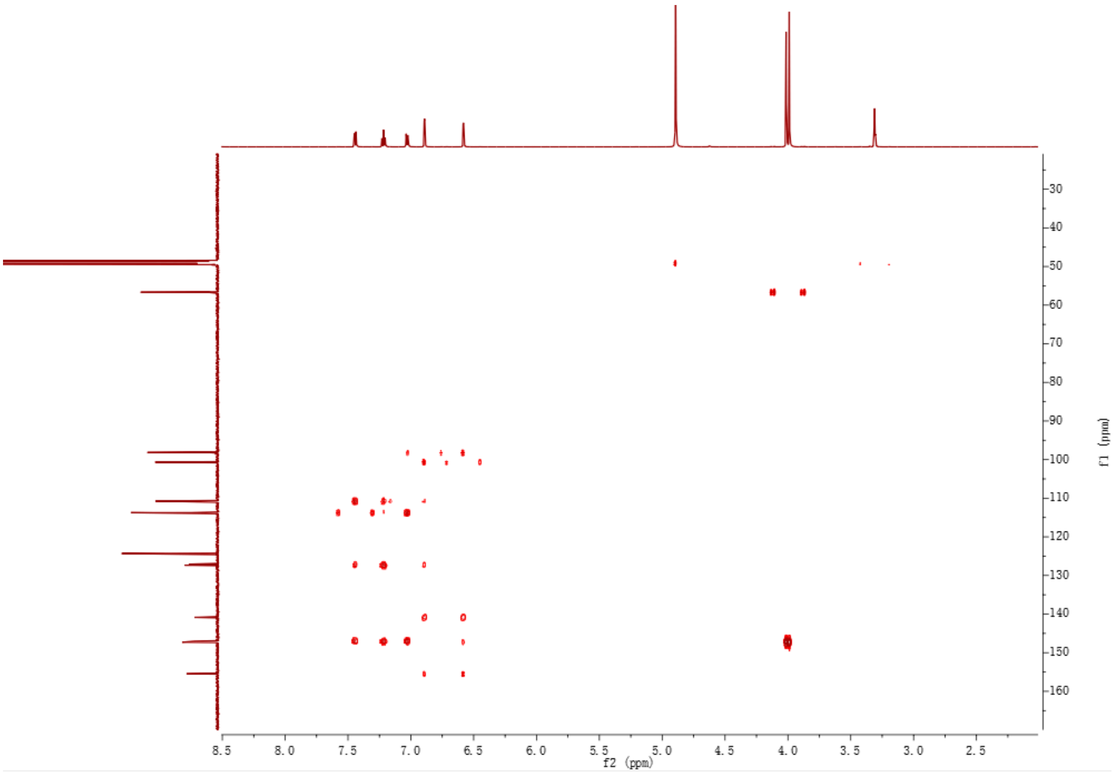

Figure S35. ROESY spectrum of compound 5.

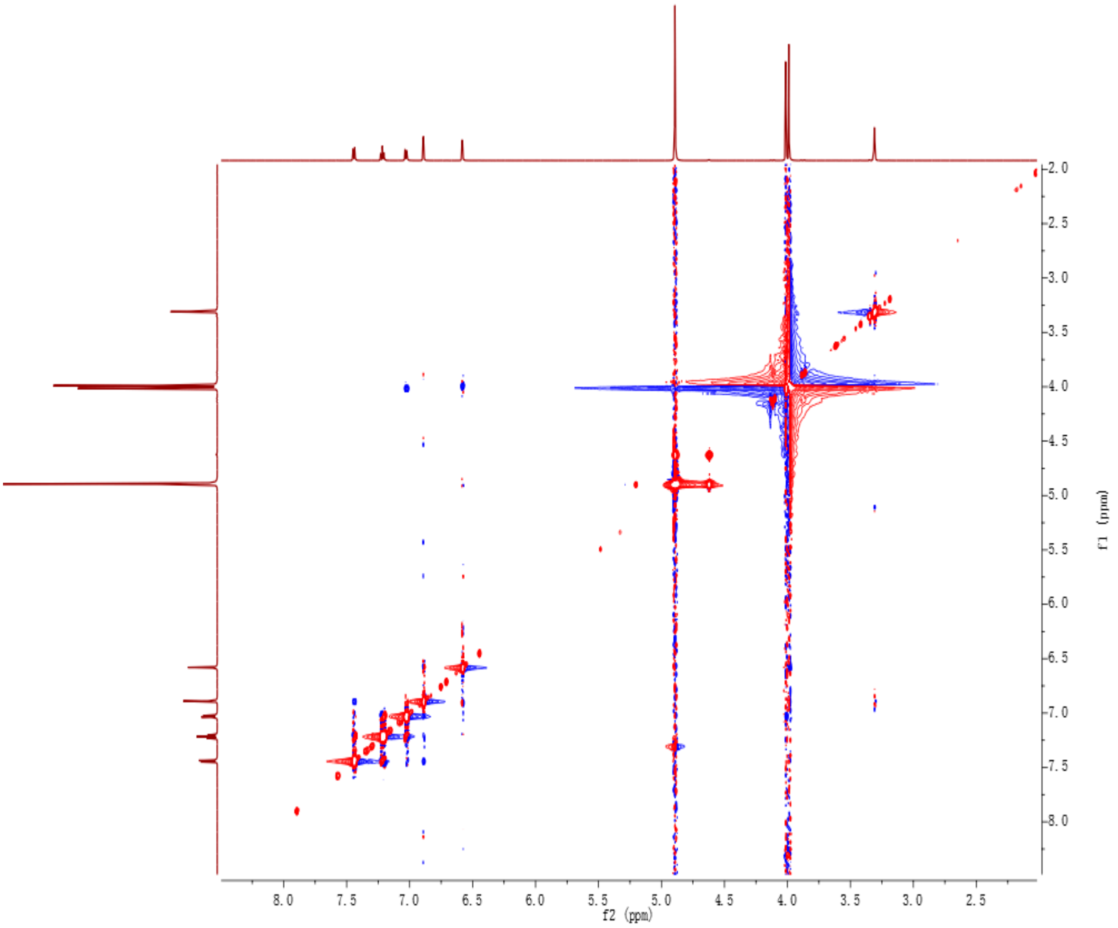

Figure S36. HRESIMS spectroscopic data of compound 6.

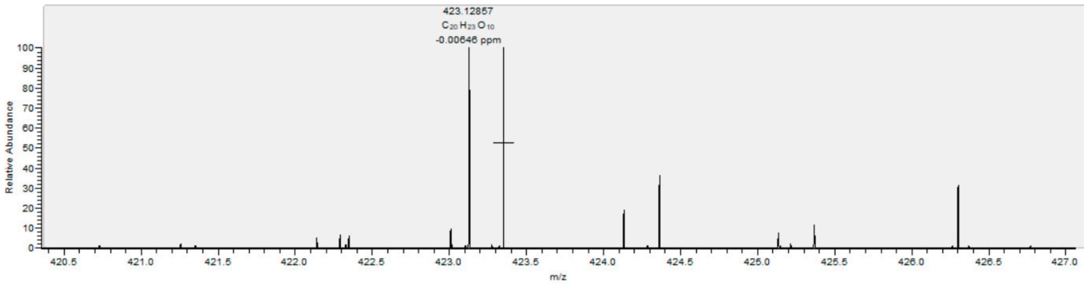

Figure S37. ^{1}H NMR (600 MHz) spectrum of compound 6.

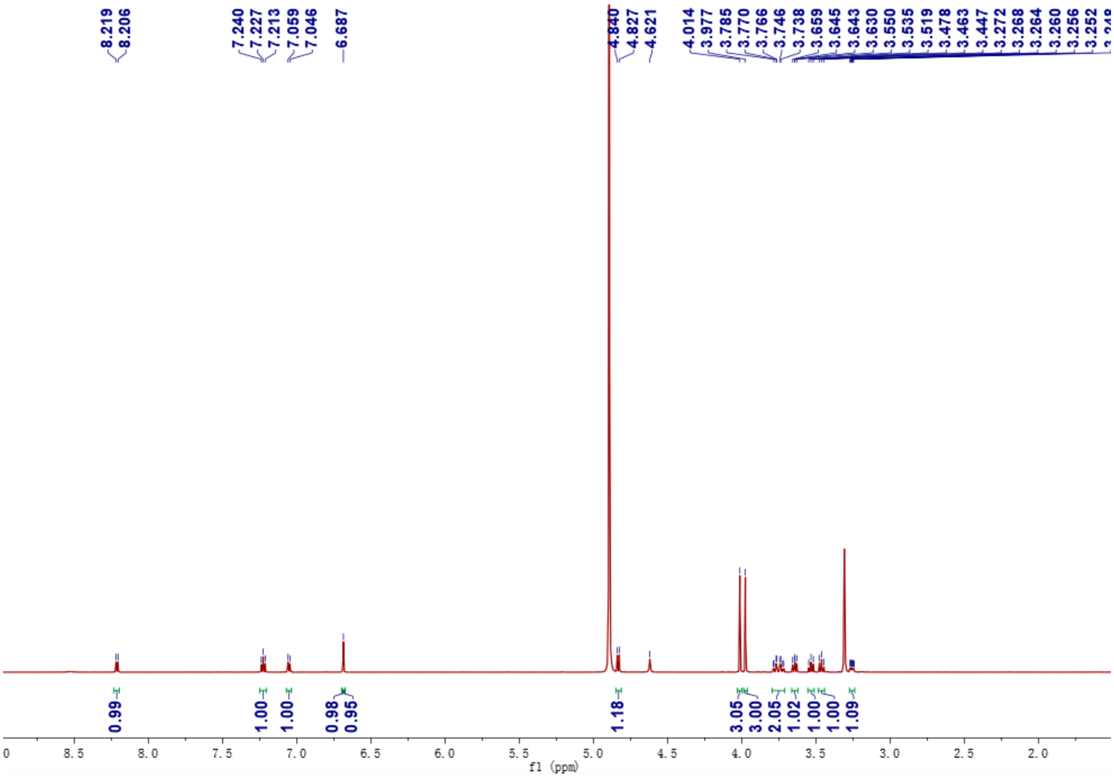

Figure S38. ^{13}C NMR and DEPT (150 MHz) spectra of compound 6.

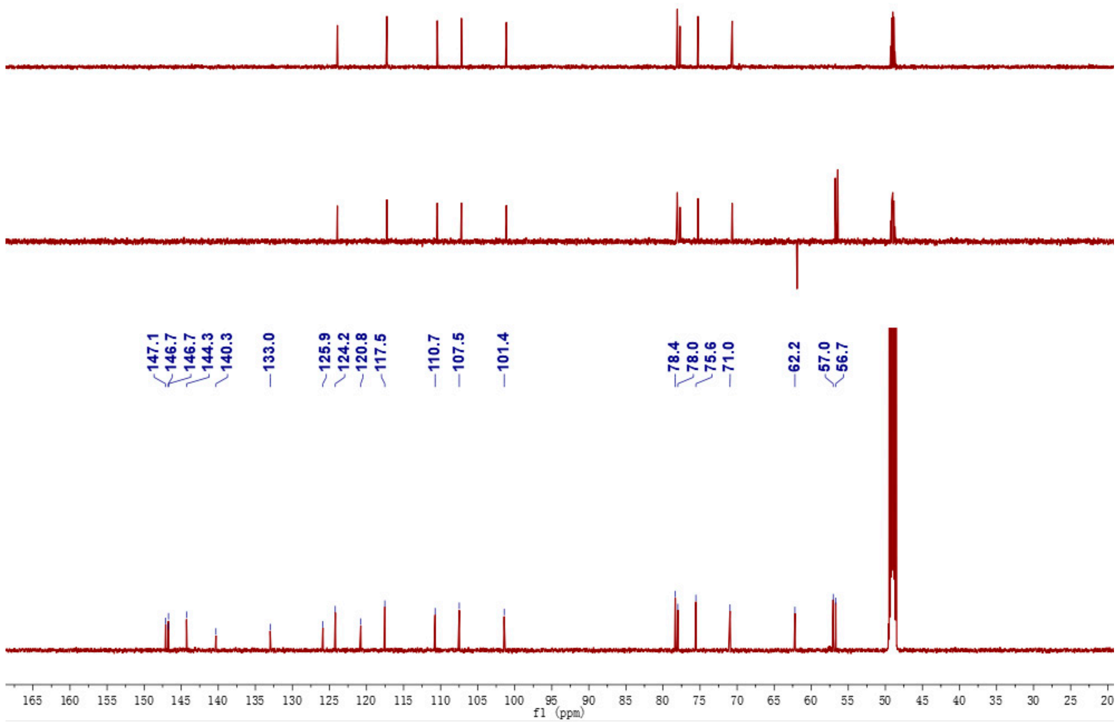

Figure S39. ^{1}H-^{1}H COSY spectrum of compound 6.

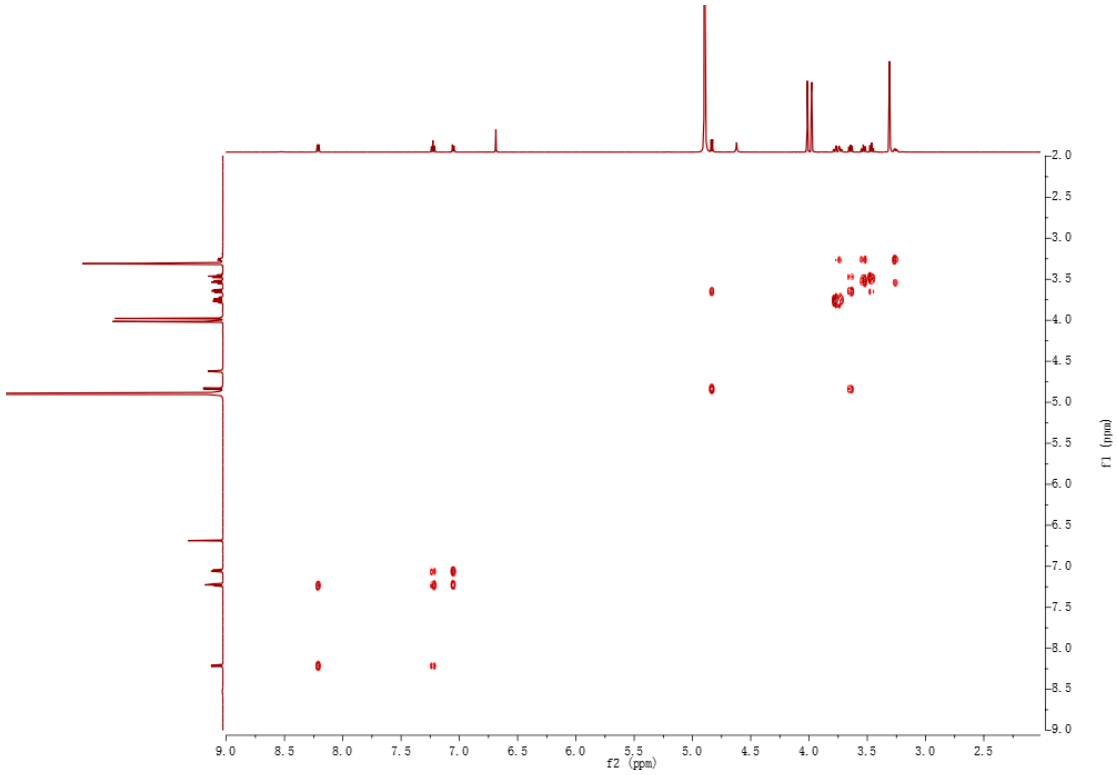

Figure S40. HSQC spectrum of compound 6.

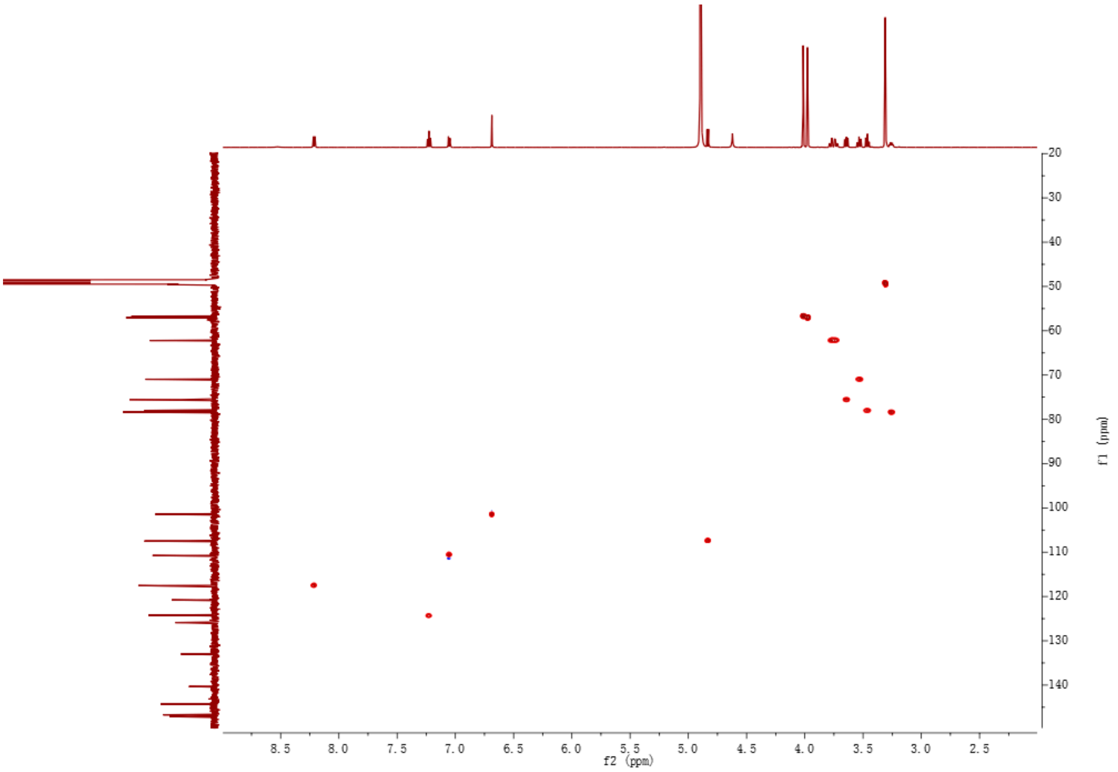

Figure S41. HMBC spectrum of compound 6.

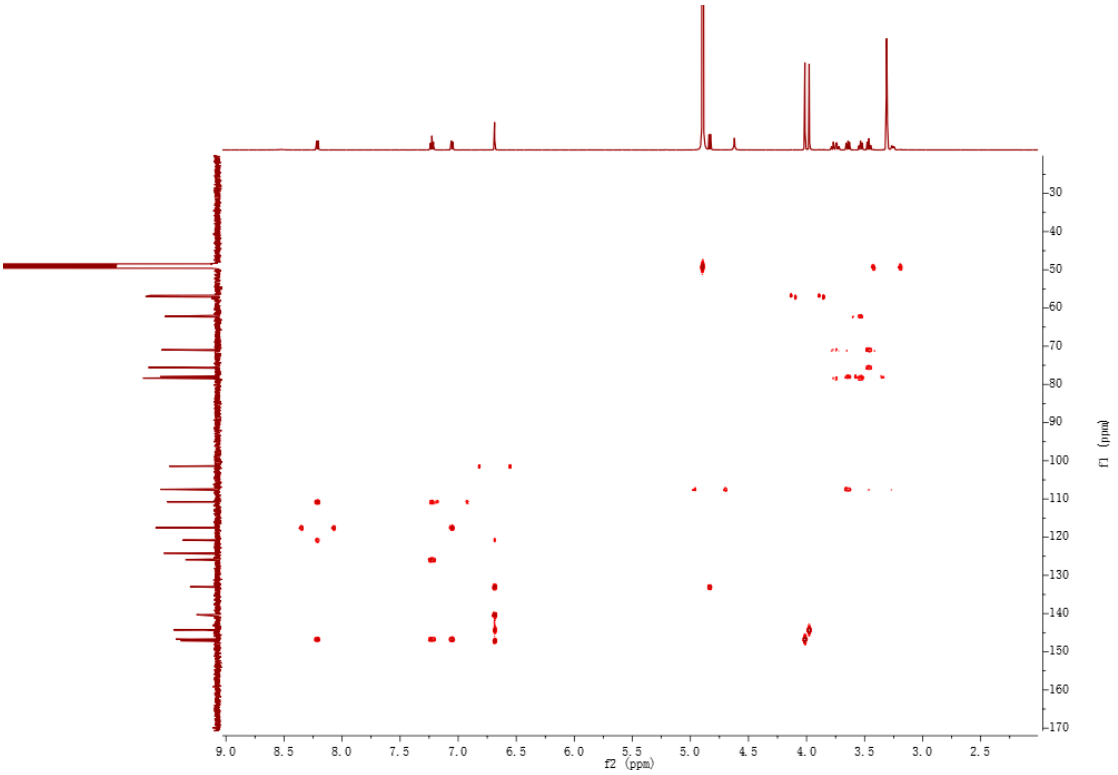

Figure S42. ROESY spectrum of compound 6.

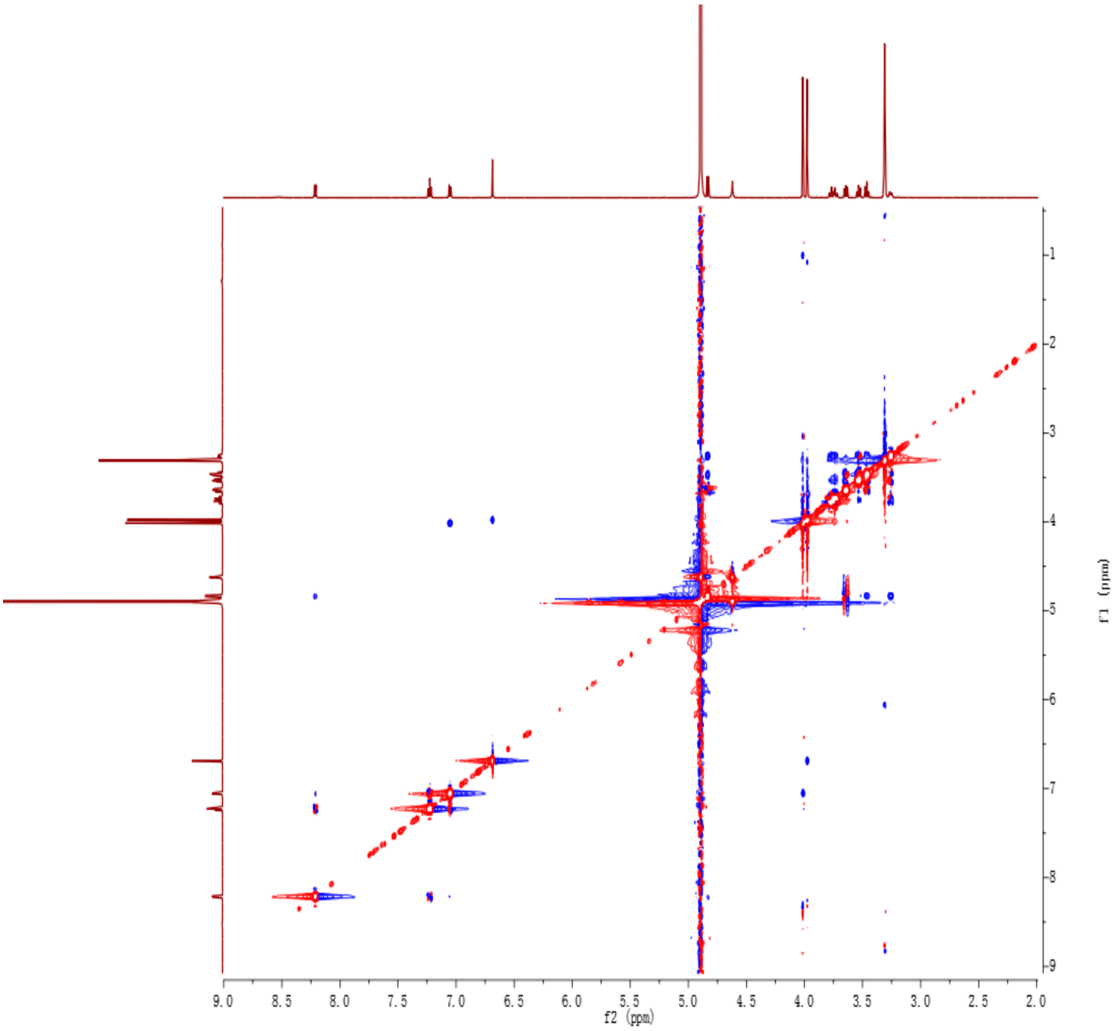

Figure S43. HRESIMS spectroscopic data of compound 7.

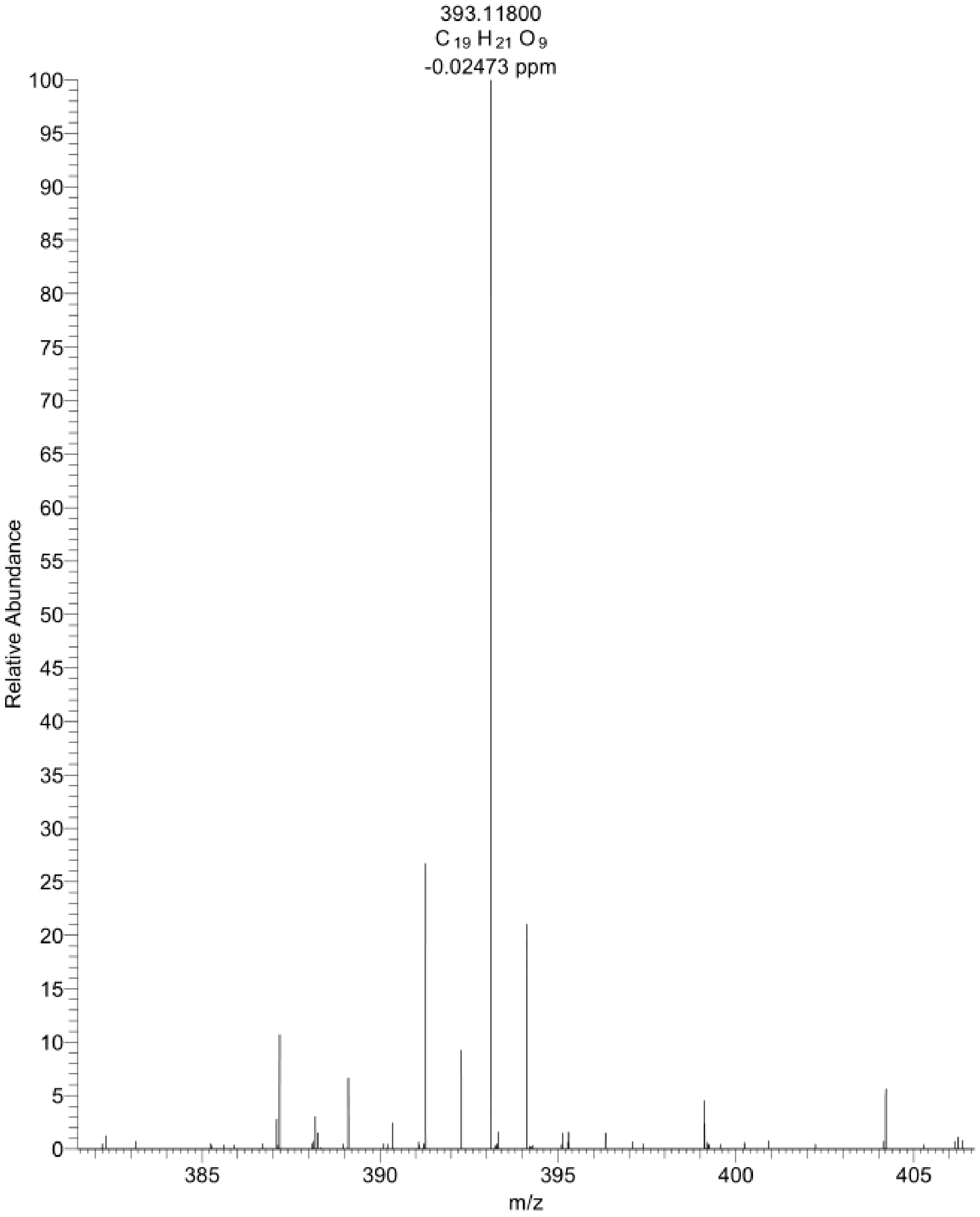

Figure S44. ^{1}H NMR (600 MHz) spectrum of compound 7.

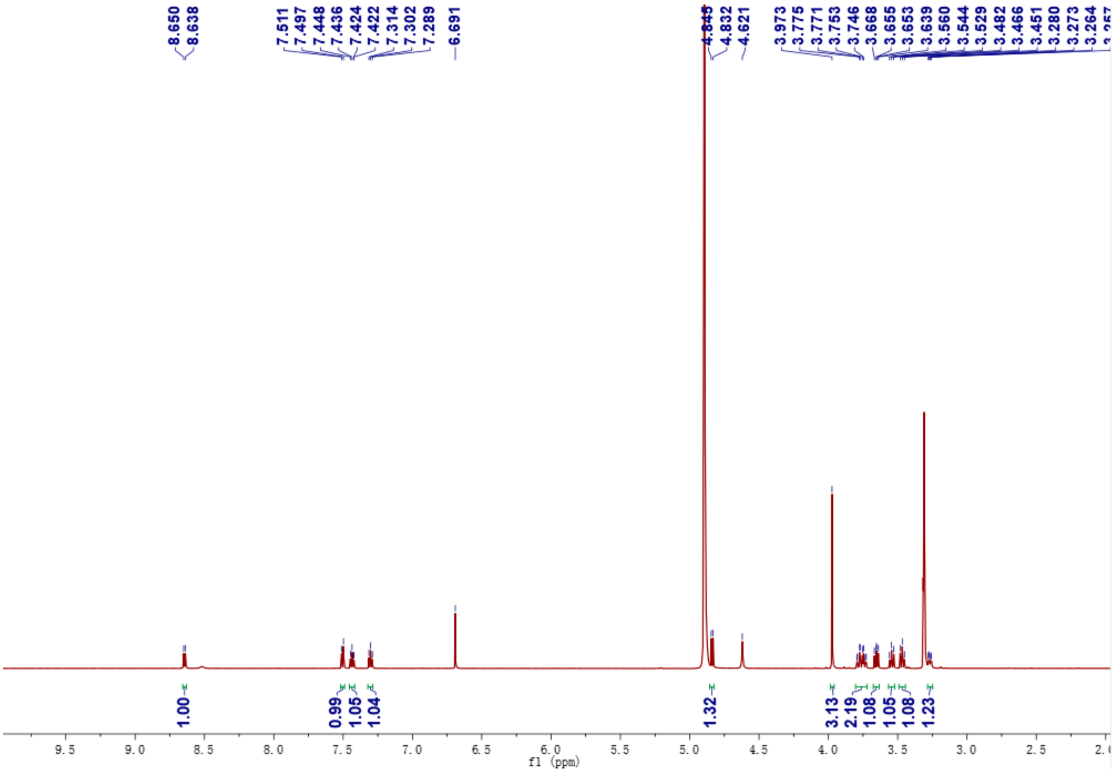

Figure S45. ^{13}C NMR and DEPT (150 MHz) spectra of compound 7.

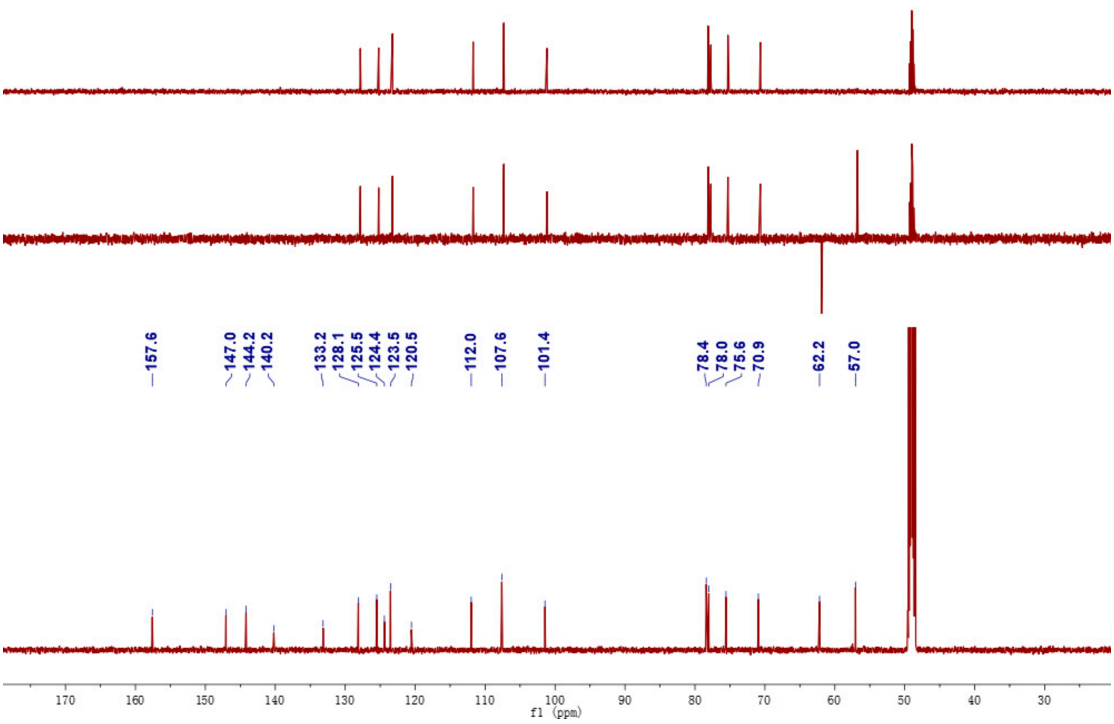

Figure S46. ^{1}H-^{1}H COSY spectrum of compound 7.

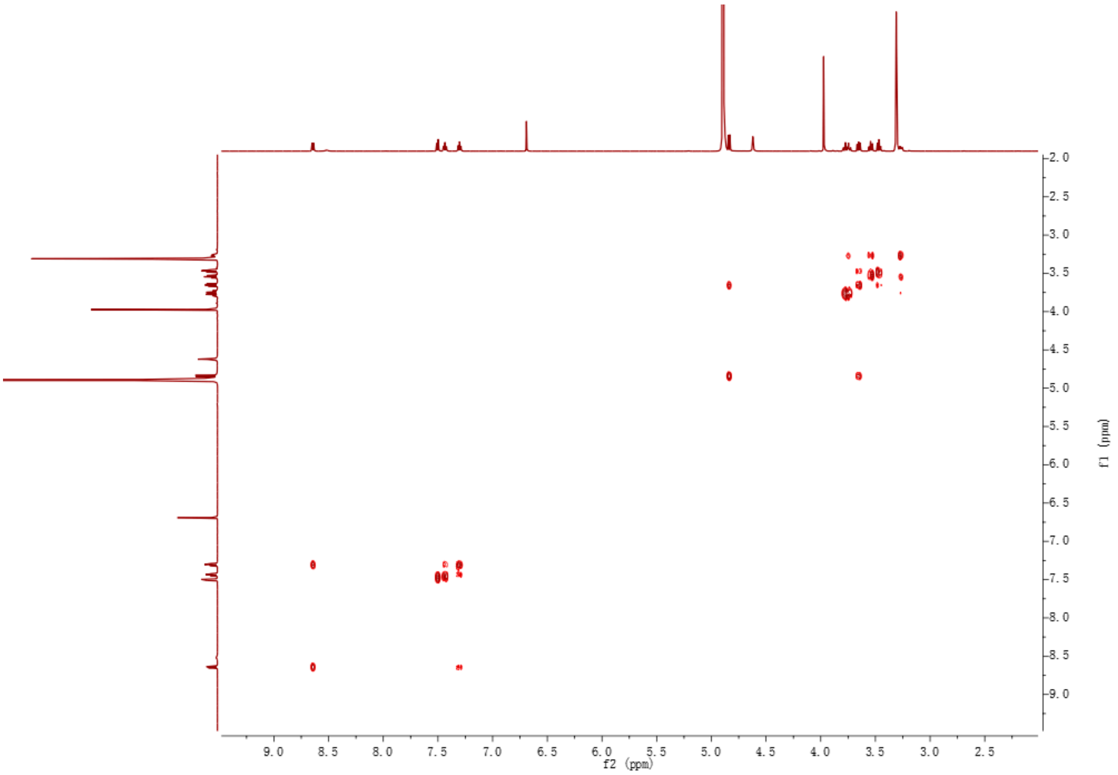

Figure S47. HSQC spectrum of compound 7.

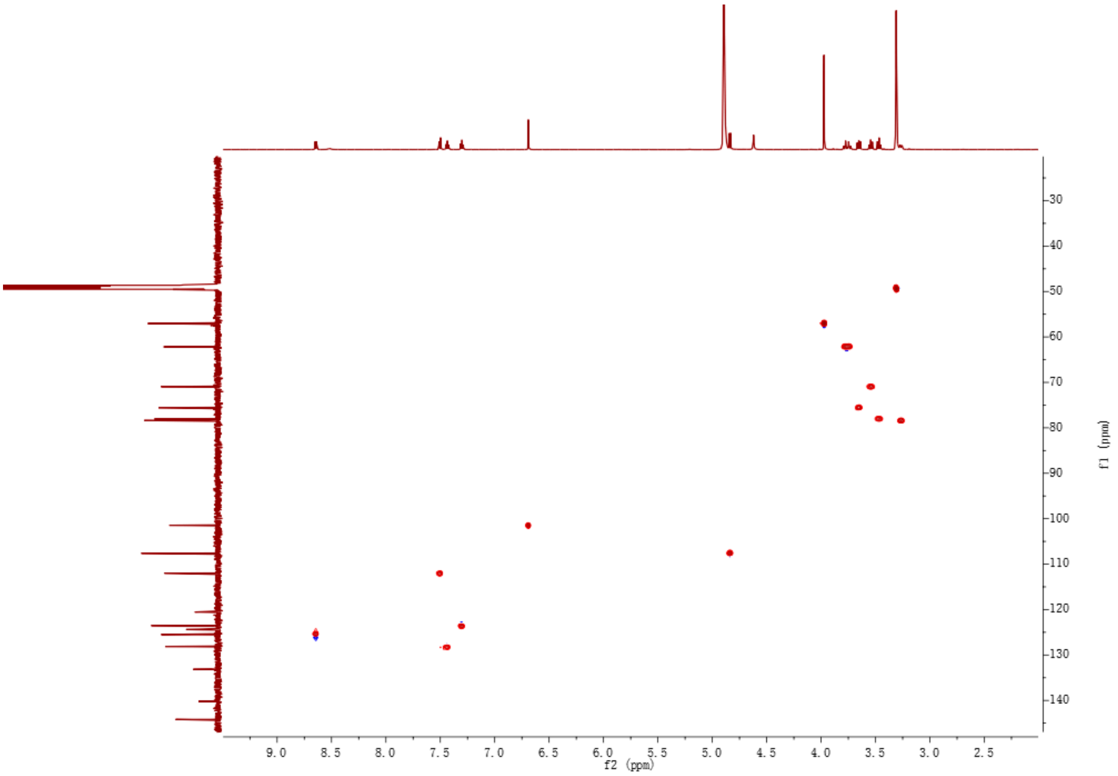

Figure S48. HMBC spectrum of compound 7.

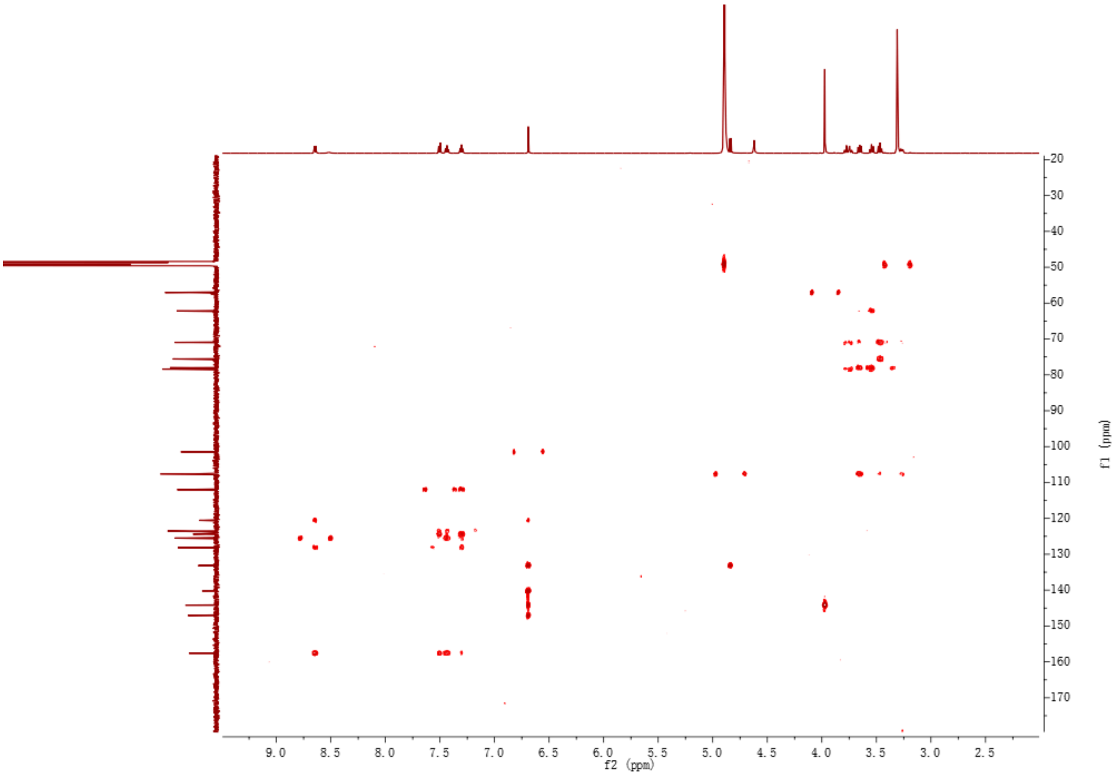

Figure S49. ROESY spectrum of compound 7.

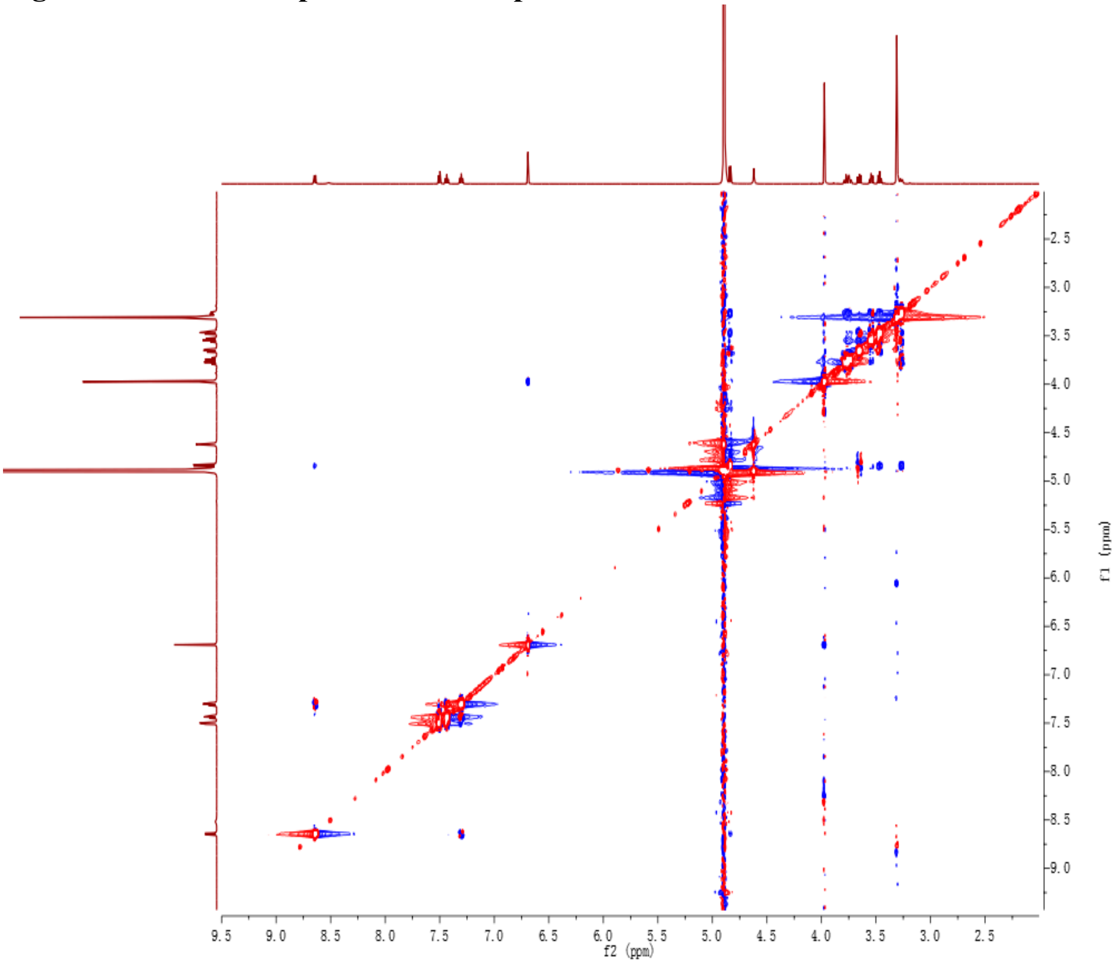

Figure S50. HRESIMS spectroscopic data of compound 8.

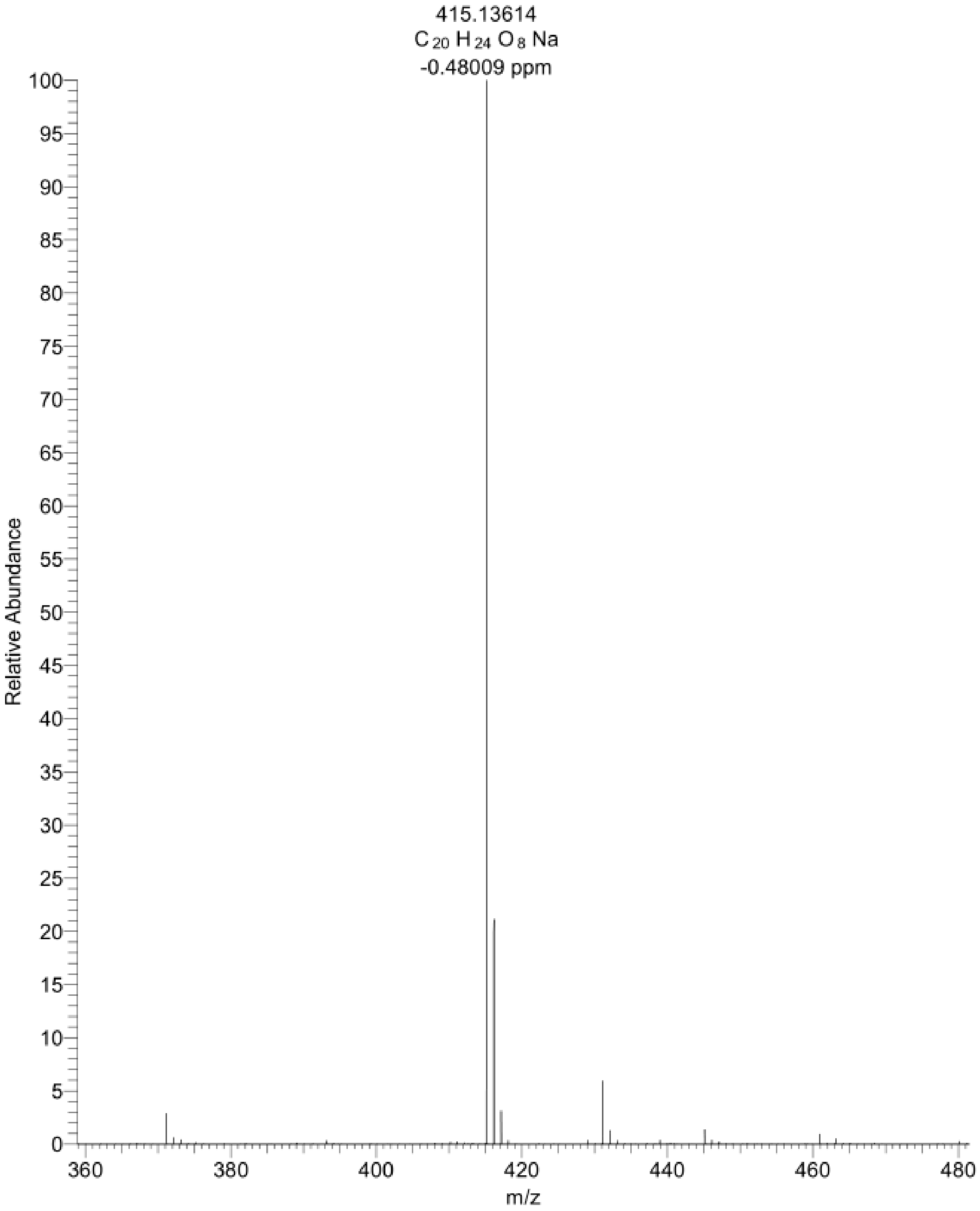

Figure S51. ^{1}H NMR (600 MHz) spectrum of compound 8.

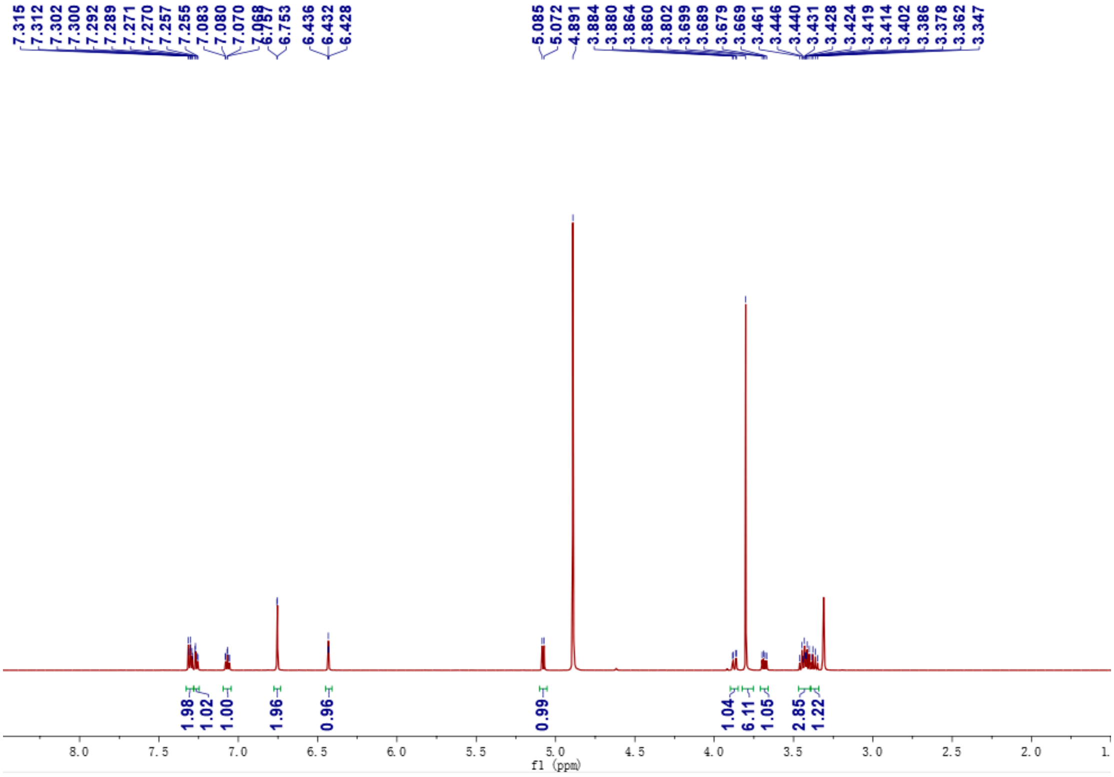

Figure S52. ^{13}C NMR and DEPT (150 MHz) spectra of compound 8.

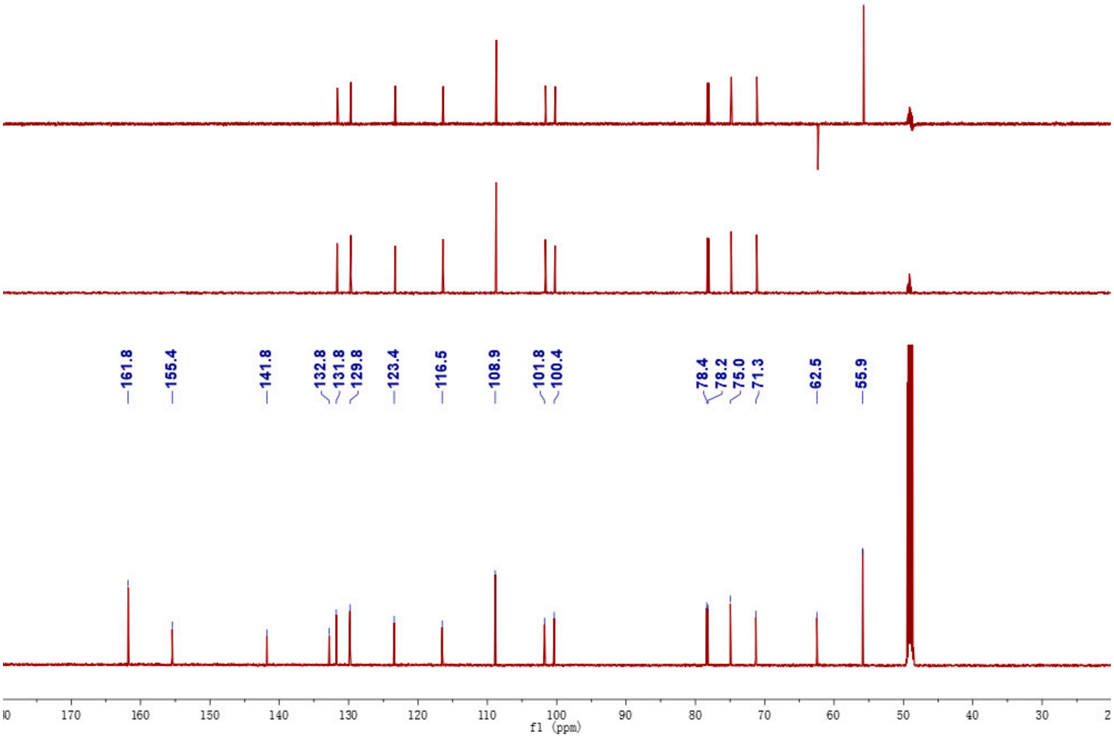

Figure S53. ^{1}H-^{1}H COSY spectrum of compound 8.

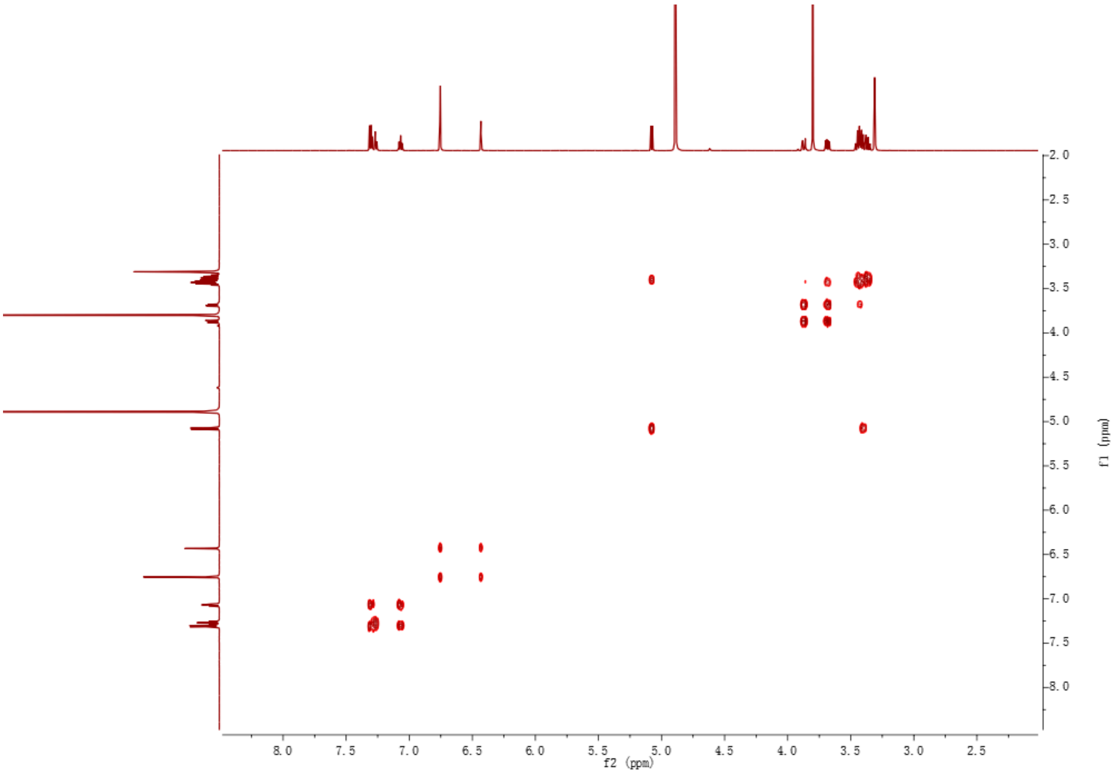

Figure S54. HSQC spectrum of compound 8.

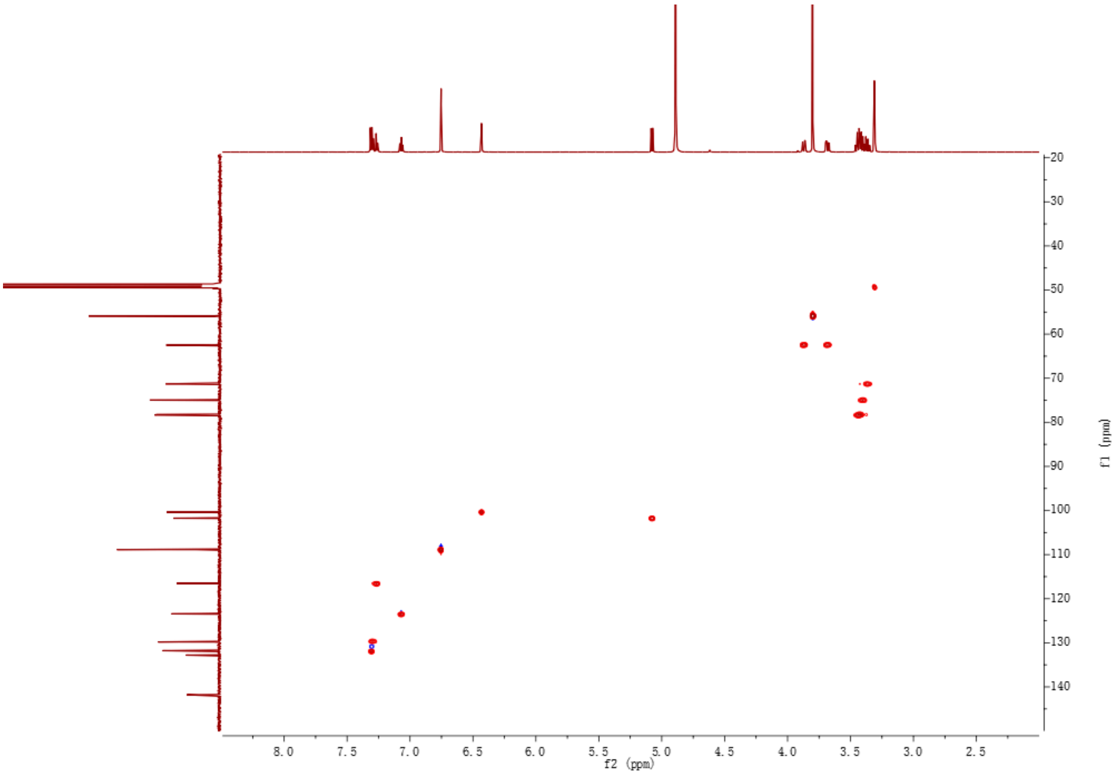

Figure S55. HMBC spectrum of compound 8.

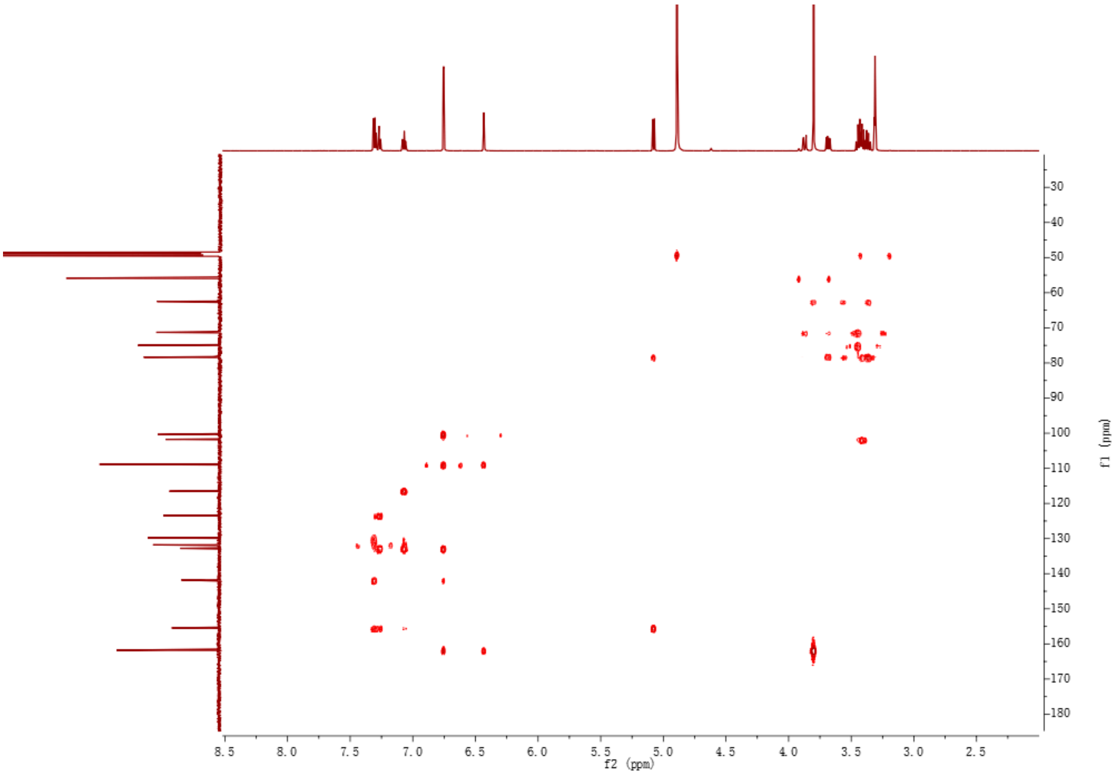

Figure S56. ROESY spectrum of compound 8.

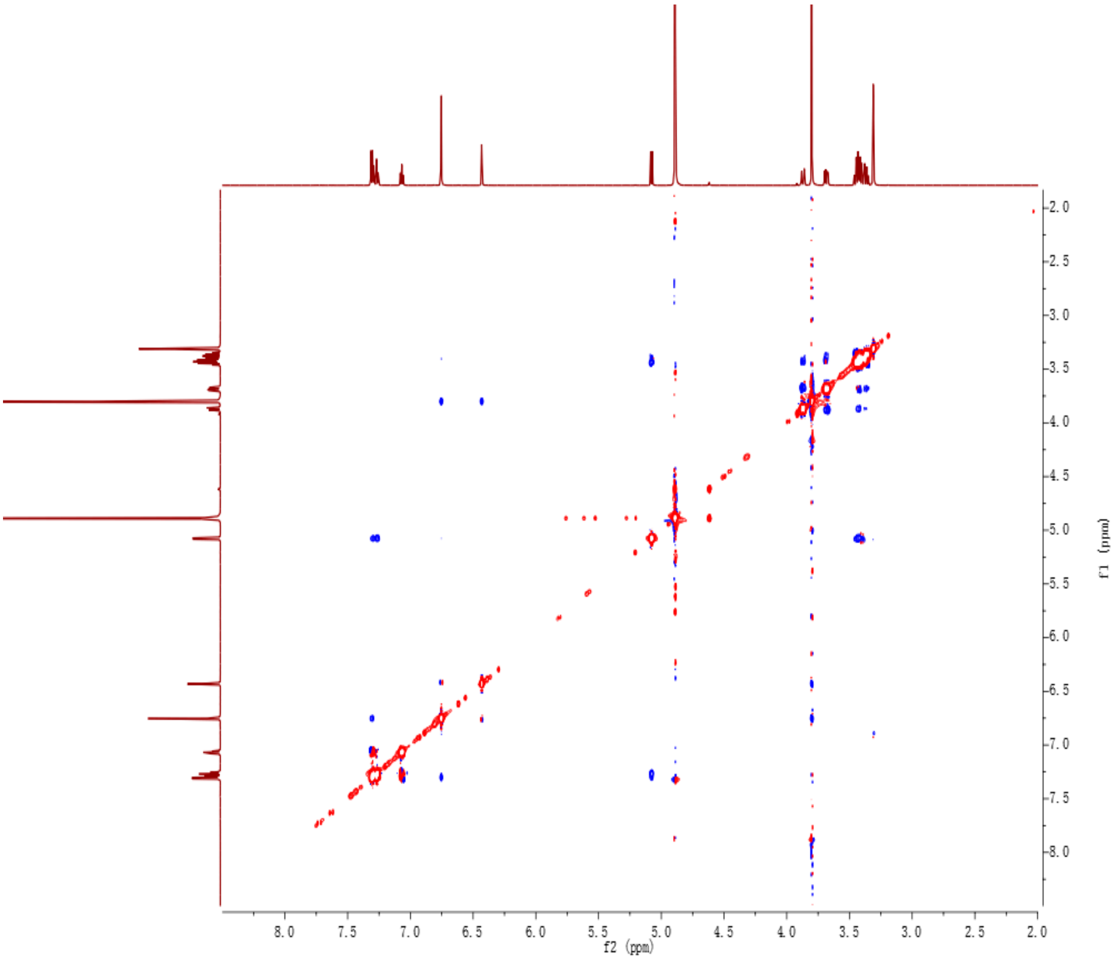

Figure S57. HRESIMS spectroscopic data of compound 9.

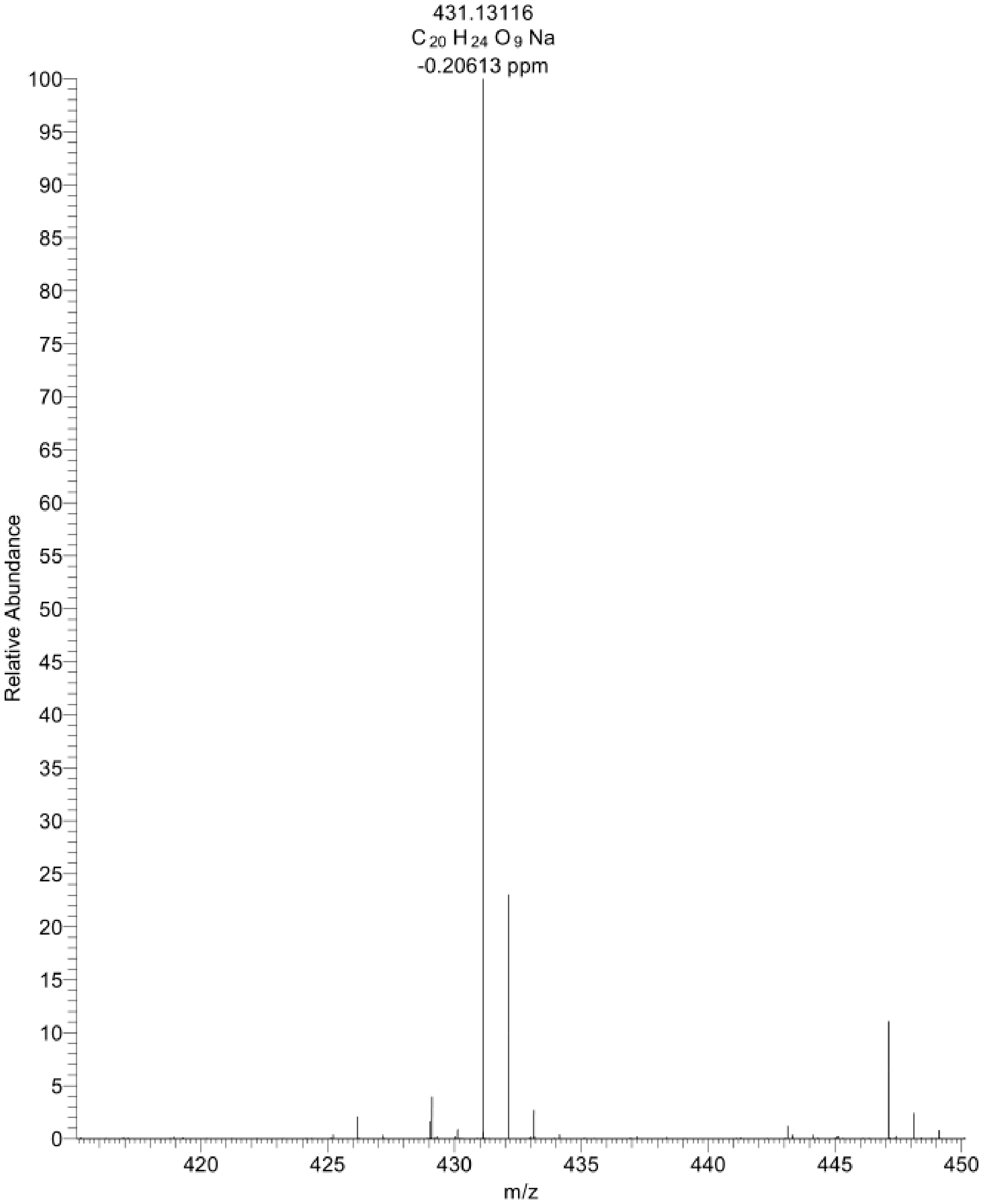

Figure S58. ^{1}H NMR (600 MHz) spectrum of compound 9.

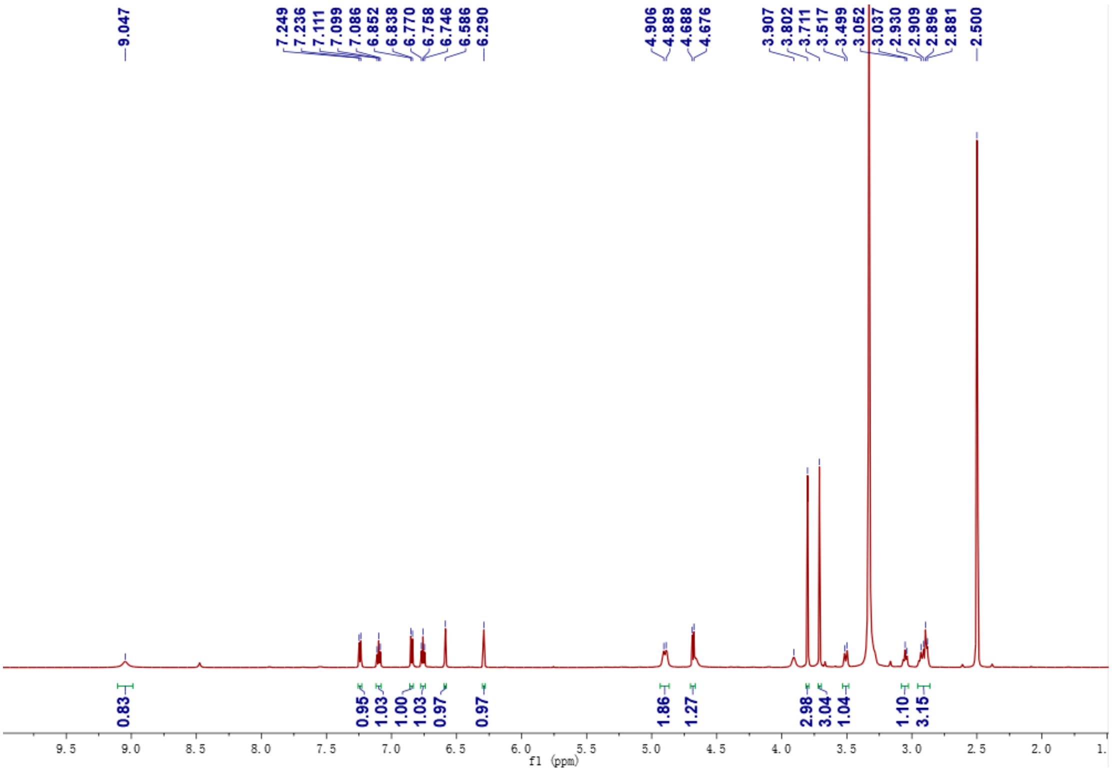

Figure S59. ^{13}C NMR and DEPT (150 lMHz) spectra of compound 9.

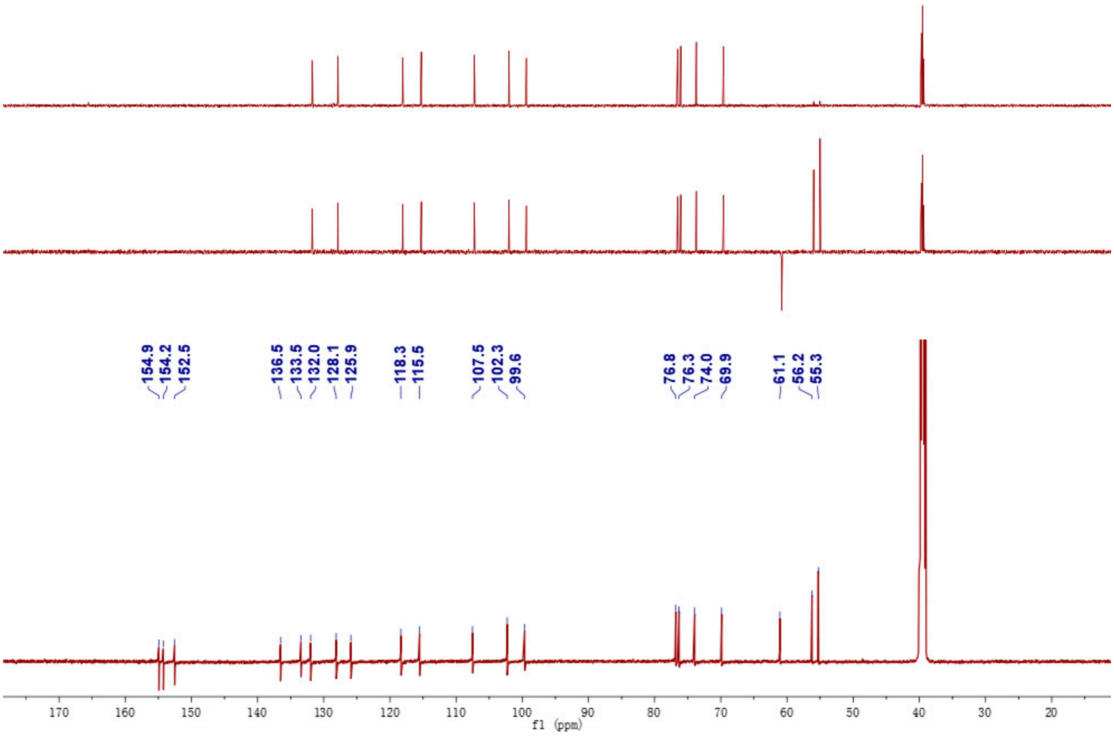

Figure S60. ^{1}H-^{1}H COSY spectrum of compound 9.

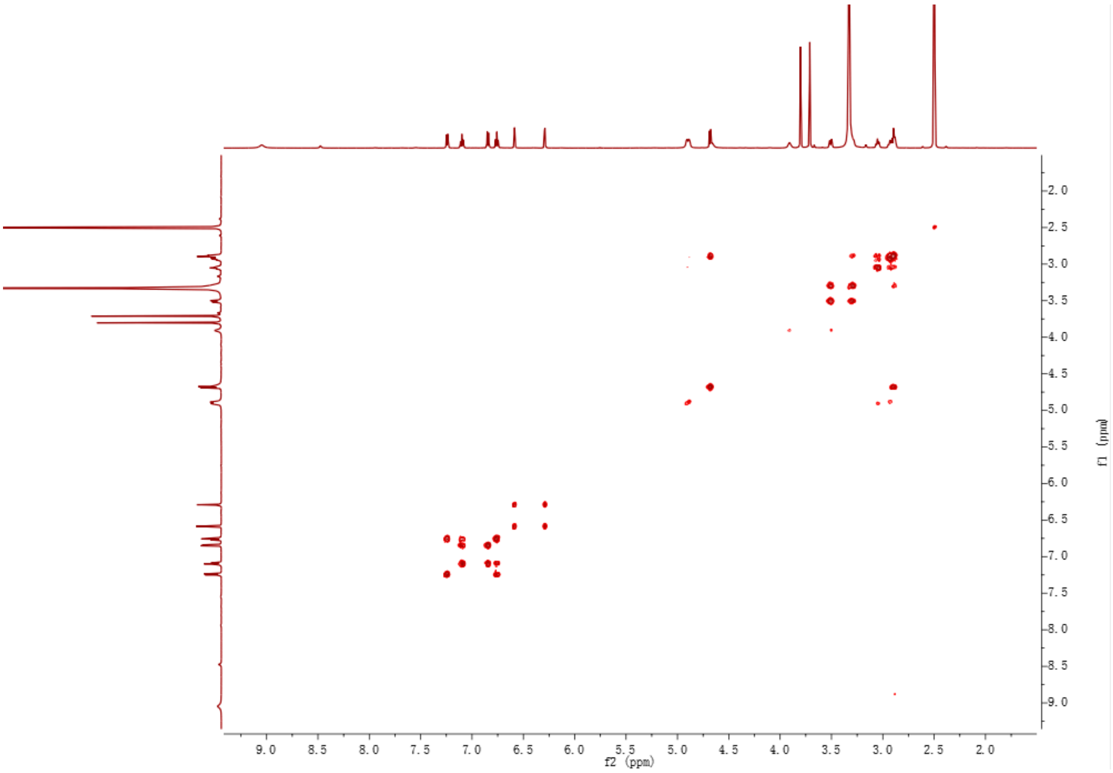

Figure S61. HSQC spectrum of compound 9.

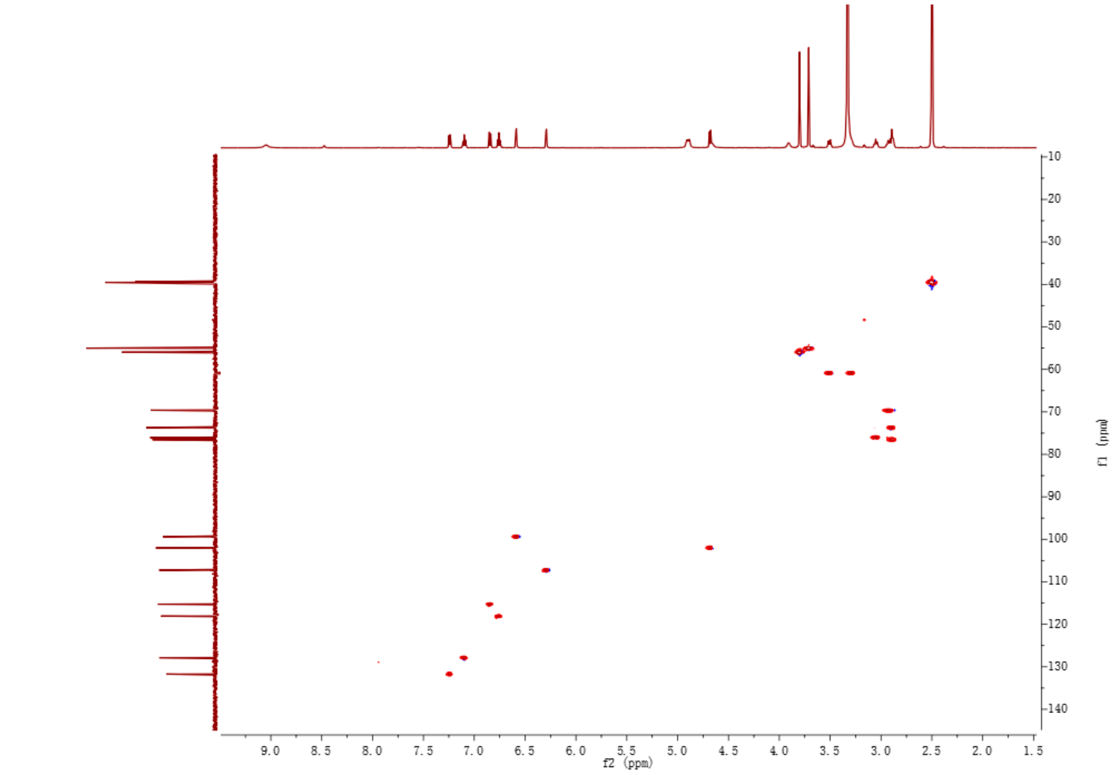

Figure S62. HMBC spectrum of compound 9.

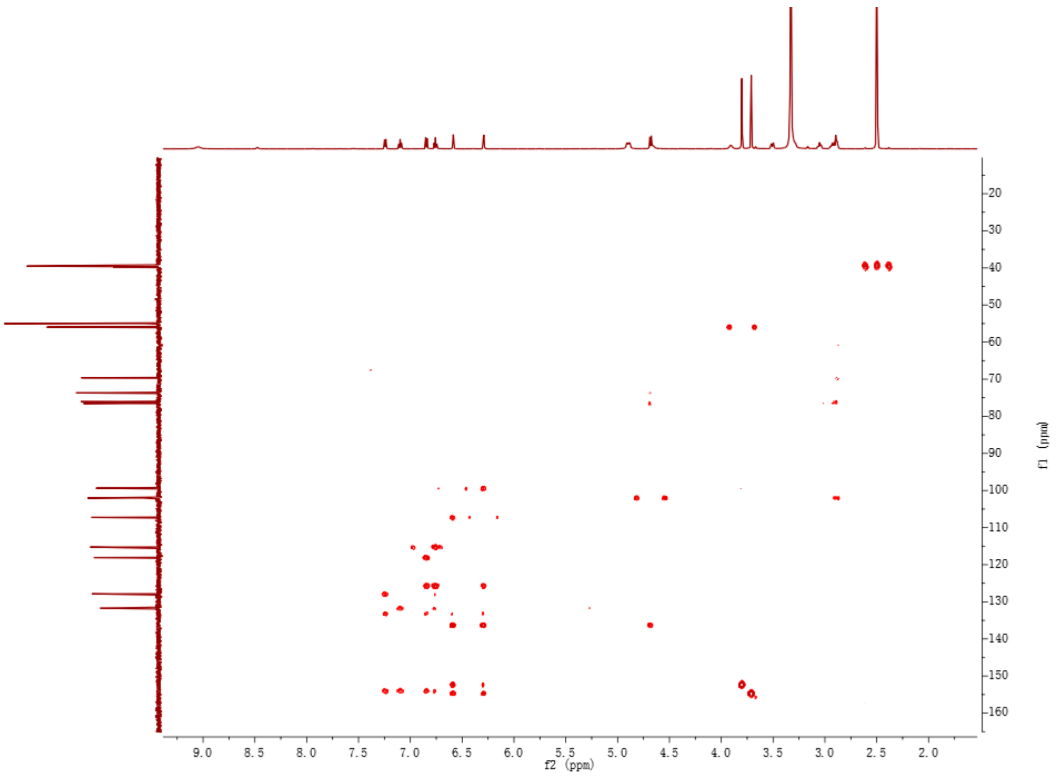

Figure S63. ROESY spectrum of compound 9.

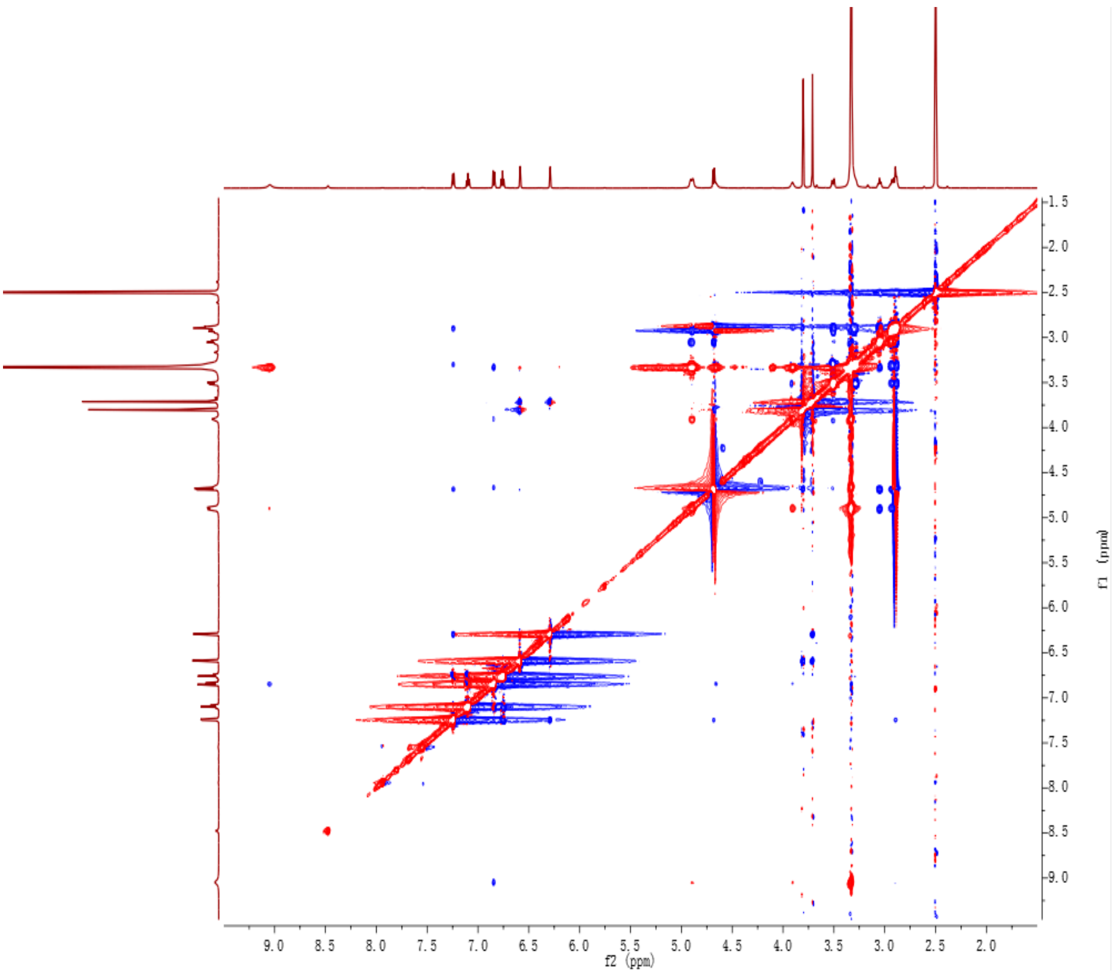

Figure S64. The reversed-phase HPLC spectra of derivatives.

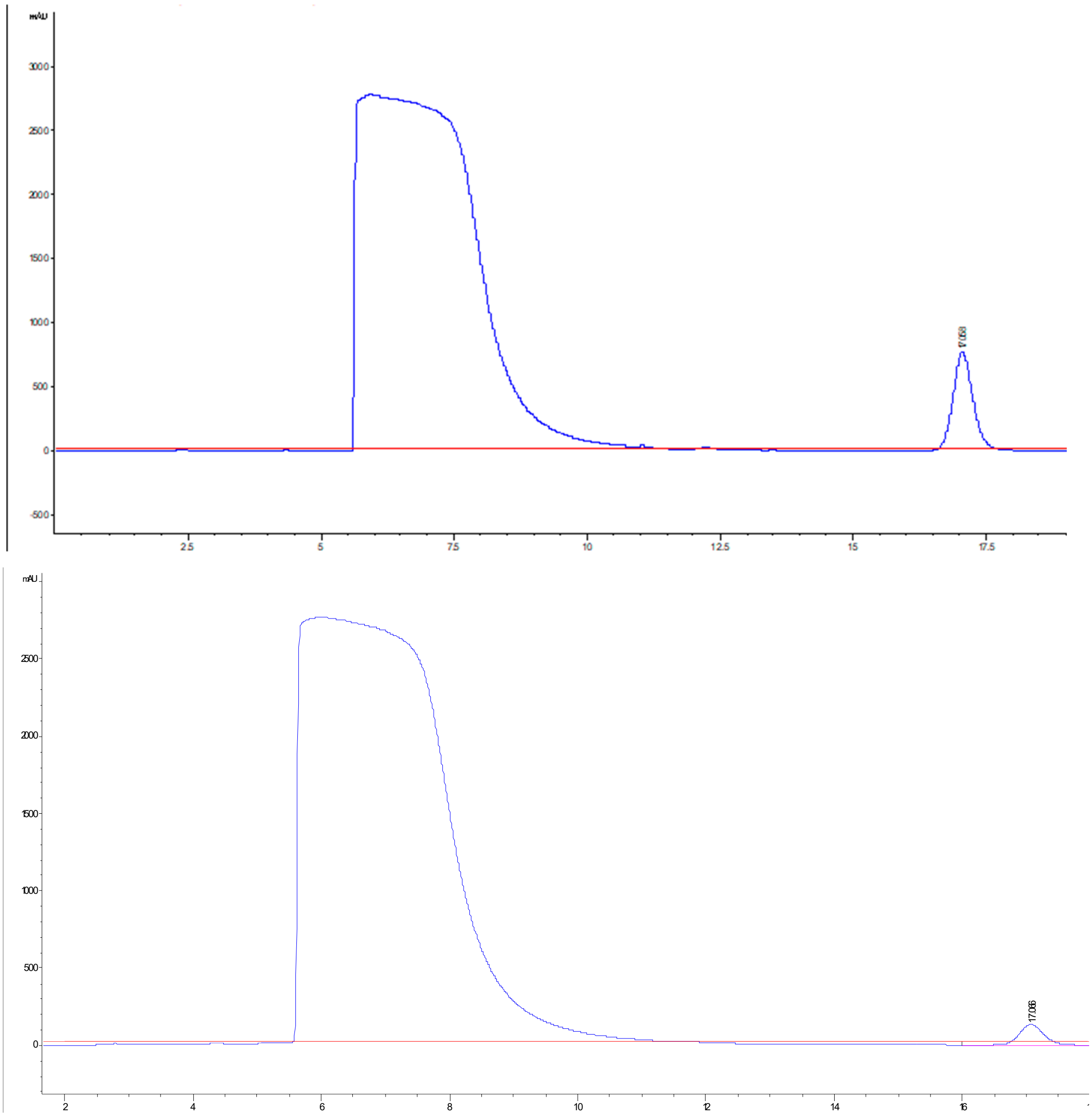

Note:

The mobile phase: CH_3CN-H_2O (21 % within 28 min, isocratic model); flow rate: 1 mL/min; detection: UV (250 nm).
